# Temporally plastic photonic processor for real-time adaptive computing

**DOI:** 10.1186/s43593-026-00139-8

**Published:** 2026-07-23

**Authors:** Lingzhi Luo, Yizhi Wang, Zhiwei Xue, Yanzhi Chen, Chunhui Yao, Senbiao Qin, Peng Bao, Jing Zhang, Kangning Xu, Minjia Chen, Ting Yan, Yuxiao Ye, Liang Ming, Gunther Roelkens, Jianji Dong, Tawfique Hasan, Ian White, Richard Penty, Lu Fang, Qixiang Cheng

**Affiliations:** 1 Electrical Engineering Division, Department of Engineering, University of Cambridge, Cambridge, UK; 2 GlitterinTech Limited, Xuzhou, China; 3 Department of Electronic Engineering, Tsinghua University, Beijing, China; 4 Microsoft Research, Cambridge, UK; 5 Photonics Research Group, Ghent University-IMEC, Ghent, Belgium; 6 Wuhan National Laboratory for Optoelectronics, School of Optical and Electronic Information, Huazhong University of Science and Technology, Wuhan, China; 7 Department of Electronic and Electrical Engineering, University of Bath, Bath, UK

## Abstract

Real-time intelligent systems increasingly require hardware that can adapt continuously to evolving inputs, yet most existing processors rely on static-weight inference, making them vulnerable to distribution shifts and error accumulation in dynamic environments. Although adaptive weight updates can, in principle, address this limitation, their implementation on electronic hardware is hindered by the stability–plasticity trade-off, as well as by the memory wall and clocking bottlenecks that become particularly severe in sequential processing. Here, we present a Temporally Plastic Photonic Processor (TPPP) that enables ultra-fast in situ adaptation by combining multi-timescale photonic kernels with a recursive optical delay memory. The architecture integrates a slow, reconfigurable kernel for stable long-term processing and a fast, dynamic kernel for transient adaptation, enabling time-varying weights to be embedded directly in the photonic domain without repeated electronic memory access. We experimentally validate the TPPP on linear and nonlinear sequential tasks. In both regimes, data-driven temporal plasticity enables the TPPP to outperform conventional static photonic baselines in robustness and accuracy. Under an operation-matched INT8 comparison, scaling analysis projects up to 16 × higher per-operation energy efficiency and up to 100 × lower intrinsic single-pass compute delay than advanced electronic processors, establishing the TPPP as a promising hardware framework for real-time adaptive photonic computing.

**Supplementary Information:**

The online version contains supplementary material available at 10.1186/s43593-026-00139-8.

## Introduction

Real-time intelligent systems increasingly operate in dynamic, data-intensive environments, where both input distributions and required responses evolve continuously over time. Applications ranging from autonomous vehicles to real-time sensing and generative artificial intelligence, therefore, demand not only high throughput and low latency but also the ability to adapt computation on the fly [1–3]. However, most existing hardware still relies on static-weight inference, which is inherently vulnerable to distribution shifts and error accumulation in sequential tasks. As temporal dependence increases, these limitations become more severe because each new prediction depends on evolving internal states and/or previous outputs. Robust computation under changing conditions therefore becomes a fundamental challenge, rather than simply a matter of accelerating inference.

In conventional electronic processors, enabling such adaptive behaviour is costly. Real-time weight updating and recurrent state evolution require frequent memory access, data movement, and synchronization, all of which are constrained by the memory wall, clocking overhead, and thermal limits of digital hardware [4, 5]. These penalties are particularly severe for sequential and autoregressive models, including Recurrent Neural Networks (RNNs), Transformers, and State-Space Models (e.g., Mamba), where temporal dependencies amplify latency and energy consumption [6–8]. Moreover, adaptive learning systems must balance the stability–plasticity trade-off: a processor must remain sufficiently stable to preserve previously learned behaviour, yet sufficiently plastic to respond rapidly to new inputs and environmental changes. Achieving both simultaneously remains difficult in electronic hardware, especially when adaptation must occur in real time.

Integrated photonics offers a promising route beyond these constraints because optical computation naturally provides high bandwidth, low latency, and excellent energy efficiency [9–11]. Recent advances in large-scale photonic integrated circuits have enabled powerful accelerators for matrix operations, neural networks, and high-speed signal processing [12–14]. Yet most existing photonic processors are still designed around static transformations, in which a mathematical kernel is mapped onto fixed hardware and the optical signal flow is repeatedly interrupted by electro-optic conversion for control and memory operations. Emerging recurrent and asynchronous photonic architectures have begun to extend photonic hardware toward sequential information processing, but inference acceleration alone does not solve the broader challenge of adaptation in complex, time-varying environments [15–17]. In practice, photonic systems must also contend with device imperfections, environmental fluctuations, and accumulated analogue errors, making it essential to develop architectures that support dynamic, in-situ adaptation without sacrificing photonic speed advantages [18].

Here, we present the Temporally Plastic Photonic Processor (TPPP), a photonic architecture for real-time adaptive computing in which data-derived operators evolve in situ at each inference step. Unlike conventional static processors, the TPPP operates via a dual-kernel design: a slow, reconfigurable kernel that preserves stable long-term transformations and a fast, dynamic kernel that enables rapid short-term adaptation. Inspired by multi-timescale biological plasticity, this architecture combines thermo-optic and electro-optic control with recursive optical delay memory, allowing temporal state evolution and adaptive processing to remain in the optical domain. Through heterogeneous integration of III–V gain modules on silicon photonics, the processor sustains recurrent low-loss signal circulation while avoiding repeated electronic memory access and the associated latency overhead.

We further show that this architecture supports two broad classes of sequential computation: linear adaptive inference and nonlinear recurrent decision-making. In linear mode, the TPPP accelerates regression by reducing the cost of iterative matrix inversion, as demonstrated through real-time spectral analysis. In nonlinear mode, it implements adaptive autoregressive models, demonstrated through a Time-Adaptive Recurrent Neural Network (TA-RNN) for autonomous navigation. These representative tasks show that the coupled slow and fast kernels enable robust multi-timescale adaptation to evolving inputs, noise, and device imperfections. The resulting system achieves a validated 8 × 8 INT8-encoded operating point (40 Gb/s, 810 fJ/op) and projects up to a 16-fold improvement in per-operation energy efficiency and up to a two-orders-of-magnitude reduction in intrinsic single-pass compute delay relative to advanced electronic processors, establishing the TPPP as a promising hardware framework for real-time adaptive photonic computing.

## Results

### Working principles

#### Temporal plasticity

Sequential processing models can be broadly formulated as:

1 $$\begin{array}{c}{z}_{k}={\mathcal{F}}_{{\Theta}_{k}}\left(k,{x}_{k},{s}_{k-1}\right)\end{array}$$

where ${x}_{k}$ is the runtime input, ${z}_{k}$ is the output at timestep $k$, and ${s}_{k-1}$ contains all the historical information available before timestep $k$. ${s}_{k-1}$ may include previous states or outputs, measured signals, device/environmental states, and previously applied operator settings. ${\Theta}_{k}$ denotes the full set of tunable hardware parameters that define the optical operator ${\mathcal{F}}_{{\Theta}_{k}}$ at timestep $k$. Thus, ${\Theta}_{k}$ configures the operator, while ${\mathcal{F}}_{{\Theta}_{k}}$ is the actual optical mapping executed. Temporal plasticity refers to the ability of ${\Theta}_{k}$ to vary over time, enabling the system to capture dynamic features that are difficult to represent efficiently with a fixed static operator. The time-dependent parameter set ${\Theta}_{k}$ can be obtained through predefined, analytical, or data-driven acquisition mechanisms, as detailed in Supplementary Note 7.

#### TPPP architecture

**Fig. 1:**
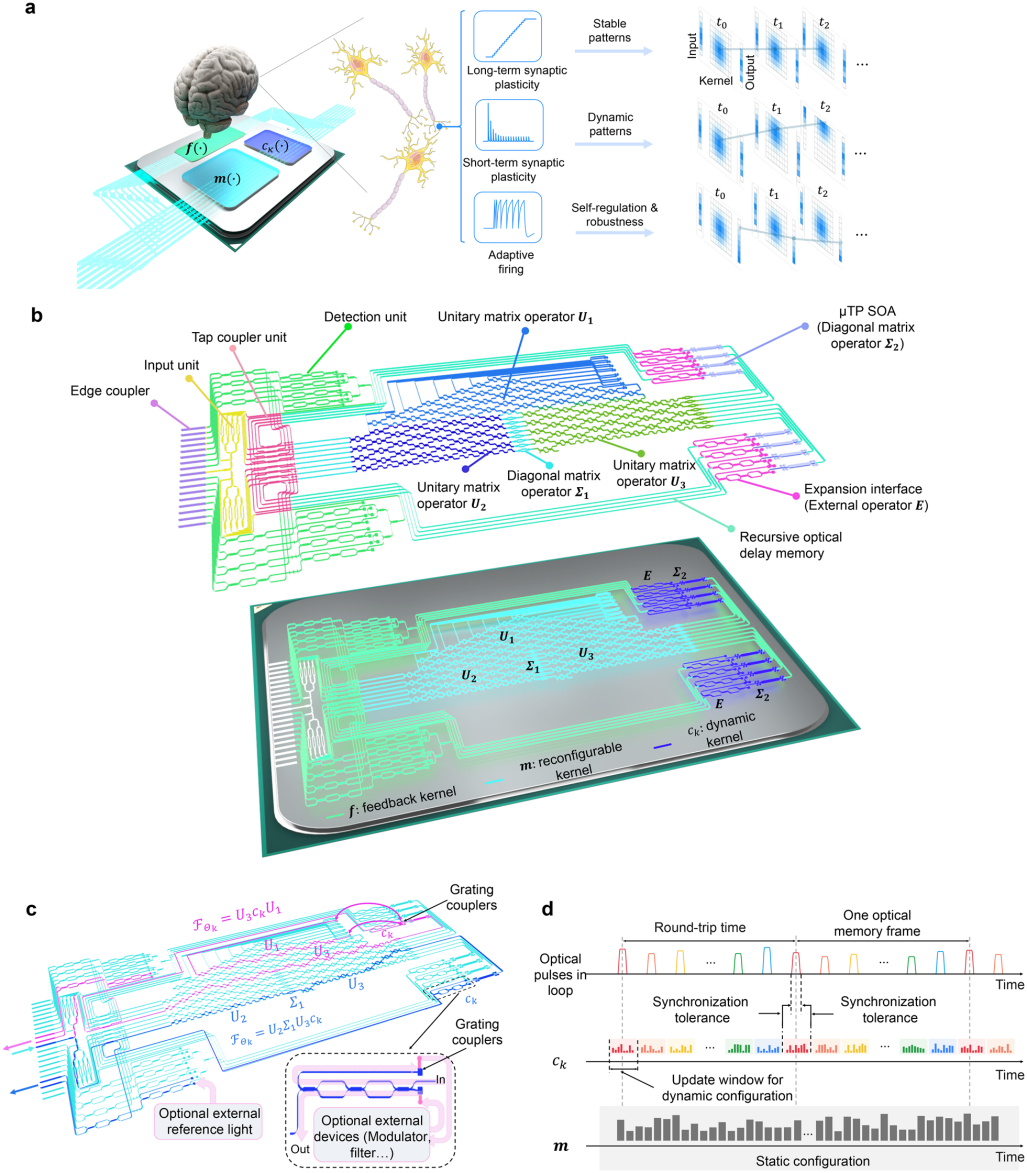
Conceptual illustration and schematic of the temporally plastic photonic processor. **a** Conceptual illustration of temporally plastic photonic processors, inspired by multi-timescale responses of biological systems. **b** Processor architecture and functional components. **c **Light path configurations for channel regulation (blue) and mode reweighting (red). **d** Timing diagram of the temporally plastic photonic processor

In this work, ${\Theta}_{k}$ comprises the control parameters of three unitary operators $\left({U}_{1},{U}_{2},{U}_{3}\right)$, two diagonal matrix operators $\left({\Sigma}_{1},{\Sigma}_{2}\right)$, and an external operator $E$ accessed through an expansion interface. These operators are organized into three biologically inspired kernels (Fig. 1a): a fully reconfigurable kernel $m(\cdot )$, a feedback kernel $f(\cdot )$, and a dynamic kernel ${c}_{k}(\cdot )$. Together, these kernels enable temporal plasticity through mechanisms conceptually analogous to key biological adaptations, including long-term synaptic plasticity (LTP), short-term synaptic plasticity (STP), and adaptive firing dynamics (AFD).

The three unitary operators and ${\Sigma}_{1}$ are implemented using cascaded Mach–Zehnder interferometer (MZI) meshes with thermo-optic (TO) phase shifters, together constituting the fully reconfigurable kernel *m*(·) [19, 20]. The relatively slow TO tuning provides stable and programmable linear information processing, thereby drawing a conceptual analogy to LTP [21]. ${\Sigma}_{2}$ is implemented using an array of micro-transfer-printed ($\mu$TP) InP-based semiconductor optical amplifiers (SOAs), which provide fast electro-optic (EO) modulation. Meanwhile, $E$ is realized by external components connected through the expansion interface. Together, ${\Sigma}_{2}$ and $E$ form the dynamic kernel ${c}_{k}(\cdot )$, supporting functions conceptually analogous to STP and AFD [22–24].

The feedback kernel $f(\cdot )$ is implemented using a tap coupler that routes part of the optical output back to the input as delay memory, while directing the remaining optical signal to the detection unit for monitoring. Detailed descriptions and performance data for each functional unit are provided in Supplementary Note 3.

Figure 1c shows how the long-timescale plasticity of $m(\cdot )$ and the short-timescale plasticity of ${c}_{k}(\cdot )$ are combined within a single optical pathway. With ${c}_{k}(\cdot )$ placed at the output of $m(\cdot )$ (blue path), the TPPP performs channel-wise amplification, suppression, or gating for input/output regulation, signal compensation, and error suppression. With ${c}_{k}(\cdot )$ inserted between two unitary operators inside $m(\cdot )$ (red path), it instead dynamically reweights the internal feature modes. These two configurations are adopted, respectively, in the linear and nonlinear task demonstrations that follow.

Figure 1d shows the timing diagram of the overall system during one complete computation cycle. Within one round-trip time, multiple temporally separated optical pulses are injected into the optical loop using time-division multiplexing, enabling parallel processing of multiple inputs in a single physical loop. Each pulse occupies an individual time slot. These pulses circulate and are retained inside the loop, forming the recursive optical memory. Therefore, one round-trip time defines both the basic computation period and one optical memory frame, while the number of resolvable pulse slots determines the memory capacity.

During the execution of a specific operator, kernel $m(\cdot )$ is configured in advance and remains fixed, providing the static mapping or long-timescale component. In contrast, kernel ${c}_{k}(\cdot )$ acts as a dynamic modulation kernel that is updated synchronously with the arrival of different pulses in the optical memory. It applies pulse-level, time-dependent control to each time slot, allowing different pulses in the same loop to experience different instantaneous weights. Importantly, kernel ${c}_{k}(\cdot )$ does not require exact temporal alignment with each optical pulse. A finite synchronization tolerance is allowed, as the modulation remains effective as long as the update window covers the target pulse. This improves robustness to timing mismatch, jitter, and delay variations.

### System characterization

**Fig. 2:**
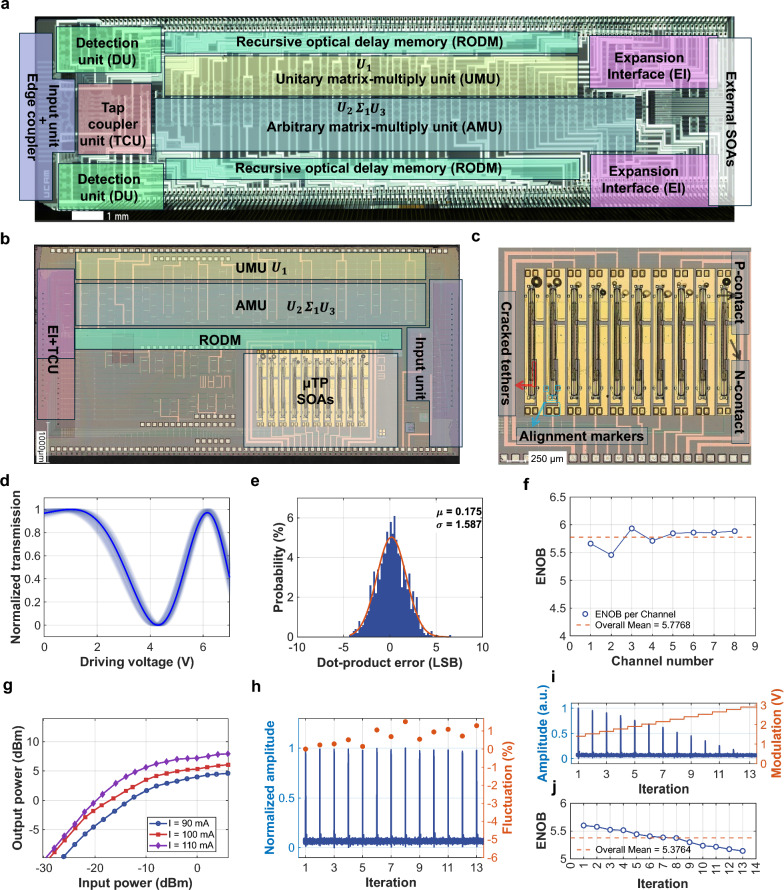
Characterization and performance of temporally plastic photonic processors. Photographs of temporally plastic photonic processor dies on **a** SiN and **b** SOI platforms, with coloured overlays indicating functional regions. **c** Photograph of the micro-transfer-printed SOA array. **d** Transmission characteristics of SiN Mach–Zehnder interferometers (MZIs): the solid line shows the mean transmission across 200 MZIs and the shaded region indicates the range across individual devices. **e** Distribution of single dot-product operation accuracy across 1,000 random input-vector operations: the line shows a Gaussian fit with a mean error of 0.175 LSB and a standard deviation of 1.587 LSB. **f** ENOB measured across eight output channels under INT8 encoding. **g** Input–output curves of the SOA at different injection currents, measured at 19 °C. **h** Optical loop power stability over iterations: optical power per iteration (left axis) and relative fluctuation with respect to the first iteration (right axis). **i** Optical loop modulation synchronized with the round-trip time: modulation signal and corresponding circulating optical amplitude over successive round trips. **j** ENOB as a function of the number of optical circulation cycles

We implemented the system on two complementary platforms: low-loss silicon nitride (SiN) and compact silicon-on-insulator (SOI). The SiN implementation was used to demonstrate the complete all-optical computing link, while the SOI implementation, together with the μTP SOA array (already heterogeneously integrated on SOI), was experimentally characterized at the building-block level. This dual-platform approach validates the portability and scalability of the TPPP architecture across photonic material systems, delineates the associated performance trade-off boundaries, and outlines a clear pathway toward heterogeneously integrated multi-chip systems. Figure 2 presents the comprehensive characterization of the TPPP. The SiN implementation features an on-chip optical loop (Fig. 2a), whilst the SOI variant incorporates micro-transfer-printed (µTP) SOA arrays (Fig. 2b), shown magnified in Fig. 2c. Figure 2d demonstrates exceptional MZI uniformity across 200 SiN devices, with measured average phase-shift efficiency of 12 mW/π. Despite minor device inconsistency, we implemented a systematic calibration method combining photonic functional verification patterns with gradient descent optimization (see Supplementary Note 3.5). After calibration, single-pass operation maintains high precision: dot-product errors average 0.175 LSB ± 1.587 LSB (Fig. 2e). All eight output channels deliver an effective number of bits (ENOB) of ~ 5.78 under INT8 encoding (Fig. 2f).

Response curves under different biases (Fig. 2g) reveal saturation of the SOAs with increasing input power, enabling either path-loss compensation or nonlinear activation by tuning the drive current. Systematic calibration ensures negligible power variation between iterations with fluctuations remaining below 2%, as shown in Fig. 2h. This stability enables precise temporal control, as demonstrated by synchronized loop modulation in Fig. 2i, where circulating optical amplitude can be dynamically attenuated from maximum to zero within each round trip. The multi-iteration performance analysis for successive identity-matrix multiplications is shown in Fig. 2j, revealing gradual ENOB degradation from 5.78 to approximately 5.14 after multiple cycles. This modest reduction stems from unavoidable amplified spontaneous emission (ASE) noise and nonlinear effects introduced by the SOA or erbium-doped fibre amplifier (EDFA) during each circulation, representing a fundamental trade-off between recurrent depth and computational precision.

### Linear task: spectrum-based moisture prediction

We first apply TPPP to a linear learning task for spectrum-based moisture prediction, demonstrating its high accuracy and temporal stability. In this task, the moisture content of flour is inferred from its near-infrared transmission spectrum using a ridge-regression model. The key computational step is to invert a regularized moment matrix ${X}^{T}X+\lambda I$, constructed from the spectral features $X$ and the regularization term $\lambda I$. More details can be found in Methods.

**Fig. 3:**
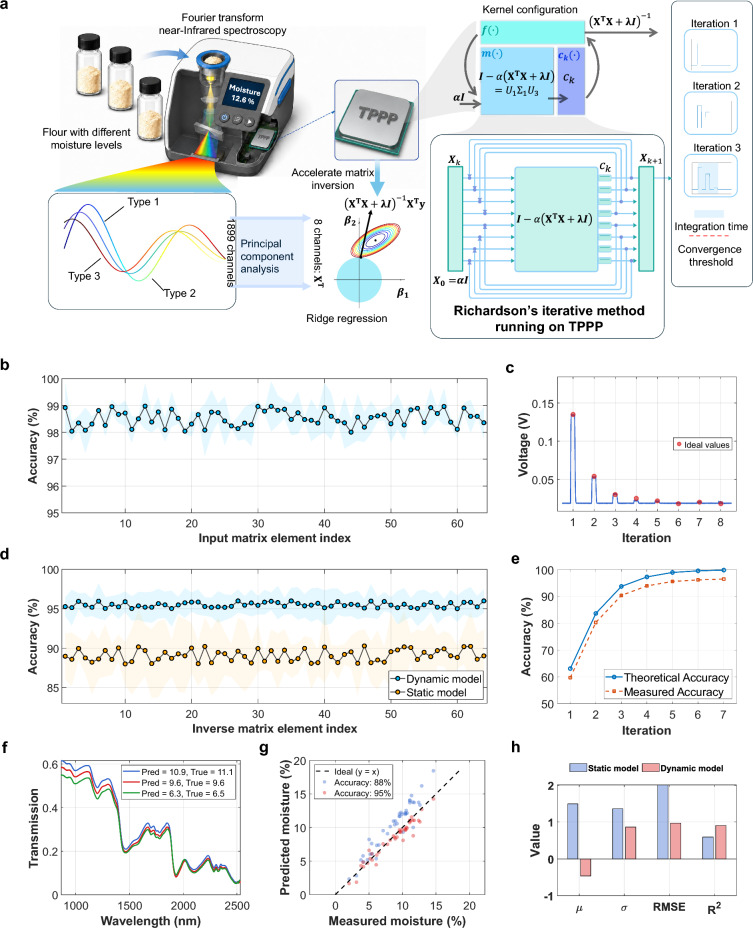
Spectral information processing in linear mode.** a** Schematic overview of Richardson iteration implemented on TPPP hardware for FT-NIR flour moisture prediction. FT-NIR spectra from flour samples with different moisture levels are compressed by PCA and used for ridge-regression-based prediction. The matrix inversion required by ridge regression is reformulated as a Richardson iteration and implemented on TPPP hardware using the linear-mode reconfigurable kernels. **b** Input-matrix encoding accuracy; the shaded region indicates the range across error bars. **c** Convergence of representative matrix elements during numerical inversion. **d** Element-wise accuracy of the inverted matrix with and without dynamic adaptation; the shaded region indicates the range across error bars. **e** Accuracy comparison between theoretical and measured values at each iteration of the Richardson algorithm. **f** FT-NIR transmission spectra for flours with different moisture content, with corresponding predicted and ground-truth moisture values. **g** Comparison of predicted and measured flour moisture for a static model (blue) and with dynamic adaptation (red). **h** Statistical prediction error metrics, including mean error (μ), standard deviation (σ), root mean squared error (RMSE), and coefficient of determination (R^2^)

Figure 3a gives a schematic overview of Richardson iteration implemented on TPPP hardware for flour moisture prediction. To construct this task, near-infrared transmission spectra were measured from flour samples with different moisture contents using a Fourier-transform infrared spectrometer. The original spectra contain 1899 wavelength channels, which are reduced offline via principal component analysis (PCA) preprocessing to eight principal components before being used as the input spectral features $X$. As the dominant computational step of the regression pipeline, the subsequent regularized matrix inversion is implemented and measured directly on the photonic chip, while the final spectrum-prediction and classification results are obtained by combining the measured optical outputs with standard electronic post-processing.

The kernel $m(\cdot )$ is programmed via singular value decomposition (SVD) to implement the iterative update operator as ${\mathrm{U}}_{2}{\Sigma}_{1}{\mathrm{U}}_{3}={\mathrm{I}-\upalpha (\mathrm{X}}^{\mathrm{T}}\mathrm{X}+\uplambda \mathrm{I})$. At its output, the dynamic kernel ${c}_{k}(\cdot)$ applies a data-driven correction, yielding the compensated transformation ${\mathrm{U}}_{2}{\Sigma}_{1}{\mathrm{U}}_{3}{c}_{k}$. Learned from device and environmental calibration data, ${c}_{k}$ adaptively compensates for path-dependent losses, device nonidealities, environmental noise, and channel-dependent signal skew across different optical paths and hardware configurations. The external electro-optic modulator (EOM) contributes to the physical implementation of ${c}_{k}(\cdot)$ by providing a high-speed electro-optic modulation pathway that maps the electronically generated correction signals onto the circulating optical pulse stream. More details can be found in Supplementary Notes 2 and 4.

During operation, the output from each iteration is fed back as the input to the next, allowing the system to progressively converge to the regression solution. With the convergence parameter set to $\alpha =0.5633$, TPPP achieves fast convergence and temporally stable operation under sequential feedback, enabling accurate spectrum-based moisture classification.

The encoding fidelity of the input matrix demonstrates exceptional performance (Fig. 3b) when applied with the dynamic correction methodology through the dynamic kernel. The input encoding system achieves > 98% average accuracy with error fluctuations maintained below 2%, validating the robustness of the encoding scheme. Following initial state injection (*αI*), the system exhibits rapid convergence characteristics (Fig. 3c). Representative matrix elements converge within seven iterations whilst maintaining high computational fidelity, demonstrating the efficiency of the recursive architecture.

Post-convergence analysis reveals significant inversion performance enhancement through dynamic adaptation (Fig. 3d). The static model yields ~ 90% average inversion accuracy with > 5% fluctuation across matrix elements. Dynamic adaptation elevates the inversion average accuracy to ~ 95% whilst suppressing fluctuation to ~ 2%. The iteration-dependent accuracy profile (Fig. 3e) quantifies error accumulation during recursive processing. Despite unavoidable amplifier noise and optical nonlinearities introducing gradual deviation between measured and theoretical values, the final inversion maintains ~ 95% accuracy.

Linear regression implemented on TPPP demonstrates precise moisture sensing capabilities (Fig. 3f). Three representative spectral curves yield predictions deviating < 0.2 from measured values. Extended validation across the complete dataset corroborates these findings. The original data undergoes dimensionality reduction with PCA and has been checked to ensure no significant information is lost. The linear regression achieves strong correlation between TPPP-predicted and professionally measured moisture values. After the regression, a binary classification is applied for more intuitive illustration of performance improvement. By defining a wet/dry threshold to mimic the scenario of a quality check, the samples are classified accordingly. The dynamic adaptation improves accuracy from 88 to 95% (Fig. 3g), demonstrating enhanced discriminative capability. Statistical metrics substantiate the performance gains (Fig. 3h): mean error (μ) improved from 1.496 to -0.46; standard deviation (σ) from 1.36 to 0.864; root mean squared error (RMSE) from 2.011 to 0.972; coefficient of determination (R^2^) from 0.594 to 0.905. These improvements validate TPPP's potential for robust real-world data processing. More details can be found in Supplementary Note 4.

### Nonlinear task: TA-RNN for autonomous driving

We further demonstrate the nonlinear task-solving capability of TPPP through real-time autonomous vehicle control, using a Time-Adaptive RNN (TA-RNN), a newly proposed recurrent model that we co-design with the TPPP hardware architecture. Experiments on our in-house rule-based autonomous-driving simulation dataset validate the system’s ability to support latency-critical decision-making in dynamic environments.

**Fig. 4:**
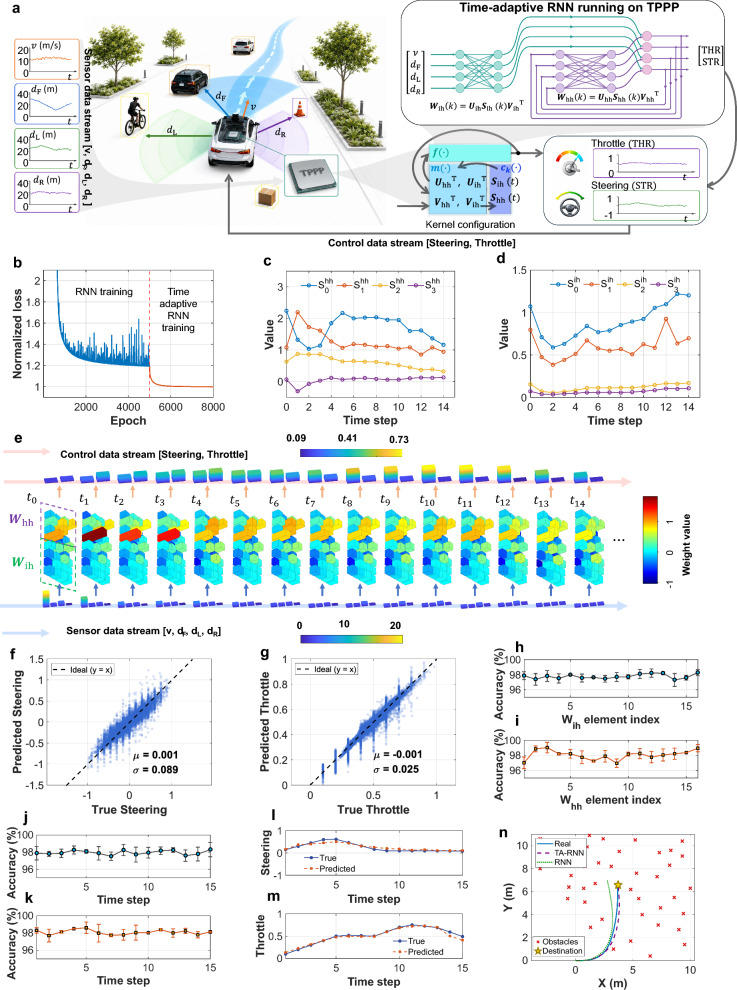
Autonomous driving data processing in nonlinear mode.** a** Schematic overview of a time-adaptive RNN implemented on TPPP hardware for autonomous driving control. Sensor streams, including vehicle speed and front, left and right obstacle distances, are processed by a TA-RNN mapped onto TPPP hardware. The dynamic kernel updates the input-to-hidden and hidden-to-hidden weight matrices over time, enabling real-time prediction of throttle and steering commands. **b** Training loss curves for the TA-RNN showing two stages: standard RNN training followed by fine-tuning of time-varying singular values of the weight matrices. Channel-wise time-adaptive characteristics of the dynamic kernel for **c** ${S}_{\mathrm{h}\mathrm{h}}$ and **d** ${S}_{\mathrm{i}\mathrm{h}}$. **e** Multi-layer TA-RNN with temporal cascading; at each time step, blocks denote combined weight matrices (${W}_{\mathrm{i}\mathrm{h}}$ and ${W}_{\mathrm{h}\mathrm{h}}$), with sensor inputs (*v*, ${d}_{\mathrm{F}}$, ${d}_{\mathrm{L}}$, ${d}_{\mathrm{R}}$) and control outputs (steering, throttle). **f** Predicted versus true steering with Gaussian-fit error statistics (μ = 0.001, σ = 0.089). **g** Predicted versus true throttle with Gaussian-fit error statistics (μ = − 0.001, σ = 0.025). **h** Static encoding accuracy for ${W}_{\mathrm{i}\mathrm{h}}$. **i** Static encoding accuracy for ${W}_{\mathrm{h}\mathrm{h}}$. **j** Mean encoding accuracy over time steps for ${S}_{\mathrm{i}\mathrm{h}}$. **k** Mean encoding accuracy over time steps for ${S}_{\mathrm{h}\mathrm{h}}$. **l** Steering command and **m** throttle command computed by the TPPP and the corresponding ground-truth values. **n** Driving trajectory comparison between ground truth and predictions from a conventional RNN and a TA-RNN

Figure 4a presents a schematic overview of a TA-RNN implemented on TPPP hardware for autonomous driving control. The vehicle dynamics follow a simplified Ackermann kinematics model. It continuously monitors four sensory inputs: current velocity (*v*) and obstacle distances across three 60° sectors: front (${d}_{\mathrm{F}}$), left (${d}_{\mathrm{L}}$), and right (${d}_{\mathrm{R}}$). The TA-RNN processes these inputs to generate real-time control commands: throttle and steering. Obstacles dynamically populate the environment, creating a challenging navigation scenario requiring rapid adaptation. The TA-RNN architecture and TPPP kernel configuration are detailed in Fig. 4a (right). The four-dimensional sensor data (*v*, ${d}_{\mathrm{F}}$, ${d}_{\mathrm{L}}$, ${d}_{\mathrm{R}})$ enters through weight matrix ${W}_{\mathrm{i}\mathrm{h}}$, whilst hidden states from previous time steps propagate via ${W}_{\mathrm{h}\mathrm{h}}$. Following the fully connected layers, the nonlinear activation is implemented using EDFA, which provides a bias-tunable optical nonlinear transfer function. The activated optical outputs are then mapped to the control commands and updated hidden states.

Here, both ${W}_{\mathrm{i}\mathrm{h}}$ and ${W}_{\mathrm{h}\mathrm{h}}$ are implemented via SVD: ${W}_{\mathrm{i}\mathrm{h}}={U}_{\mathrm{i}\mathrm{h}}{S}_{\mathrm{i}\mathrm{h}}{V}_{i\mathrm{h}}^{T},{W}_{\mathrm{h}\mathrm{h}}={U}_{\mathrm{h}\mathrm{h}}{S}_{\mathrm{h}\mathrm{h}}{V}_{\mathrm{h}\mathrm{h}}^{T}.$ The fast diagonal matrix operator in the dynamic kernel ${c}_{k}(\bullet )$, which encodes ${S}_{\mathrm{i}\mathrm{h}}$ and ${S}_{\mathrm{h}\mathrm{h}}$, is inserted between the corresponding unitary matrix operators, $\left({U}_{\mathrm{i}\mathrm{h}},{V}_{i\mathrm{h}}^{T}\right)$ and $\left({U}_{\mathrm{h}\mathrm{h}},{V}_{\mathrm{h}\mathrm{h}}^{T}\right)$. This configuration allows the internal feature modes to be dynamically reweighted. Consequently, the singular-value matrices ${S}_{\mathrm{i}\mathrm{h}}$ and ${S}_{\mathrm{h}\mathrm{h}}$ are adapted at each time step, enabling temporal modulation of the recurrent dynamics (see Supplementary Note 2). The static and dynamic weights are obtained through a two-stage offline training procedure (Fig. 4b). In Stage 1, a conventional RNN is trained to establish baseline task performance. In Stage 2, only the temporal modulation parameters associated with ${S}_{\mathrm{i}\mathrm{h}}$ and ${S}_{\mathrm{h}\mathrm{h}}$ are fine-tuned, enabling task-specific temporal adaptation while preserving the previously learned recurrent representations and mitigating catastrophic forgetting. The training curves show that the conventional RNN reaches a performance plateau after approximately 5,000 epochs, whereas the temporal adaptation stage provides a further 16% reduction in loss. This indicates that the time-dependent diagonal modulation terms introduce useful modelling capacity beyond a fixed-weight RNN. TA-RNN is therefore a model-level contribution rather than merely a hardware-mapping framework: it introduces a structured temporal-adaptation mechanism that reweights learned temporal modes over a stable static backbone, adding time-dependent modelling capacity without retraining the full recurrent weight matrices. TA-RNN therefore provides an efficient and structured approach to temporal adaptation, distinct from standard RNNs with fixed weights, generic time-varying RNNs with unconstrained time-varying matrices, and reservoir computing with a largely fixed recurrent backbone.

Post-training analysis (Figs. 4c, d) reveals the temporal evolution of diagonal matrices ${{\boldsymbol{S}}}_{\mathrm{h}\mathrm{h}}$ and ${{\boldsymbol{S}}}_{\mathrm{i}\mathrm{h}}$. These adaptive parameters dynamically couple real-time sensor data with hidden states, extracting time-varying features essential for navigation in changing environments.

The temporal cascade architecture (Fig. 4e) effectively implements a multi-layer deep neural network, with each layer processing one timestep. Square blocks represent combined weight operations (${{\boldsymbol{W}}}_{\mathrm{i}\mathrm{h}}$ and ${{\boldsymbol{W}}}_{\mathrm{h}\mathrm{h}}$) realized through SVD-based photonic kernels. The bottom stream carries sensor inputs whilst the top stream generates control commands.

Model performance exhibits exceptional accuracy across both control outputs (Figs. 4f, g). Steering predictions cluster tightly along the diagonal with minimal dispersion, even at extreme values (± 1). Gaussian fitting yields near-zero bias (μ = 0.001) with standard deviation σ = 0.089, confirming robust directional control. Throttle predictions maintain superior linearity across the operational range, with highest fidelity in the critical mid-range (0.3–0.7) where most driving occurs. Statistical analysis reveals μ = − 0.001 and σ = 0.025, essential for smooth velocity regulation.

After offline training, only the inference-stage recurrent computation is mapped onto the TPPP system and experimentally carried out through the all-optical loop. TPPP implementation requires precise encoding of static weight matrices ${{\boldsymbol{W}}}_{\mathrm{i}\mathrm{h}}$ and ${{\boldsymbol{W}}}_{\mathrm{h}\mathrm{h}}$ (Figs. 4h, i) alongside dynamic modulation parameters ${{\boldsymbol{S}}}_{\mathrm{i}\mathrm{h}}$ and ${{\boldsymbol{S}}}_{\mathrm{h}\mathrm{h}}$ (Figs. 4j, k). All components achieve ~ 98% encoding accuracy, establishing a robust foundation for recurrent operations. This high fidelity translates directly to control performance, with measured steering (Fig. 4l) and throttle (Fig. 4m) commands demonstrating excellent agreement with target values.

Navigation trajectory comparison (Fig. 4n) reveals the transformative impact of temporal adaptation. TA-RNN achieves < 5% trajectory prediction error. More details can be found in Supplementary Note 5. These results validate the photonic TA-RNN for latency-sensitive applications in ultra-dynamic environments.

## Discussion

**Fig. 5:**
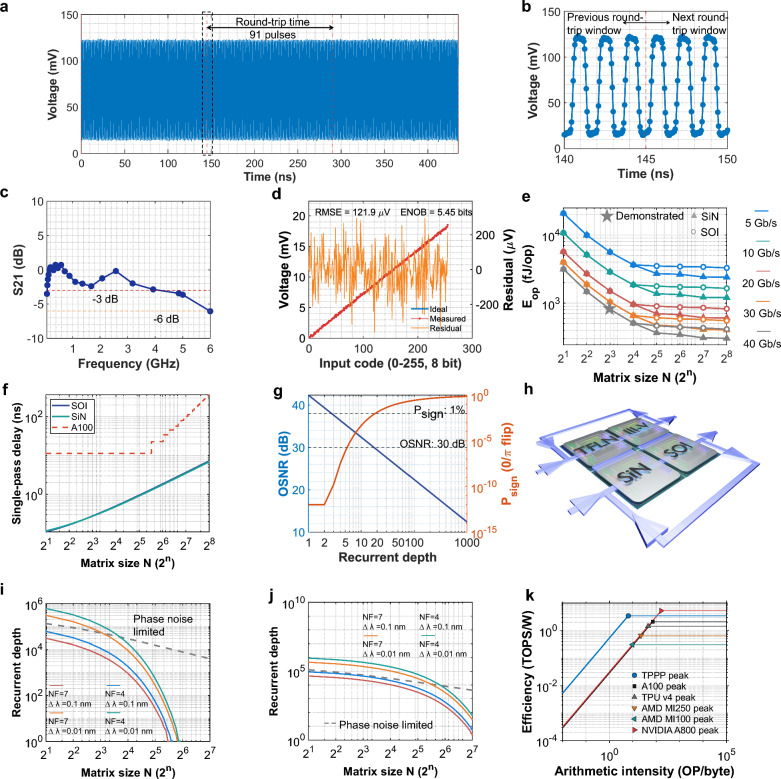
Scalability and performance comparison.** a** Time-domain waveform showing 91 pulse slots within a $\sim 145$ ns round-trip window. **b** Zoom-in near the round-trip boundary. **c** Measured ${S}_{21}$ response of the optoelectronic TPPP system. **d** Computational accuracy at a 5 GHz parallel-task rate with INT8. **e** Energy per operation (${E}_{\mathrm{o}\mathrm{p}}$) at different input data rates for SOI and SiN platforms versus MZI matrix size under INT8 precision. **f** Raw single-pass compute delay versus matrix size under INT8 precision. Solid lines show SOI and SiN delays; the dashed line shows the operation-matched A100 raw compute-delay estimate. **g** Optical signal-to-noise ratio (OSNR) and bit-flip probability versus recurrent depth. **h** Schematic illustration of multi-chiplet scalability architecture. **i** Maximum recurrent depth limited by noise accumulation for the SOI platform at noise figures (NF) of 4 dB and 7 dB, with filter bandwidths of 0.1 nm and 0.01 nm. **j** Maximum recurrent depth limited by noise accumulation for the SiN platform at noise figures of 4 dB and 7 dB, with filter bandwidths of 0.1 nm and 0.01 nm. **k** Compute efficiency comparison based on the Roofline model under INT8 precision

Scaling the TPPP from proof-of-concept to commercial systems requires co-optimizing the matrix size of matrix-multiply unit, energy efficiency, and recurrent depth. As matrices grow, propagation loss becomes limiting and typically requires SOA-based restoration. This overhead is mitigated by a photonic advantage: compute-while-propagating. Optical waveforms are processed continuously as they traverse the device, allowing new inputs to enter before prior ones exit, avoiding global clocking, deep pipelines, and scheduling stalls typical of GPUs. Consequently, biased SOAs and weight-retention circuits are reused in a streaming regime, and total power is dominated by static bias rather than per-operation rate. Time-division multiplexing (TDM) is therefore a natural match for the TPPP architecture. It fully exploits compute-while-propagating, since computation can proceed so long as time-domain slots remain cleanly resolvable, and it simultaneously fills the otherwise idle intervals within the optical delay memory. These advantages are characterized experimentally in Fig. 5. The measured time-domain waveform resolves 91 pulse slots within a single ~ 145 ns round-trip window (Fig. 5a), and a zoom-in near the round-trip time boundary confirms that the optical delay memory preserves pulse fidelity across loop closure, supporting robust temporal storage (Fig. 5b). At the system level, the complete optoelectronic TPPP chain exhibits a 3 dB bandwidth of ~ 5 GHz, which sets the achievable per-slot data rate (Fig. 5c). Operating at this 5 GHz parallel-task rate under INT8 encoding precision, iterative multiplication by identity matrices of varying amplitude yields an effective number of bits (ENOB) of approximately 5.45 for the end-to-end system (Fig. 5d). The demonstrated operating speed is currently limited by the off-chip modulation, synchronization, and detection chain. At higher modulation rates, finite bandwidth, timing jitter, pulse-to-modulation misalignment, and inter-symbol interference reduce the end-to-end signal fidelity, leading to ENOB degradation and lower computational accuracy.

The metrics in Fig. 5a–d are obtained directly from measurements on the demonstrated TPPP hardware. The subsequent analyses (Fig. 5e–k) extend these calibrated experimental observations through analytical and architectural modelling under explicitly stated assumptions, with full derivations and parameter values provided in Supplementary Note 6. Building on this throughput and accuracy, Fig. 5e quantifies the amortization of energy per operation with increasing data rate. The curves are extrapolated from an experimentally validated operating point (8 × 8 matrices at 40 Gb/s, ${E}_{\mathrm{o}\mathrm{p}}=$ 810 fJ/op) using the measured static- and dynamic-power contributions. All system contributions—laser power, SOA bias, weight retention, modulator encoding, receiver chains, and supporting electronics—are included, and the per-operation energy decreases monotonically with rate as the dominant static-power terms are spread across a greater number of operations. Larger matrices initially improve efficiency through higher compute density; beyond 64 × 64, however, the required amplifier count and weight-retention overhead produce an efficiency plateau that defines application-specific optima.

The raw single-pass compute delay increases with matrix dimension (Fig. 5f). For a fair operation-matched comparison, the A100 reference is estimated from serialized Tensor Core tiles required to perform the same number of MAC operations (See Supplementary Note 6.4). The SOI and SiN MZI meshes provide approximately one order of magnitude lower delay on average across the evaluated matrix-size range, with a maximum reduction approaching two orders of magnitude. Recurrent operation is limited by noise accumulation: ASE degrades optical signal-to-noise ratio (OSNR) and longer paths increase phase noise, risking sign errors in real-valued MZI encodings (Fig. 5g). With current parameters, 30 dB OSNR limits depth to 17 iterations, and keeping phase-flip probability below 1% permits 18 iterations. These constraints motivate a heterogeneous multi-chiplet approach (Fig. 5h): SiN for ultra-low-loss routing, SOI for high-density integration, lithium niobate for efficient EO modulation, and heterogeneous III–V gain to compensate loss in deep recurrent networks. Figure 5i–j map operating boundaries: phase noise dominates beyond ~ 10^4^ iterations, ASE constrains practical SOI recurrence at 64 × 64, while SiN sustains dozens of iterations at 128 × 128. Narrower filtering and lower amplifier noise figures would extend these limits. Roofline analysis (Fig. 5k) places TPPP favourably against electronic accelerators. It reaches 3.3 TOPS/W at arithmetic intensity ~ 6.39 OP/byte, where memory bandwidth constrains conventional processors.

Building on these measurements, operation-matched scaling analyses project up to 16 × higher per-operation energy efficiency (~ 300 fJ per operation) and 100 × lower intrinsic compute latency (~ ns-scale) than advanced electronic processors operating under INT8 encoding, with experimentally measured ENOB of ~ 5.78, establishing the TPPP as a promising hardware framework for real-time adaptive photonic computing.

In conclusion, we demonstrate a TPPP that reimagines photonic computing by combining multi-timescale modulation kernels. TPPP unlocks the hardware’s ability to manipulate temporal dynamics. Operation-matched scaling analyses, anchored to the experimentally validated operating point (8 × 8 matrices, INT8 encoding, 40 Gb/s, 810 fJ/op), project up to a 16-fold improvement in per-operation energy efficiency and up to a 100-fold reduction in intrinsic single-pass compute delay relative to advanced electronic processors operating in the same INT8 regime.

## Methods

### Richardson’s iterative method

Considering a simple linear system of $AX=B$, the solution can be numerically approximated with a gradient descent process, often known as Richardson’s iterative method, as shown in Eq. (2) [25, 26]:

2 $$\begin{array}{c}{{\boldsymbol{X}}}_{(k+1)}={{\boldsymbol{c}}}_{k} \cdot[\left({\boldsymbol{I}}-\alpha {\boldsymbol{Y}}\right){{\boldsymbol{X}}}_{k}], (k=\mathrm{0,1},2,\dots , {{\boldsymbol{X}}}_{0}=\alpha I)\end{array}$$

where ${\boldsymbol{Y}}$ is the target matrix, ***I*** is the identity matrix and $\alpha$ sets the convergence rate. ${{\boldsymbol{X}}}_{(k+1)}$ and ${{\boldsymbol{X}}}_{k}$ are the output of the $(k+1)$-th and $k$-th iterations, respectively, ${{\boldsymbol{c}}}_{{\boldsymbol{k}}}$ is the active correction term that adaptively compensates for path-dependent losses, device nonidealities, environmental noise, and channel-dependent signal skew across different optical paths and hardware configurations. By encoding the matrix ${\boldsymbol{I}}-\alpha {\boldsymbol{Y}}$ in $m(\cdot )$, and injecting the initial state ${{\boldsymbol{X}}}_{0}=\alpha {\boldsymbol{I}}$, the optical loop generates successive Neumann-series terms. The accumulated sum of the corrected optical outputs over iterations then converges to the desired solution.

### Time-adaptive RNN

The time-adaptive RNN operating in nonlinear mode of TPPP can be expressed as Eq. (3):

3 $$\begin{array}{c}{a}_{k}={\sigma}_{k}\left({{\boldsymbol{W}}}_{\mathrm{h}\mathrm{h}}(k){a}_{k-1}+{{\boldsymbol{W}}}_{\mathrm{i}\mathrm{h}}(k){v}_{k}+{b}_{k}\right), k=1,\dots ,N\end{array}$$

where ${a}_{k}$ denotes the result vector after layer $k$ with ${a}_{0}$ as input states*.* ${{\boldsymbol{W}}}_{\mathrm{h}\mathrm{h}}$ denotes the hidden-to-hidden weight matrix, and ${{\boldsymbol{W}}}_{\mathrm{i}\mathrm{h}}$ denotes the input-to-hidden weight matrix for the layer-specific injected input. ${v}_{k}$ is the external input unique to layer *k*. ${\sigma}_{k}$ is the non-linearity for layer k, ${b}_{k}$ is the bias.

The modified RNN, termed TA-RNN, leverages the SVD theory where an arbitrary linear transformation can be regarded as the cascade of two unitary transformations and a diagonal one. For predominantly static connectivity, minute changes to small parts can be used for optimizing the system without the need to modify the entire transformation. Thus, the two weight matrices ${{\boldsymbol{W}}}_{\mathrm{i}\mathrm{h}}$ (input-to-hidden) and ${{\boldsymbol{W}}}_{\mathrm{h}\mathrm{h}}$ (hidden-to-hidden) of the TA-RNN are given in Eq. (4).

4 $$\begin{array}{c}{{\boldsymbol{W}}}_{\mathrm{i}\mathrm{h}}={{\boldsymbol{U}}}_{\mathrm{i}\mathrm{h}} {{\boldsymbol{S}}}_{\mathrm{i}\mathrm{h}} {{{\boldsymbol{V}}}_{\mathrm{i}\mathrm{h}}}^{T}, {{\boldsymbol{W}}}_{\mathrm{h}\mathrm{h}}={{\boldsymbol{U}}}_{\mathrm{h}\mathrm{h}}{{\boldsymbol{S}}}_{\mathrm{h}\mathrm{h}}{{{\boldsymbol{V}}}_{\mathrm{h}\mathrm{h}}}^{T}\end{array}$$

Crucially, singular values evolve temporally according to:

5 $$\begin{array}{c}{{\boldsymbol{S}}}_{\mathrm{i}\mathrm{h}}\left(k\right)={{\boldsymbol{S}}}_{\mathrm{i}\mathrm{h}} + \lambda \times \Delta {{\boldsymbol{S}}}_{\mathrm{i}\mathrm{h}}\left(k\right), {{\boldsymbol{S}}}_{\mathrm{h}\mathrm{h}}\left(k\right)={{\boldsymbol{S}}}_{\mathrm{h}\mathrm{h}} + \lambda \times \Delta {{\boldsymbol{S}}}_{\mathrm{h}\mathrm{h}}\left(k\right)\end{array}$$

By employing slowly changing connectivity in $m(\cdot )$ and rapidly changing dynamics in ${c}_{k}(\cdot )$, the nonlinear mode TPPP allows for efficient and robust processing of sequential information compared to standard photonic-based RNNs.

### Chip fabrication

We fabricate the silicon nitride (SiN) photonic chip at CUMEC using the CSiN300 process on 200-mm silicon wafers. The device stack comprises a 4.4-µm buried oxide, a 300-nm SiN waveguide layer, and a 3.5-µm oxide cladding. We define optical components through complete etching of the SiN layer using 248-nm deep-ultraviolet lithography. A 600-nm titanium nitride layer provides resistive heating for thermo-optic phase control, with etched thermal-isolation trenches enhancing modulation efficiency.

We fabricate the silicon-on-insulator (SOI) photonic chip using IMEC's iSiPP50G platform on 200-mm SOI wafers featuring a 2-µm buried oxide and 220-nm crystalline-silicon device layer. Three etch depths—220 nm (full etch), 150 nm (shallow etch), and 70 nm (rib etch)—define the photonic components using 193-nm immersion lithography.

We employ micro-transfer printing with polydimethylsiloxane (PDMS) stamps to heterogeneously integrate prefabricated III–V semiconductor optical amplifiers (SOAs) onto silicon photonics [27, 28]. This technique lifts microscale SOA "coupons" from native InP substrates and precisely places them onto target silicon chips.

We fabricate SOAs on 2-inch InP wafers grown by metalorganic vapour-phase epitaxy (MOVPE). Selective wet etching of a sacrificial release layer liberates devices whilst maintaining tethers to the source wafer. Rapid retraction of a laminated PDMS stamp breaks these tethers, transferring coupons to the stamp's surface. The stamp's optical transparency enables alignment with ± 1-µm accuracy. We complete transfer by laminating the loaded stamp onto the target wafer pre-coated with divinylsiloxane-bisbenzocyclobutene (DVS-BCB) adhesive. Slow retraction with lateral shear motion releases coupons onto the silicon substrate. Post-transfer metallisation electrically connects the III–V devices to existing silicon bond pads.

For seamless integration on IMEC's iSiPP50G platform, we etch localised back-end recesses exposing buried silicon waveguides. Each 40-µm-wide, 1.2-mm-long SOA incorporates dual 180-µm adiabatic tapers for efficient optical coupling. A bilayer polysilicon/crystalline-silicon waveguide (160 nm/220 nm) beneath the SOA mediates vertical coupling, with an additional taper transitioning into standard 220-nm silicon wire waveguides.

### Measurement setup

A narrow-linewidth tunable laser (Thorlabs) delivers 1550-nm light at 10 dBm output power. Polarisation controllers (Thorlabs FPC030) align the input polarisation to the chip. The chips are bonded to metal heatsinks. Wire bonds connect chip electrodes to the PCB. A thermoelectric cooler maintains thermal stability, with its cold side attached to the heatsink's bottom surface and hot side to the thermal baseplate. An embedded negative-temperature-coefficient thermistor monitors heatsink temperature. On-chip expansion interfaces connect to auxiliary devices. Intensity modulators and phase modulators (Thorlabs LNA6213, LN65S-FC), driven by arbitrary-waveform generators (Tektronix AWG5204) and amplified by RF amplifiers (SHF S807Cs), generate ns-scale optical pulses for computations. Isolators (Thorlabs IO-G-1550-APC) are inserted in the optical paths to suppress back-reflected light from propagating back into the upstream components. Tunable delay lines and AWG are employed to synchronize the pulse arrival times. The phase-stabilization setup consists of equal-length reference and measurement fibers mounted on an air-floated optical bench, with temperature-controlled operation to reduce environmental drift. A weak pilot/reference signal is used for phase monitoring. The detected interference signal is digitized by an AD9361 RF transceiver and processed by a ZYNQ-7020 FPGA, which generates a feedback signal. After RF amplification, this signal drives phase modulators (Thorlabs LN65S-FC) to actively stabilize the optical phase. Balanced photodetectors (Thorlabs BDX-series, 0–5 GHz bandwidth) receive the output signals through matched optical delay lines. A 4-channel oscilloscope (Teledyne LeCroy WavePro 804HD) records outputs. EDFAs (Connect MARS) and SOAs compensate optical losses and can also be used as nonlinear activation elements, where photodetection-based feedback adjusts the EDFA pump current or SOA bias current to control the gain transfer function. Optical filters (WL Photonics, 0.1-nm 3-dB bandwidth) suppress ASE noise. A Python-controlled microcontroller (STM32) operates a 450-channel DAC system based on AD5370 chips via SPI protocol. Supplementary Notes 1–5 detail specific configurations for different operation modes.

## Supplementary Information

Additional file 1.

## Data Availability

All data supporting this study are included within the main text and/or Supplementary Information. The dataset is available at: https://github.com/Lingzhi-CIMCS/TPPP. Additional inquiries regarding the data can be directed to the corresponding author.
