# Supplementary material for: Temporally plastic photonic processor for real-time adaptive computing
Source: eLight. 2026 Jul 23;6(1):24. doi: 10.1186/s43593-026-00139-8 (PMC13396080; doi:10.1186/s43593-026-00139-8)
Supplement: Supplementary file 1 — Additional file 1. [file 43593_2026_139_MOESM1_ESM.pdf]

Supplementary Information for:
Temporally Plastic Photonic Processor for Real-Time Adaptive Computing

Author Information

Lingzhi Luo[1,2†], Yizhi Wang[1 †], Zhiwei Xue[3], Yanzhi Chen[4], Chunhui Yao[1,2], Senbiao Qin[5], Peng Bao[1], Jing Zhang[5], Kangning Xu[2], Minjia Chen[1], Ting Yan[2], Yuxiao Ye[2], Liang Ming[2], Gunther Roelkens[5], Jianji Dong[6], Tawfique Hasan[1], Ian White[7], Richard Penty[1], Lu Fang[3, *], Qixiang Cheng[1,2 *]

1. Electrical Engineering Division, Department of Engineering, University of Cambridge, Cambridge, U.K.
2. GlitterinTech Limited, China
3. Department of Electronic Engineering, Tsinghua University, Beijing, China
4. Microsoft Research, Cambridge, U.K.
5. Photonics Research Group, Ghent University-IMEC, Ghent, Belgium
6. Wuhan National Laboratory for Optoelectronics, School of Optical and Electronic Information, Huazhong University of Science and Technology, Wuhan, China
7. Department of Electronic and Electrical Engineering, University of Bath, Bath, U.K.

[*] Corresponding authors: Lu Fang (fanglu@tsinghua.edu.cn), Qixiang Cheng (qc223@cam.ac.uk)

†These authors contributed equally

Table of Contents

Author Information ... 1
Table of Contents ... 2
Supplementary Note 1: Linear-mode computing ... 4
1.1 The Richardson Method ... 4
1.2 Error Propagation, Convergence and Accuracy ... 5
1.3 Power Splitting and Gain Compensation in Iterative Operation ... 6
1.4 Computational Efficiency Analysis ... 6
Supplementary Note 2: Nonlinear-mode computing ... 8
2.1 Time adaptive RNN ... 8
2.2 Training Strategy ... 8
Supplementary Note 3: Functional parts characterization ... 12
3.1 Fiber-to-chip couplers ... 12
3.2 Expansion interface ... 13
3.3 Input and tap coupler units ... 15
3.4 Detection unit ... 16
3.5 Matrix-multiply Cores ... 17
3.6 Micro-transfer printed semiconductor optical amplifiers ... 27
3.7 Dynamic calibration ... 28
3.8 Phase stabilization ... 29
Supplementary Note 4: Linear mode configuration ... 31
4.1 Experimental Configuration ... 31
4.2 Spectral information Acquisition ... 32
4.3 Spectral data analysis ... 33
4.4 Representative Input–Output Samples ... 36
Supplementary Note 5: Nonlinear mode configuration ... 42
5.1 Experimental Configuration ... 42
5.2 Driving model ... 43
5.3 Representative Input–Output Samples ... 45

Supplementary Note 6: Performance evaluation and scalability 48
6.1 Energy Efficiency 48
6.2 Recurrent depth 52
6.3 Normalized roofline model 54
6.4 Operation-matched raw single-pass compute delay 56
Supplementary Note 7: Three Forms of Temporal Plasticity 57
7.1 Predefined temporal plasticity 58
7.2 Analytical Temporal Plasticity 58
7.3 Data-driven Temporal Plasticity 59
7.4 Hardware Perspective 61
Reference 62

Supplementary Note 1: Linear-mode computing

1.1 The Richardson Method

The goal of matrix inversion is to solve the equation for $\boldsymbol{X}$:

$$\boldsymbol{YX} = \boldsymbol{I} \quad (\text{S1}-1)$$

where $\boldsymbol{Y} \in \mathbb{R}^{N\times N}$ and $\boldsymbol{I}$ is the unit matrix. Rather than computing $\boldsymbol{Y^{-1}}$ directly—which is often computationally prohibitive—Richardson iteration offers an efficient alternative that relies solely on matrix–vector products. The iterative strategy considers $\boldsymbol{X}_k$ as the current approximation of $\boldsymbol{Y^{-1}}$. We define the residual:

$$\boldsymbol{R}_k = \boldsymbol{I} - \boldsymbol{YX}_k \quad (\text{S1}-2)$$

which quantifies the deviation of $\boldsymbol{X}_k$ from the true inverse. To refine the approximation, we update $\boldsymbol{X}_k$ as:

$$\boldsymbol{X}_{k+1} = \boldsymbol{X}_k + \alpha\boldsymbol{R}_k = (\boldsymbol{I} - \alpha\boldsymbol{Y})\boldsymbol{X}_k + \alpha\boldsymbol{I} \ \ (k = 0,1,2,\dots) \quad (\text{S1}-3)$$

where $\boldsymbol{\alpha}$ is the parameter to control the convergence rate.

To enable efficient implementation on our TPPP, we adopt an equivalent formulation with the initialization $\boldsymbol{X}_0 = \alpha\boldsymbol{I}$:

$$\boldsymbol{X}_k = \sum_{\boldsymbol{i}=\boldsymbol{0}}^{\boldsymbol{k}} \left[(\boldsymbol{I} - \alpha\boldsymbol{Y})^i\right] \cdot \alpha\boldsymbol{I} \ \ (k = 0,1,2,\dots) \quad (\text{S1}-4)$$

where $\boldsymbol{I} - \alpha\boldsymbol{Y}$ is implemented in the MAC unit. The k -th term in the cumulative summation corresponds to the signal output produced at iteration k. The equivalence can be proven using mathematical induction as follows:

Base Case ($\boldsymbol{k} = \boldsymbol{0}$):

$$\boldsymbol{X}_0 = \sum_{\boldsymbol{i}=\boldsymbol{0}}^{\boldsymbol{0}} [(\boldsymbol{I} - \alpha\boldsymbol{Y})^0] \cdot \alpha\boldsymbol{I} = \alpha\boldsymbol{I} \quad (\text{S1}-5)$$

which agrees with the initialization. Thus, the base case holds.

Inductive Step:

Assume the formula holds for $k = n$:

$$\boldsymbol{X}_n = \sum_{\mathbf{q}=\mathbf{0}}^{\boldsymbol{n}} [(\boldsymbol{I} - \alpha\boldsymbol{Y})^q] \cdot \alpha\boldsymbol{I} \quad (\text{S1}-6)$$

Then, using the Richardson recurrence relation with substituting (S1-6):

$$\boldsymbol{X}_{n+1} = (\boldsymbol{I} - \alpha\boldsymbol{Y})\boldsymbol{X}_n + \alpha\boldsymbol{I} = (\boldsymbol{I} - \alpha\boldsymbol{Y})\sum_{\boldsymbol{i=0}}^{\boldsymbol{n}}\left[(\boldsymbol{I} - \alpha\boldsymbol{Y})^i\right]\cdot\alpha\boldsymbol{I} + \alpha\boldsymbol{I}$$

$$= \sum_{\boldsymbol{q=1}}^{\boldsymbol{n+1}}[(\boldsymbol{I} - \alpha\boldsymbol{Y})^q]\cdot\alpha\boldsymbol{I} + \boldsymbol{\alpha I} = \sum_{\boldsymbol{q=0}}^{\boldsymbol{n+1}}[(\boldsymbol{I} - \alpha\boldsymbol{Y})^q]\cdot\alpha\boldsymbol{I} \qquad (\mathrm{S1}-7)$$

Therefore, the formula holds for $k = n+1$. Thus, by mathematical induction, the closed-form expression (S1-4) holds for all $k \geq 0$.

1.2 Error Propagation, Convergence and Accuracy

Let the true inverse of $\boldsymbol{Y}$ be $\boldsymbol{X}^* = \boldsymbol{Y}^{-\boldsymbol{1}}$, At iteration k, the error is defined as:

$$\boldsymbol{E}_k = \boldsymbol{X}_k - \boldsymbol{X}^* \qquad (\mathrm{S1}-8)$$

Using the Richardson update and substituting $\boldsymbol{YX}^* = \boldsymbol{I}$:

$$\boldsymbol{E}_{k+1} = \boldsymbol{X}_{k+1} - \boldsymbol{X}^* = \boldsymbol{X}_k + \alpha(\boldsymbol{I} - \boldsymbol{Y}\boldsymbol{X}_k) - \boldsymbol{X}^* = (\boldsymbol{I} - \alpha\boldsymbol{Y})\boldsymbol{E}_k \qquad (\mathrm{S1}-9)$$

This linear recurrence shows that the error evolves through repeated application of the operator $(\boldsymbol{I} - \alpha\boldsymbol{Y})$. Convergence is guaranteed if the spectral radius satisfies:

$$\rho(\boldsymbol{I} - \alpha\boldsymbol{Y}) < 1 \qquad (\mathrm{S1}-10)$$

In many practical applications, including ridge regression, wireless communications, signal processing, and machine learning, the matrix $\boldsymbol{Y}$ is typically assumed to be symmetric positive definite (SPD). Under this assumption, the optimal α that yields the fastest convergence in Richardson iteration is given by:

$$\alpha = \frac{2}{\lambda_{\max} + \lambda_{\min}} \qquad (\mathrm{S1}-11)$$

where $\lambda_{\max}$ and $\lambda_{\min}$ are the maximum and minimum eigenvalues of $\boldsymbol{Y}$, respectively. The corresponding minimum number of iterations N required to achieve a convergence threshold ε is:

$$N = \frac{\ln\left(\frac{1}{\varepsilon}\right)}{\ln\left(\frac{\lambda_{\max} + \lambda_{\min}}{\lambda_{\max} - \lambda_{\min}}\right)} \qquad (\mathrm{S1}-12)$$

In practical implementations, each iteration of the algorithm is affected by noise arising from intrinsic physical mechanisms in photonic integrated circuits, including amplified spontaneous emission (ASE), thermal fluctuations, shot noise, and phase noise. Denoting the noise introduced at iteration k by N_k, the recurrence relation becomes:

$$\boldsymbol{X}_{k+1} = (\boldsymbol{I} - \alpha\boldsymbol{Y})\boldsymbol{X}_k + \alpha\boldsymbol{I} + \boldsymbol{N}_k, \quad (k = 0,1,2,\dots) \qquad (\mathrm{S1}-13)$$

With the initial condition $\boldsymbol{X}_0 = \alpha\boldsymbol{I}$, the closed-form expression for X_k is:

$$\boldsymbol{X}_k = \alpha\sum_{i=0}^{k}(\boldsymbol{I} - \alpha\boldsymbol{Y})^i + \sum_{j=0}^{k-1}(\boldsymbol{I} - \alpha\boldsymbol{Y})^{k-1-j}\boldsymbol{N}_j \; (k = 0,1,2,\dots) \qquad (\mathrm{S1}-14)$$

where the first summation term is the ideal, noise-free contribution, while the second summation term gathers the accumulated noise. Equations S1–14 establish a rigorous benchmark for evaluating experimental measurements against theoretical predictions, whilst providing an analytical framework for implementing active correction term $\boldsymbol{c_k}$ (equation 2, main text).

1.3 Power Splitting and Gain Compensation in Iterative Operation

In iterative optical systems, signal loss arises from intrinsic waveguide attenuation, fabrication imperfections, and coupling inefficiencies. Additionally, tap couplers extract a small fraction of optical power for real-time monitoring introduce further loss. To compensate for this accumulated attenuation, micro-transfer-printed semiconductor optical amplifiers (μTP SOAs) and external SOAs are integrated into the loop, providing the necessary gain to restore signal strength and maintain stable, efficient operation over multiple iterations. In numerical mode, considering all relevant losses, the *k*-th optical power allocated for monitoring, S_k , and the power transmitted to the next iteration, T_k , are given by:

$$\boldsymbol{S}_k = b \cdot a \cdot \boldsymbol{X}_k \tag{S1 − 15}$$

$$\boldsymbol{T}_k = c \cdot a \cdot \boldsymbol{X}_k \tag{S1 − 16}$$

Here, *b* and *c* denote the fractions of optical power used for monitoring and iteration, respectively, and *a* is the gain parameter. Substituting (S1-15) and (S1-16) into recurrence relation (S-13), we have:

$$\boldsymbol{S}_k = b \cdot a \cdot (\boldsymbol{A}^k \boldsymbol{X}_0 + \alpha \sum_{i=0}^{k-1} \boldsymbol{A}^i + \sum_{i=0}^{k-1} \boldsymbol{A}^{k-1-i} \boldsymbol{N}_i)(k = 0,1,2, \dots) \tag{S1 − 17}$$

$$\boldsymbol{T}_k = c \cdot a \cdot (\boldsymbol{A}^k \boldsymbol{X}_0 + \alpha \sum_{i=0}^{k-1} \boldsymbol{A}^i + \sum_{i=0}^{k-1} \boldsymbol{A}^{k-1-i} \boldsymbol{N}_i)(k = 0,1,2, \dots) \tag{S1 − 18}$$

where $\boldsymbol{A} = c \cdot a \cdot (\boldsymbol{I} - \alpha \boldsymbol{Y})$. To ensure numerical accuracy and iterative stability, the system should satisfy the lossless condition: $b \cdot a = c \cdot a = 1$.

1.4 Computational Efficiency Analysis

Our TPPP demonstrates substantial advantages over existing hardware architectures for matrix inversion computations. We benchmark our system against two dominant computational paradigms. The first category encompasses CPUs and streaming-class GPUs, which require repeated data fetching from HBM/DRAM at each iteration, incurring significant memory bandwidth bottlenecks. The second category includes TPUs, cached-class GPUs, and photonic single-pass processor (PSP), which maintain matrix $(\boldsymbol{I} - \alpha \boldsymbol{Y})$ stationary on-chip throughout iterations, reducing memory overhead.

Our TPPP architecture not only maintains matrix $(\boldsymbol{I} - \alpha\boldsymbol{Y})$ stationary on-chip but also enables systematic reuse of intermediate computational products across iterations. Let N be the matrix size and P iteration times. Table S1-1 presents benchmarks demonstrating these performance advantages.

TABLE S1-1 Computational Characteristics of Matrix Inversion Across Processor Architectures

	CPUs, streaming-class GPUs	TPUs, cached-class GPUs	TPPP
Input data size	$2N^2 \cdot P + N \cdot P$	$N^2 \cdot P + N \cdot P$	$N + N \cdot P$
Output data size	$N^2 \cdot P$	$N^2 \cdot P$	N^2
Memory access counts	$3N^2 \cdot P + N \cdot P$	$N^2 \cdot (2P + 1) + N \cdot P$	$2N^2 + N + N \cdot P$
Number of operations	$N^3 \cdot P$	$N^3 \cdot P$	$N^3 \cdot P$
Arithmetic intensity	$\frac{N^2}{3N + 1}$	$\frac{N^2 P}{(2N + 1)P + N}$	$\frac{N^2 P}{2N + P + 1}$

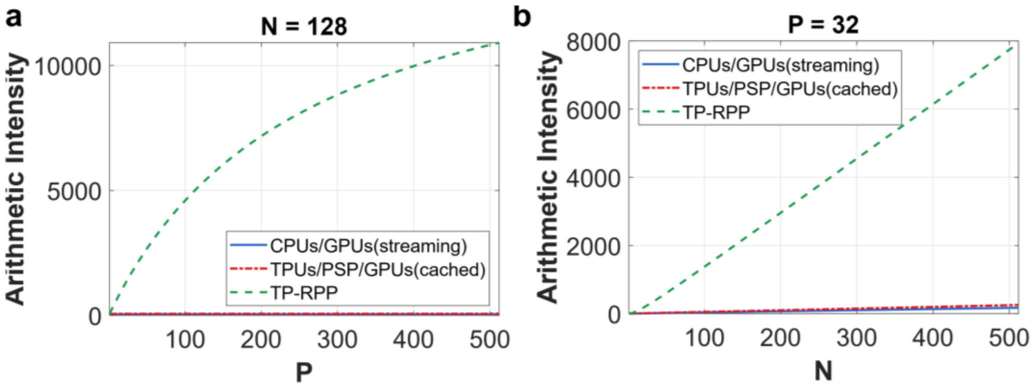

Fig. S1-1| Comparison of arithmetic intensity for matrix inversion across processor architectures.

a, Arithmetic intensity for three processor architectures with a fixed matrix size (N = 128) and an increasing number of iterations (P). b, Arithmetic intensity for three processor architectures with a fixed number of iterations (P = 32) and an increasing matrix size (N).

Figure S1-1 Compare arithmetic intensity (AI) for three architectures. With N=128 and rising iterations P, TPPP (green) scales with P, while streaming CPUs/GPUs and cached TPUs/GPUs stay near-constant (only tens), showing the benefit of reusing intermediates. At fixed P=32 and growing N, all lines rise ~linearly in N, but TPPP's slope ≈P/2 far exceeds cached and streaming, so its advantage amplifies with larger matrices or more iterations.

Supplementary Note 2: Nonlinear-mode computing

2.1 Time adaptive RNN

For layer/time step $k = 1, \dots, N$

$$a_k = \sigma_k(\boldsymbol{W}_{hh}(k)a_{k-1} + \boldsymbol{W}_{ih}(k)v_k + b_k), \qquad a_0 = \text{ initial state} \qquad (\text{S2}-1)$$

here a_k denote the hidden states after layer k with a_0 as the initial state. $\boldsymbol{W}_{hh}$ is the hidden-to-hidden state weight matrix, and $\boldsymbol{W}_{ih}$ is the coupling weight matrix for the layer-specific injected input. v_k is the external input unique to layer k. σ_k is the nonlinear activation for layer k. In this work, the activation is modelled as a scaled hyperbolic tangent function:

$$\sigma_k(x) = \tanh(\gamma x), \qquad (\text{S2}-2)$$

where γ controls the effective nonlinear slope. The tanh-like transfer function is implemented through photodetection-based electronic feedback, in which the detected optical power is used to dynamically adjust the EDFA pump current or SOA bias current. This feedback-controlled gain modulation shapes the effective gain transfer function in real time, thereby realizing the desired nonlinear activation. For the input-to-hidden ($\boldsymbol{W}_{ih}$) and hidden-to-hidden weights ($\boldsymbol{W}_{hh}$), we factorize both via SVD with fixed singular vectors and varying singular values:

$$\boldsymbol{W}_{\text{ih}} = \boldsymbol{U}_{\text{ih}} \times \text{diag}(\boldsymbol{S}_{\text{ih}}) \times \boldsymbol{V}_{\text{ih}}{}^{T} \qquad (\text{S2}-3)$$

$$\boldsymbol{W}_{\text{hh}} = \boldsymbol{U}_{\text{hh}} \times \text{diag}(\boldsymbol{S}_{\text{hh}}) \times \boldsymbol{V}_{\text{hh}}{}^{T}$$

The singular values evolve as:

$$\boldsymbol{S}_{\text{ih}}(k) = \boldsymbol{S}_{\text{ih}} + \lambda \times \Delta\boldsymbol{S}_{\text{ih}}(k) \qquad (\text{S2}-4)$$

$$\boldsymbol{S}_{\text{hh}}(k) = \boldsymbol{S}_{\text{hh}} + \lambda \times \Delta\boldsymbol{S}_{\text{hh}}(k)$$

where λ scales the time-dependent singular value modulations $\Delta\boldsymbol{S}_{\text{ih}}(k)$ and $\Delta\boldsymbol{S}_{\text{hh}}(k)$.

The step output can be produced from the hidden state via a linear or nonlinear head:

$$y_k = \boldsymbol{W}_{\text{ho}}a_k + b_{\text{o}} \text{ or } y_k = \sigma_{\text{o}}(\boldsymbol{W}_{\text{ho}}a_k + b_{\text{o}}) \qquad (\text{S2}-5)$$

2.2 Training Strategy

The training of TA-RNN follows a two-stage protocol. Stage 1 establishes baseline performance through standard RNN training using Adam optimisation. Stage 2 fine-tunes only the temporal modulation parameters $\Delta\boldsymbol{S}$, enabling specialised adaptation without catastrophic forgetting. The training process is detailed below:

1. **Input normalization.**

First, we compute the training split mean and standard deviation, then z-score normalize each input vector v_k. This step prevents gradient explosion and ensures that all input features contribute equally to the learning process. Note that targets need not be normalized since we want to preserve their original scale for interpretation. We choose the initial hidden state $a_0 = 0$.

2. Forward pass (per sequence; loop k = 1 → N)

The forward pass computes the network's predictions by sequentially processing each input in the sequence.

For stage 1, the pre-activation, state update and output can be expressed as:

$$z_k = \boldsymbol{W}_{hh} a_{k-1} + \boldsymbol{W}_{ih} v_k + b_k \qquad (\mathrm{S2}-6)$$

Similarly, for stage 2, we have:

$$z_k = \boldsymbol{U}_{\mathrm{hh}}\left(\boldsymbol{S}_{\mathrm{hh}}(k) \odot {\boldsymbol{V}_{\mathrm{hh}}}^T a_{k-1}\right) + \boldsymbol{U}_{\mathrm{ih}}\left(\boldsymbol{S}_{\mathrm{ih}}(k) \odot {\boldsymbol{V}_{\mathrm{ih}}}^T v_k\right) + b_k \qquad (\mathrm{S2}-7)$$

They share the same nonlinear activation and output:

$$a_k = \sigma_k(z_k) \qquad (\mathrm{S2}-8)$$

$$y_k = \boldsymbol{W}_{\mathrm{ho}} a_k + b_{\mathrm{o}} \qquad (\mathrm{S2}-9)$$

In the forward pass process, z_k, a_k, and the projections:

$$x_k^{\mathrm{proj}} = {\boldsymbol{V}_{\mathrm{ih}}}^T v_k \;\; h_k^{\mathrm{proj}} = {\boldsymbol{V}_{\mathrm{hh}}}^T a_{k-1} \qquad (\mathrm{S2}-10)$$

are stored for back propagation.

3. Loss calculation

After completing the forward pass for all sequences in our batch, we need to quantify how well our network performed by comparing predictions to targets. For M sequences with targets y_k^*, the loss can be calculated:

$$\mathcal{L} = \frac{1}{MN} \sum_{m=1}^{M} \sum_{k=1}^{N} \left\| y_k^{(m)} - y_k^{*(m)} \right\|_2^2 \qquad (\mathrm{S2}-11)$$

4. Back propagation

Now we perform backpropagation through time (BPTT) to compute gradients. We work backward from the final timestep to efficiently propagate error signals through the temporal dependencies.

Step A: Head Gradient (Linear Head)

We begin by computing gradients with respect to the output layer, and accumulate gradients for the output layer parameter:

$$\frac{\partial \mathcal{L}}{\partial a_k} \mathrel{+}= {\boldsymbol{W}_{\mathrm{ho}}}^T \frac{\partial \mathcal{L}}{\partial y_k}, \qquad \frac{\partial \mathcal{L}}{\partial \boldsymbol{W}_{\mathrm{ho}}} = \sum_{m,k} \frac{\partial \mathcal{L}}{\partial y_k}^{(m)} a_k^{T(m)}, \qquad \frac{\partial \mathcal{L}}{\partial b_{\mathrm{o}}} = \sum_{m,k} \frac{\partial \mathcal{L}}{\partial y_k}^{(m)} \qquad (\mathrm{S2}-12)$$

Step B: Nonlinearity Derivative

Next, we propagate the gradient through the nonlinear activation function. For the hyperbolic tangent activation, the derivative and the gradient with respect to the pre-activation are:

$$\sigma(z_k) = \tanh(\gamma z_k), \sigma'(z_k) = \gamma \mathrm{sech}^2(\gamma z_k) = \gamma[1 - \tanh^2(\gamma z_k)],$$
$$\frac{\partial \mathcal{L}}{\partial z_k} = \frac{\partial \mathcal{L}}{\partial a_k} \odot \sigma'(z_k) = \frac{\partial \mathcal{L}}{\partial a_k} \odot \gamma[1 - a_k \odot a_k]. \qquad \text{(S2-13)}$$

Step C: Transform Gradients to SVD Coordinates (Stage 2)

In the SVD formulation, we need to transform gradients from the original matrix space into the factorized coordinate system. This involves projecting the pre-activation gradients onto the left singular vectors:

$$g_{k,ih}^{(r)} = \boldsymbol{U}_{\mathrm{ih}}{}^T \frac{\partial \mathcal{L}}{\partial z_k}, \qquad g_{k,hh}^{(r)} = \boldsymbol{U}_{\mathrm{hh}}{}^T \frac{\partial \mathcal{L}}{\partial z_k} \qquad \text{(S2 − 14)}$$

These projections allow us to compute gradients efficiently in the reduced-rank space.

Step D: Gradients for Time-Varying Singular Values (Stage 2 Trainable)

Since Stage 2 optimizes the time-varying singular value adjustments, we compute gradients for these parameters:

$$\frac{\partial \mathcal{L}}{\partial \boldsymbol{S}_{\mathrm{ih}}(k)} = \sum_{m=1}^{M} g_{k,ih}^{(r)(m)} \odot x_k^{\mathrm{proj(m)}}, \ \frac{\partial \mathcal{L}}{\partial \boldsymbol{\Delta S}_{\mathrm{ih}}(k)} = \lambda \frac{\partial \mathcal{L}}{\partial \boldsymbol{S}_{\mathrm{ih}}(k)} \qquad \text{(S2 − 15)}$$

$$\frac{\partial \mathcal{L}}{\partial \boldsymbol{S}_{\mathrm{hh}}(k)} = \sum_{m=1}^{M} g_{k,hh}^{(r)(m)} \odot h_k^{\mathrm{proj(m)}}, \qquad \frac{\partial \mathcal{L}}{\partial \boldsymbol{\Delta S}_{\mathrm{hh}}(k)} = \lambda \frac{\partial \mathcal{L}}{\partial \boldsymbol{S}_{\mathrm{hh}}(k)} \qquad \text{(S2 − 16)}$$

Step E: Bias Gradients

The bias at step k:

$$\frac{\partial \mathcal{L}}{\partial b_k} = \sum_{m=1}^{M} \frac{\partial \mathcal{L}}{\partial z_k}^{(m)} \qquad \text{(S2 − 17)}$$

Step F: Backpropagation to Inputs and Previous Hidden States

Finally, we compute gradients with respect to the inputs and previous hidden states:

$$\frac{\partial \mathcal{L}}{\partial v_k} = \boldsymbol{V}_{\mathrm{ih}} \left(g_{k,ih}^{(r)} \odot \boldsymbol{S}_{\mathrm{ih}}(k) \right), \qquad \left. \frac{\partial \mathcal{L}}{\partial a_{k-1}} \right|_{z_k} = \boldsymbol{V}_{\mathrm{hh}} \left(g_{k,hh}^{(r)} \odot \boldsymbol{S}_{\mathrm{hh}}(k) \right) \qquad \text{(S2 − 18)}$$

We accumulate $\frac{\partial \mathcal{L}}{\partial a_{k-1}}$ into the running gradient for a_{k-1}.

For Stage 1 training, we replace Steps C–F with standard dense matrix gradient computations:

$$\frac{\partial \mathcal{L}}{\partial \boldsymbol{W}_{\mathrm{ih}}} = \sum_{m,k} \frac{\partial \mathcal{L}}{\partial z_k}^{(m)} v_k{}^{T(m)}, \qquad \frac{\partial \mathcal{L}}{\partial \boldsymbol{W}_{\mathrm{hh}}} = \sum_{m,k} \frac{\partial \mathcal{L}}{\partial z_k}^{(m)} a_{k-1}{}^{T(m)} \qquad \text{(S2 − 19)}$$

$$\frac{\partial \mathcal{L}}{\partial v_k} = \boldsymbol{W}_{\mathrm{ih}}{}^T \frac{\partial \mathcal{L}}{\partial z_k}, \qquad \left. \frac{\partial \mathcal{L}}{\partial a_{k-1}} \right|_{z_k} = \boldsymbol{W}_{\mathrm{hh}}{}^T \frac{\partial \mathcal{L}}{\partial z_k} \qquad \text{(S2 − 20)}$$

4. Parameter update

With all gradients computed, we can now update the network parameters using our chosen optimization algorithm Adam. The parameter update strategy depends on the training stage:

Stage 1: We update all parameters including $\boldsymbol{W}_{\mathrm{hh}}$, $\boldsymbol{W}_{\mathrm{ih}}$, $\boldsymbol{W}_{\mathrm{ho}}$, b_{o} and b_{k}. This allows the network to learn the optimal full-rank representation.

Stage 2: We freeze the SVD components $\boldsymbol{U}_{\mathrm{hh}}$, $\boldsymbol{U}_{\mathrm{ih}}$, $\boldsymbol{S}_{\mathrm{hh}}$, $\boldsymbol{S}_{\mathrm{ih}}$, ${\boldsymbol{V}_{\mathrm{hh}}}^T$, ${\boldsymbol{V}_{\mathrm{ih}}}^T$as well as biases b_{o}, b_{k}, $\boldsymbol{W}_{\mathrm{ho}}$, and update only the time-adaptive adjustments $\boldsymbol{\Delta S}_{\mathrm{hh}}$, $\boldsymbol{\Delta S}_{\mathrm{ih}}$.

5.Training Loop Continuation

We repeat the forward pass, loss computation, backward pass, and parameter update cycle over multiple epochs until convergence. Importantly, in Stage 2, we begin from the exact Stage 1 solution after performing SVD decomposition of the learned weight matrices. This initialization ensures that Stage 2 training starts from a good baseline and focuses on learning the time-adaptive components $\boldsymbol{\Delta S}_{\mathrm{hh}}(k)$ and $\boldsymbol{\Delta S}_{\mathrm{ih}}(k)$ that can capture temporal variations in the data.

Supplementary Note 3: Functional parts characterization

3.1 Fiber-to-chip couplers

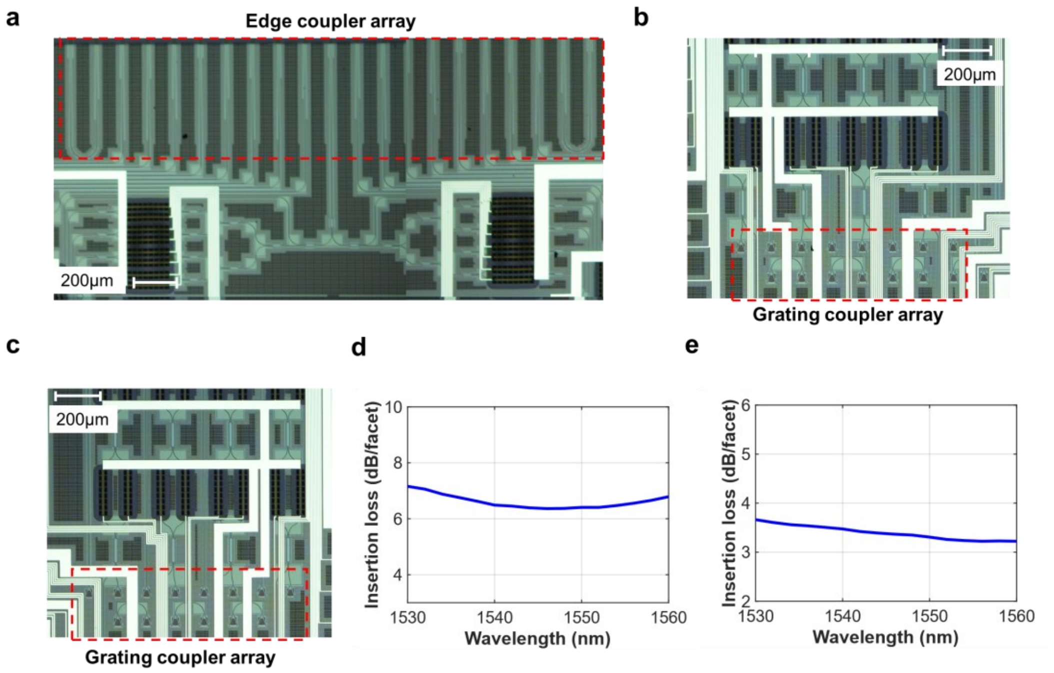

Fig. S3-1 | Performance of edge couplers and grating couplers.

a, Photographs of the edge coupler array**. b,** Photographs of the grating coupler array in the expansion interface. **c,** Photographs of the grating coupler array in the detection unit. Measured insertion losses of **d**, grating coupler and **e** edge coupler.

Edge couplers and grating couplers establish critical optical interfaces between external fibres and the on-chip photonic network. Here, edge couplers facilitate low-loss signal injection at the input unit and direct output to photodetectors, whilst grating couplers enable versatile integration with external optical components for system expansion.

Figure S3-1a-c presents fabricated edge coupler array and grating coupler arrays. Edge couplers achieve insertion losses of approximately 3.4 dB per facet at 1550 nm wavelength (Fig. S3-1e), making them ideal for high-fidelity signal transmission in the primary optical path. Grating couplers exhibit higher insertion losses of approximately 6.5 dB per facet (Fig. S3-1d), yet their out-of-plane coupling capability and fabrication simplicity justify their deployment for auxiliary connections and system modularity.

3.2 Expansion interface

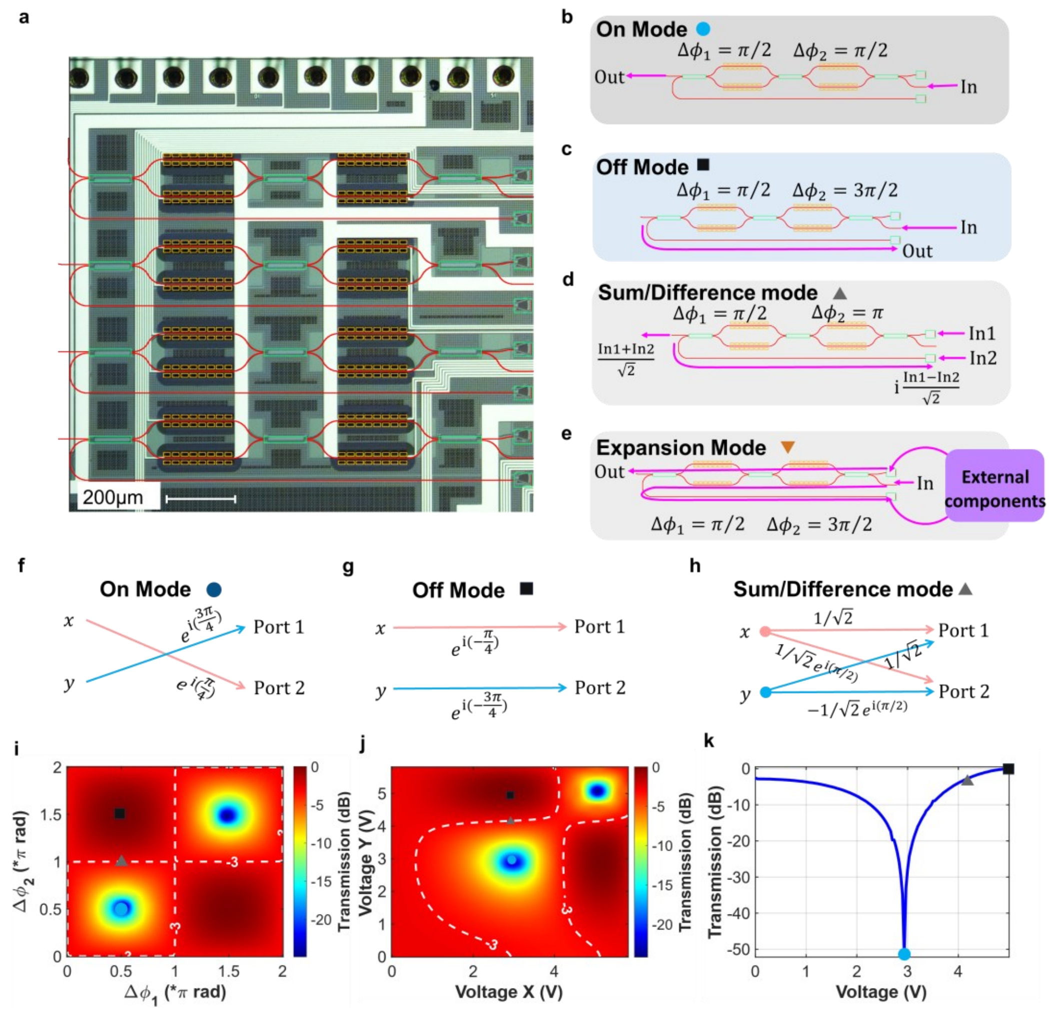

Fig. S3-2 | Expansion interface: design, microscopy, and performance.

a, Illustration and optical micrograph of the expansion interface with key pathways annotated. Schematic illustrations of light paths and phase difference configuration under different operational modes: **b**, On mode. **c**, Off mode. **d**, Sum/Difference mode. **e**, Expansion mode. Light path and corresponding phase shift for **f**. On mode, **g**, Off mode and **h**, Sum/Difference mode. **i**, Simulated two-dimensional scan of the optical transmission as a function of the phase differences in the two serially connected phase-shifting arms. **j**, Measured two-dimensional transmission mapping from voltage scanning of the two serially connected shifters. **k**, Measured transmission curve when one phase-shifting arm is fixed at a π/2 phase difference, while the voltage on the other arm is scanned.

The expansion interface enables seamless transitions between on-chip processing and external optical operations. Each channel incorporates three cascaded 2×2 multimode interference (MMI) couplers interleaved with two pairs of programmable phase shifters, as illustrated in Fig. S3-2a, creating a versatile optical routing matrix.

The proposed expansion interface four distinct operational modes through precise phase control. In the On mode (Fig. S3-2b), $\pi/2$ phase differences in both shifting arms route signals directly through the expansion interface, maintaining on-chip propagation. The Off mode (Fig. S3-2c) employs asymmetric phase configuration—$\pi/2$ and $3\pi/2$ in the first and second arms respectively, redirecting on-chip signals to grating couplers for free-space emission. The Sum/Difference mode (Fig. S3-2d) demonstrates the interface's computational capabilities. With phase differences of $\pi/2$ and π, the system performs simultaneous addition and subtraction operations on dual inputs—one from on-chip and another via grating coupler. The sum emerges at the on-chip output whilst the difference exists through the secondary grating coupler, both scaled by a factor of $1/\sqrt{2}$. The Expansion mode (Fig. S3-2e) facilitates hybrid computing by routing on-chip signals through external optical elements. Signals exit via one grating coupler, interact with off-chip components, and return through the adjacent coupler—effectively extending the computational domain beyond silicon photonics.

Figure S3-2 f–h show the propagation paths and phase delays at the expansion interface, and its corresponding equivalent transfer matrix are given below:

On mode: $\begin{bmatrix} 0 & e^{\mathrm{i}(\pi/4)} \\ e^{\mathrm{i}(\pi/4)} & 0 \end{bmatrix}$

Off mode and expansion mode: $\begin{bmatrix} e^{-\mathrm{i}(\pi/4)} & 0 \\ 0 & e^{-\mathrm{i}(3\pi/4)} \end{bmatrix}$

$\frac{\textbf{Sum}}{\textbf{Difference}}$ **mode**: $\frac{1}{\sqrt{2}}\begin{bmatrix} 1 & 1 \\ e^{\mathrm{i}(\pi/2)} & -e^{\mathrm{i}(\pi/2)} \end{bmatrix}$

In On mode, light cross-couples: input x exits at Port 2 with a phase factor $e^{\mathrm{i}(\pi/2)}$, and input y exits at Port 1 with $e^{\mathrm{i}(3\pi/4)}$. In Off mode (g), light passes straight through, input x remains at Port 1 with $e^{-\mathrm{i}(\pi/4)}$, and input y remains at Port 2 with $e^{-\mathrm{i}(3\pi/4)}$. In Sum/Difference mode (h), Port 1 outputs the sum of the two inputs, while Port 2 outputs their difference and is delayed by an additional phase of $\pi/2$ relative to the sum port. Sum and difference of the inputs are both scaled by a factor of $1/\sqrt{2}$.

Two-dimensional transmission mapping reveals the relationship between phase configurations and optical transmission (Fig. S3-2 i, j). Simulated (Fig. S3-2 i) and experimentally measured (Fig. S3-2 j) mappings demonstrate remarkable agreement. Each operational mode corresponds to at least two equivalent phase configurations; we selected the low-power-consumption states (annotated points) to minimize thermal crosstalk and energy dissipation. The measured transmission curve (Fig. S3-2k), obtained by fixing one phase shifter at $\pi/2$ whilst scanning the other, confirms sharp transitions between modes. The >50 dB On-to-Off extinction ratio—achieved without active feedback—demonstrates its stability for maintaining signal integrity in cascaded photonic processors.

3.3 Input and tap coupler units

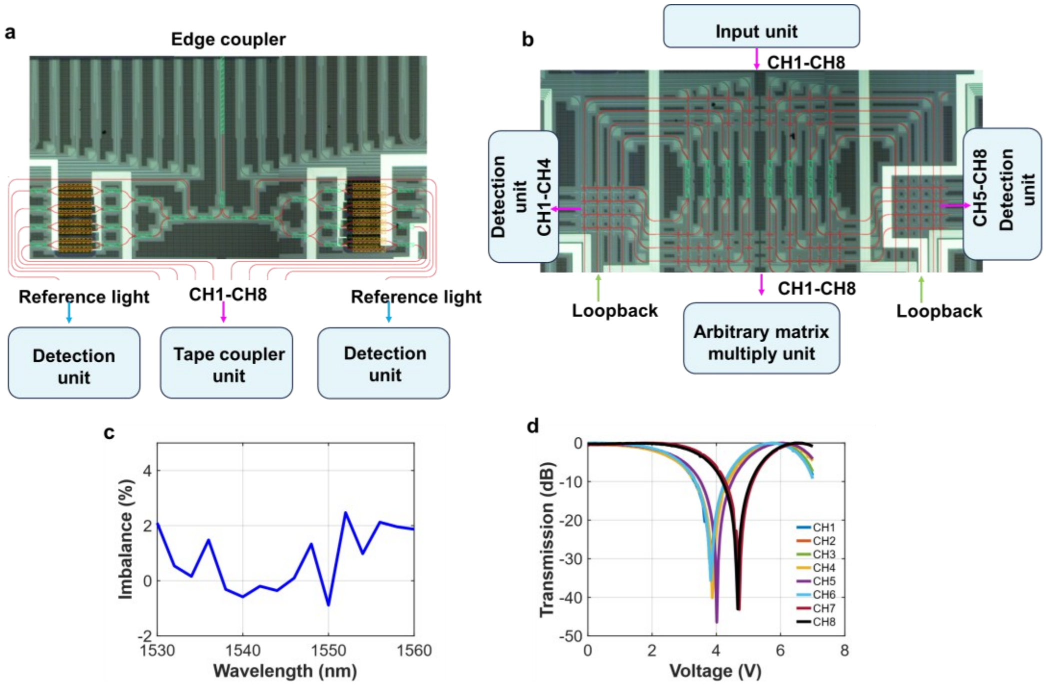

Fig. S3-3 | Input and tap coupler units: design, microscopy, and performance.

a, Illustration and optical micrograph of the input unit with key pathways annotated. **b**, Illustration and optical micrograph of the tap coupler unit, with key pathways annotated. **c**, Measured power imbalance of the directional coupler. **d**, Optical transmission for all eight channels of the input unit.

Figure S3-3 a-b present optical micrographs of the input and tap coupler units. The input unit (Fig. S3-3a) receives optical signals through edge couplers and implements a 1×10 power splitting network. Two output channels are dedicated as reference signals for coherent detection, while the remaining eight are distributed to parallel Mach-Zehnder interferometer (MZI) modulators for initial signal conditioning. The tap coupler unit (Fig. S3-3b) orchestrates bidirectional signal flow through eight directional couplers. It supports two operational modes: forward propagation from the input unit and iterative feedback via loopback pathways. Each directional coupler performs 50:50 power splitting—routing half the optical power to the detection unit for real-time monitoring whilst directing the remainder to the matrix-multiply unit for subsequent computational processing.

The directional couplers achieve power splitting ratios within 1% of the ideal 50:50 target at 1550 nm wavelength (Fig. S3-3c). Channel-to-channel transmission measurements across the input unit (Fig. S3-3d) reveal consistent modulation characteristics for channels 1–6. Channels 7–8 display elevated voltage requirements for equivalent modulation depths, attributable to fabrication variations.

3.4 Detection unit

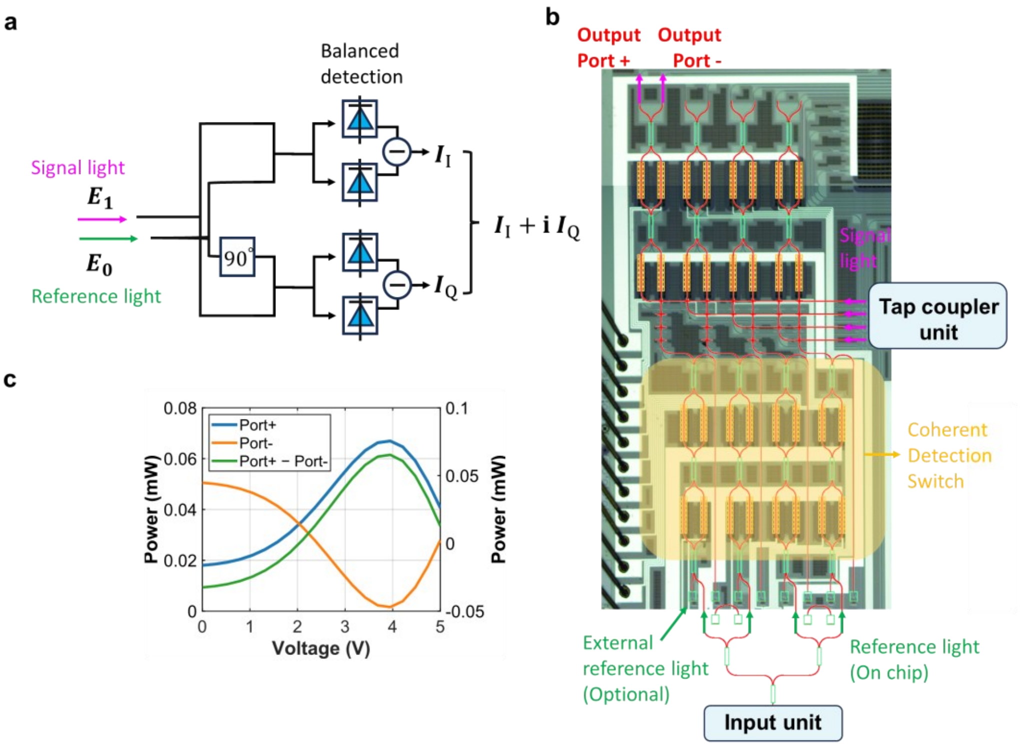

Fig. S3-4 | Detection units: design, microscopy, and performance.

a, Schematic of in-phase/quadrature (I/Q) homodyne coherent detection. **b**, Illustration and optical micrograph of the detection unit, with key pathways annotated. **c**, Measured coherent detection performance.

Standard intensity detection measures only optical power ($|E|^2$), discarding crucial phase information. This limitation severely constrains optical computing architectures to real-valued operations. Here, I/Q Homodyne Coherent Detection is utilized to extract both amplitude and phase from optical output signals, enabling complex-valued computation for potential advanced applications.

The principle of coherent detection is given in Fig. S3-4 a. The I/Q Homodyne Coherent Detection Unit mixes output signals $E_1(t)$ with a local reference $E_0(t)$ through an integrated Mach-Zehnder interferometer (MZI), where both fields share identical frequency and polarisation:

$$E_1(t) = A_1(t)\mathrm{e}^{\mathrm{i}(\omega_1 \mathrm{t}+\phi_1)} \qquad (\mathrm{S3}-1)$$

$$E_0(t) = A_0(t)\mathrm{e}^{\mathrm{i}(\omega_0 \mathrm{t}+\phi_0)} \qquad (\mathrm{S3}-2)$$

The MZI generates two output ports whose interference patterns encode the relative phase $\Delta\phi = \phi_1 - \phi_0$.Setting the internal MZI phase to $\theta = \pi/2$ yields output intensities:

$$I_1 = \frac{1}{2}|A_1|^2 + \frac{1}{2}|A_0|^2 + A_1 A_0 \cos(\Delta\phi) \qquad (\mathrm{S3}-3)$$

$$I_2 = \frac{1}{2}|A_1|^2 + \frac{1}{2}|A_0|^2 - A_1 A_0 \cos(\Delta\phi) \qquad (\mathrm{S3}-4)$$

Balanced detection ($I_1 - I_2$) extracts the in-phase component:

$$I_I = 2A_1A_0\cos(\Delta\phi) \qquad (S3-5)$$

To obtain the quadrature component, we introduce an additional $\pi/2$ phase shift to the reference arm. This generates outputs E_3 and E_4 , yielding:

$$I_3 = \frac{1}{2}|A_1|^2 + \frac{1}{2}|A_0|^2 + A_1A_0\sin(\Delta\phi) \qquad (S3-6)$$

$$I_4 = \frac{1}{2}|A_1|^2 + \frac{1}{2}|A_0|^2 - A_1A_0\sin(\Delta\phi) \qquad (S3-7)$$

The quadrature component ($I_3 - I_4$) is extracted:

$$I_Q = 2A_1A_0\sin(\Delta\phi) \qquad (S3-8)$$

I_Q, together with I_I, determines $\Delta\phi$, and $2A_0$ can be considered as a global scaling factor.

In this work, we only implement signed number calculation, thus full complex-valued encoding is unnecessary; a π-phase shift suffices to represent negative values (i.e., binary phase-shift keying, BPSK). Consequently, the in-phase component alone determines the sign:

$$I_I = 2A_1A_0\cos(\Delta\phi + \pi) = -2A_1A_0\cos(\Delta\phi) \qquad (S3-9)$$

Figure S3-4 b presents the physical implementation within the detection unit. Each channel comprises a 2×2 MZI incorporating both internal and external phase shifters. The internal shifter maintains a fixed π/2 phase difference between interferometer arms. Within the carefully calibrated interferometer, the signal light from the tap coupler unit mixes with the reference light from the input unit. Additionally, we route the reference path through an expansion interface before mixing, providing operational flexibility. This architecture supports three detection modes: coherent detection using the on-chip reference, intensity-only detection with the reference disabled, or enhanced coherent detection using an external reference source coupled through grating couplers. Such versatility enables dynamic switching between real and complex-valued processing within the same hardware platform. Experimental validation confirms robust coherent detection performance (Fig. S3-4 c). Balanced photodetectors monitor both output ports of the interferometer, capturing complementary power variations as the external phase shifter modulates the input's signal phase. The measured response demonstrates smooth transitions of the in-phase component I_I from negative to positive values with increasing voltage.

3.5 Matrix-multiply Cores

Calibration methodology

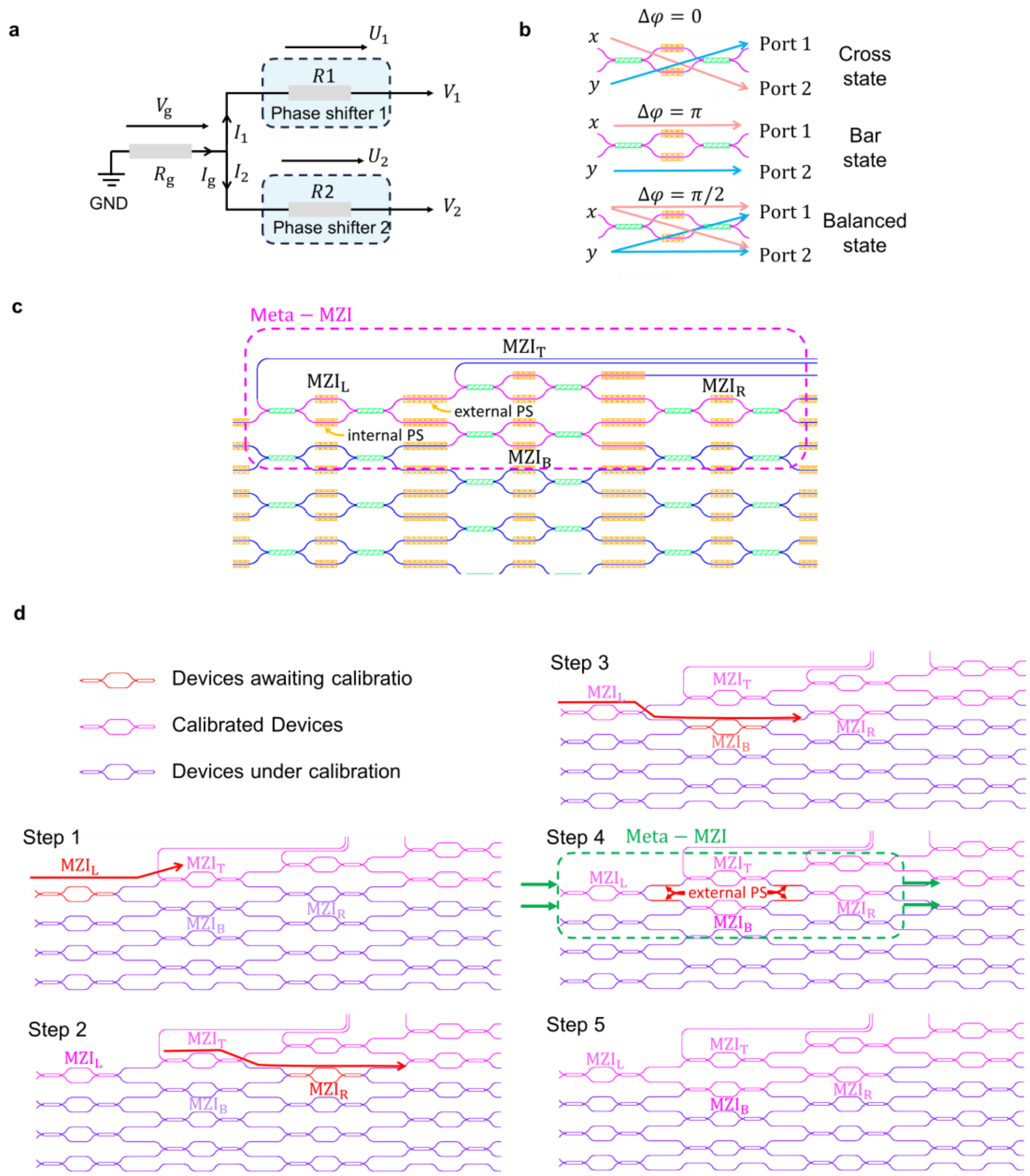

Fig. S3-5 | Characterization methodology for an U(2) unit cell.

a, Electrical model of the voltage-driven phase shifters, accounting for shared ground-path resistance. b. Three configurations of U(2) unit cell used for characterization **c**, Characterization of external and internal phase shifters within unitary matrix-multiply unit. **d**, Characterization of external and internal phase shifters within arbitrary matrix-multiply unit.

To ensure compatibility with our custom voltage-driven control electronics, we employed a voltage-based driving scheme for the thermo-optic phase shifters. Figure S3-5 a illustrates the electrical model, where two voltage-controlled phase shifters share a common ground path. Each phase shifter exhibits resistances $R1$ and $R2$, whilst the shared ground contributes resistance R_g. Although applied voltages $V1$

and $V2$ differ from the actual voltages $U1$ and $U2$ across the phase shifters due to ground coupling, Kirchhoff's law yields:

$$U1 = \frac{R1 \cdot \left[\left(R2 + R_g \right) \cdot V1 - R_g \cdot V2 \right]}{R1 \cdot R2 + R1 \cdot R_g + R2 \cdot R_g} \qquad (S3-15)$$

$$U2 = \frac{R2 \cdot \left[\left(R1 + R_g \right) \cdot V2 - R_g \cdot V1 \right]}{R1 \cdot R2 + R1 \cdot R_g + R2 \cdot R_g} \qquad (S3-16)$$

For our chip design, where $R1$, $R2 \gg R_g$, the approximations $U1 \approx V1$ and $U2 \approx V2$ remain valid, simplifying control requirements.

We calibrated the U(2) unit cells using interference fringe measurements. Each Mach-Zehnder interferometer (MZI) operates in three fundamental configurations (Fig. S3-5 b):

Cross state (φ = 0): Perfect mode exchange between ports, routing each input to the opposite output with unity transmission.

Balanced state (φ = π/2): Symmetric 50:50 beam splitting, equally distributing input power between outputs.

Bar state (φ = π): Direct routing without cross-coupling, each input mapped to its corresponding output.

Our matrix-multiply unit integrates two distinct cores. Core 1 implements a Reck-configuration MZI mesh for unitary matrix operations, whilst Core 2 implements a Clements-configuration mesh that performs arbitrary, non-expansive linear transformations using singular value decomposition (SVD). Waveguide interconnects between Core 1's bottom channel and Core 2's top channel enable optical signal exchange between the two cores, facilitating systematic calibration.

The Reck architecture's triangular topology enables sequential calibration from the apex downwards (Fig. S3-5 c). Our five-step procedure establishes initial phase control:

Step 1: Input light enters port x of the topmost MZI (MZI_T). Scanning the internal phase shifter voltage whilst monitoring port 1 yields MZI_T's transmission curve, completing its calibration.

Step 2: With MZI_T set to cross configuration, light enters MZI_L's port x. Monitoring MZI_T's port 1 whilst scanning MZI_L's internal phase voltage produces MZI_L's transmission curve.

Step 3: Maintaining MZI_T in cross configuration, light enters MZI_T's port x. Scanning MZI_R's internal phase voltage whilst monitoring MZI_R's port 1 calibrates this element.

Step 4: Both MZI_L and MZI_R configured to cross state, light enters MZI_L's port x. Monitoring MZI_R's port 1 whilst scanning MZI_B's voltage yields the bottom element's calibration.

Step 5: The four MZIs now form a meta-interferometer. Setting MZI_L and MZI_R to balanced configuration and MZI_T and MZI_B to bar configuration, we scan the external phase shifters (now internal to the meta-MZI) whilst monitoring MZI_R's port 1, completing the external phase shifters calibration.

This hierarchical approach extends throughout Core 1's Reck network, progressively forming meta-interferometers for U(2) unit cells calibration within Core 1.

Following Core 1's complete calibration, Core 2 leverages these established optical paths (Fig. S3-5 d). For boundary-region U(2) cells within Core 2, we select a calibrated Core 1 MZI as the reference element MZI_T. The calibration sequence (steps 2–5) proceeds identically, with monitoring signals routed through Core 1's calibrated pathways to the detector. This approach ensures consistent phase characterisation across both cores whilst exploiting the architectural coupling between Reck and Clements configurations.

Characterization of U(2) unit cell

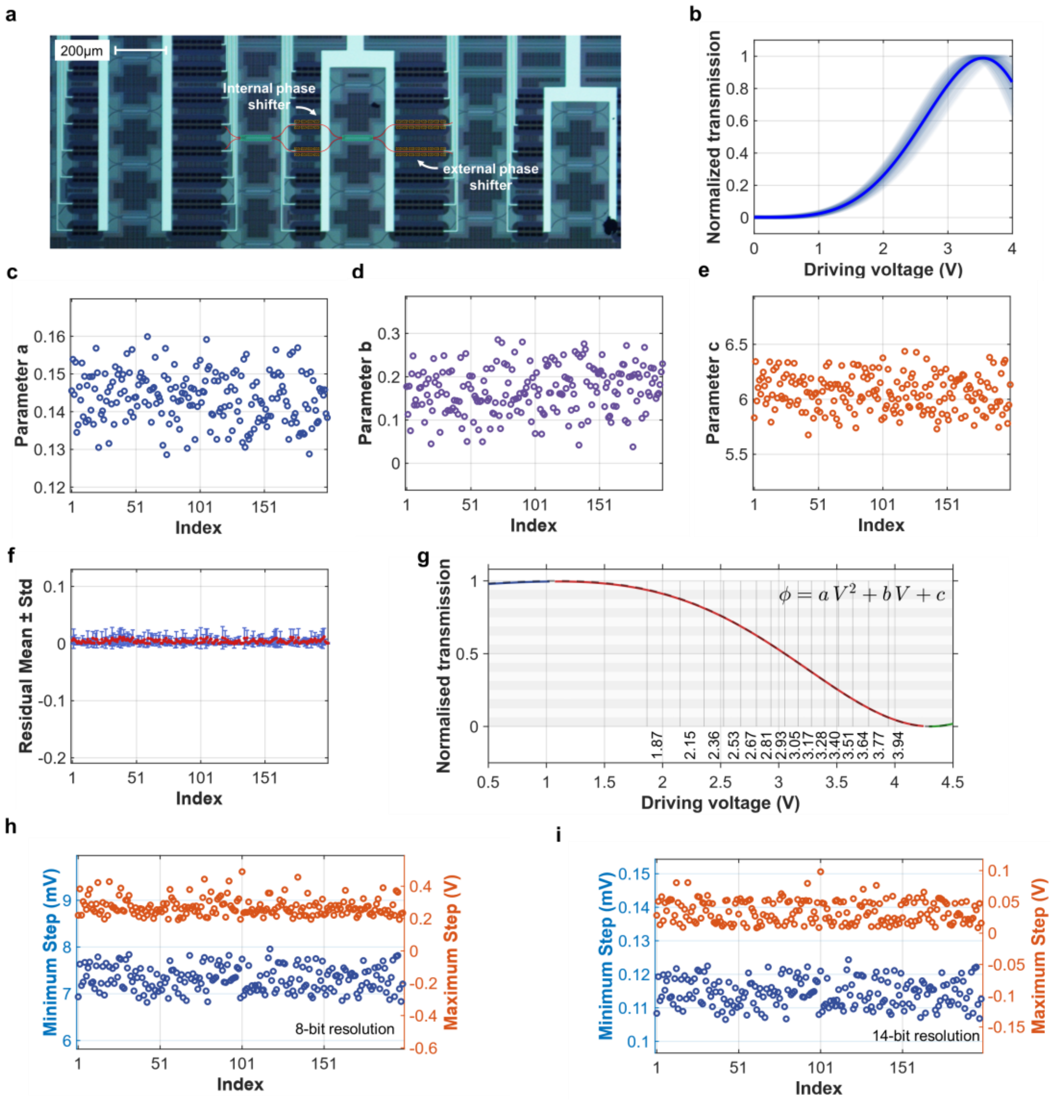

Fig. S3-6 | Characterization of U(2) unit cell.

a, Optical micrograph of the U(2) unit cell. **b**, Transmission characteristics of Mach–Zehnder interferometers (MZIs) measured during internal phase-shifter sweeps. The solid blue line represents the average transmission curves across 200 MZIs, with the shaded region indicating the full range of individual device responses. Extracted fitting parameters from transmission curves measured during external phase shifter sweeps: **c**, parameter a, **d**, parameter b, **e**. parameter c **f**. Residual analysis showing mean and standard deviation after curve fitting for external phase shifter sweeping, **g**, Illustration of digitalized MZI transmission curves under external phase shifter tuning. Required maximum and minimum voltage sweep step for the external phase shifter to achieve **h**, 8-bit and **i**, 14-bit resolution.

Photonic matrix multiplication relies on cascaded networks of U(2) unit cells (Fig. S3-6 a), making precise characterization of individual cells critical for computational accuracy. Each U(2) unit cell

comprises a 2×2 Mach-Zehnder interferometer (MZI) with integrated phase control. Internal phase shifters (100 μm) positioned between couplers control the differential phase θ between MZI arms, whilst external phase shifters (200 μm) at the output ports set the relative phase φ. The U(2) unit cell implements the unitary transformation:

$$U(2) = \begin{bmatrix} e^{\mathrm{i}\phi} \sin\left(\frac{\theta}{2}\right) & e^{\mathrm{i}\phi} \cos\left(\frac{\theta}{2}\right) \\ \cos\left(\frac{\theta}{2}\right) & -\sin\left(\frac{\theta}{2}\right) \end{bmatrix} \qquad (S3-17)$$

where both the sweep of θ and φ follow quadratic voltage dependencies: $aV^2 + bV + c$. Figure 2d and Fig. S3-6 b demonstrates the transmission characteristics across 200 MZIs during external and internal phase-shifter sweeps. The devices exhibit remarkable uniformity—the mean transmission curve (solid blue) closely tracks individual device responses (shaded region). Full amplitude modulation (0 to 1) of internal phase shifter requires approximately 3.5 V, corresponding to a power efficiency of 18.8 mW/π, a lower efficiency than external phase shifters.

To illustrate calibration requirements and control precision, we use the characterization of external phase shifters as a representative example. Figs S3-6 c–e presents the extracted fitting parameters of the external phase shifters, and the corresponding residual analysis (Fig. S3-6 f) validates our fitting approach: amongst 200 devices, residual standard deviations remain within 0.1, demonstrating excellent model accuracy. Figure S3-6 g illustrates the discretization scheme: the transmission range (0–1) divides uniformly into 2^n levels, with required voltages determined via inverse function mapping from fitted curves. For 8-bit amplitude resolution (Fig. S3-6 h), external phase shifters require only 10-bit digital-to-analogue converters (DACs), with maximum and minimum voltage steps comfortably within standard tolerances. However, achieving 14-bit resolution (Fig. S3-6 i) demands at least 16-bit DACs, as the minimum voltage step approaches noise floors. The internal phase shifters exhibit similar fitting curves but with a lower tunning efficiency, and the details are not repeated here.

Crosstalk Suppression

Whilst we have successfully established individual U(2) unit cell calibration, matrix operations require coordinated control of multiple cells operating simultaneously. This collective operation introduces crosstalk—a fundamental challenge that must be addressed for accurate photonic computing at scale. We develop a comprehensive crosstalk model to capture inter-cell coupling effects. For the i-th phase shifter, the actual phase shift ϕ_i incorporates contributions from all active elements:

$$\phi_i = c_i + \sum_j C_{ij} \cdot (a_j {V_j}^2 + b_j V_j) \qquad (S3-18)$$

Here, parameters $a_{i,}$ $b_{i,}$, and $c_{i,}$ derive from individual cell calibration (previous section), whilst C_{ij} represents the crosstalk matrix. Diagonal elements $C_{ii} \approx 1$ capture self-action, whereas off-diagonal elements $C_{ij} \ll 1$ quantify coupling between distinct phase shifters. The summation spans all channels, including j = i, with V_j denoting the voltage applied to channel j.

This general formulation presents a computational challenge: calibrating n phase shifters requires characterising n^2 coupling coefficients. For a 64×64 photonic processor containing thousands of phase shifters, full calibration becomes prohibitively complex.

We exploit the physical locality of crosstalk mechanisms to dramatically reduce calibration overhead. Both thermal and electrical crosstalk exhibit strong distance dependence—coupling strength decreases rapidly with separation between elements. This insight enables a nearest-neighbor approximation, where we retain only C_{ij} values for physically adjacent phase shifters, setting all other off-diagonal elements to zero. This sparse-matrix approach aligns naturally with our chip architecture. Phase shifters are arranged in regular arrays with well-defined neighbour relationships, making the nearest-neighbor model both physically motivated and computationally efficient. The calibration complexity reduces from $O(n^2)$ to $O(n)$, enabling practical characterization of large-scale photonic processors. Under the nearest-neighbor approximation, we have:

$$\phi_i = c_i + C_{ii}(a_i V_i^2 + b_i V_i) + \sum_{j \in \mathcal{N}(i)} C_{ij}\,(a_j V_j^2 + b_j V_j) \qquad (S3-19)$$

where $\mathcal{N}(i)$denotes the set of nearest-neighbour phase shifters around element i. The linear superposition principle enables direct experimental extraction of coupling coefficients through isolated perturbation measurements. To measure the crosstalk coefficient C_{ij} between neighbouring devices, we configure device i at its minimum transmission point—where phase-dependent intensity variations exhibit maximum sensitivity. We then systematically vary the voltage applied to each neighboring device j whilst monitoring the transmission through device i. A series of voltage sweeps generates the response curve from which we extract C_{ij} through least-squares fitting. This measurement protocol requires approximately 30 seconds per

coupling coefficient, and the extracted coefficients typically range from C_{ij} = 0.001–0.01 for nearest neighbours. This weak coupling regime validates our linearization approach and ensures that iterative calibration converges rapidly.

With crosstalk coefficients determined, the central challenge becomes computing compensation voltages that counteract inter-device coupling. We seek correction voltages ΔV_i that, when applied alongside nominal settings, produce the target phase shifts $\phi_{i,\text{target}}$ despite crosstalk interference. We solve this compensation problem through perturbative expansion, exploiting the weak coupling regime ($C_{ij} \ll C_{ii}$) to achieve rapid convergence. The method proceeds in two stages: zero-order approximation followed by first-order correction.

Zero-order approximation. Initially ignoring neighboring devices (j ≠ i), we solve for the baseline voltage $V_i^{(0)}$ from the self term:

$$\phi_{i,\text{target}} - c_i = C_{ii}\left(a_i\left(V_i^{(0)}\right)^2 + b_i V_i^{(0)}\right) \tag{S3 − 20}$$

This quadratic gives the physically valid root:

$$V_i^{(0)} = \begin{cases} \dfrac{-b_i + \sqrt{b_i^2 + 4a_i \dfrac{\phi_{i,\text{target}} - c_i}{C_{ii}}}}{2a_i}, & a_i \neq 0 \\ \dfrac{\phi_{i,\text{target}} - c_i}{C_{ii} b_i}, & b_i \neq 0 \text{ and } a_i = 0 \end{cases} \tag{S3 − 21}$$

First-order correction. Reintroducing neighbouring devices (fixed at their zero-order voltages $V_j^{(0)}$), we compute the correction ΔV_i that compensates for crosstalk:

$$\phi_i = c_i + \sum_{j\neq i} C_{ij}\left(a_j\left(V_j^{(0)}\right)^2 + b_j V_j^{(0)}\right) + C_{ii}\left(a_i\left(V_i^{(0)} + \Delta V_i\right)^2 + b_i\left(V_i^{(0)} + \Delta V_i\right)\right) \tag{S3 − 22}$$

Taylor expanding the self term at $V_i^{(0)}$ and neglecting $(\Delta V_i)^2$:

$$a_i\left(V_i^{(0)} + \Delta V_i\right)^2 + b_i\left(V_i^{(0)} + \Delta V_i\right) \approx a_i\left(V_i^{(0)}\right)^2 + b_i V_i^{(0)} + \left(2a_i V_i^{(0)} + b_i\right)\Delta V_i \tag{S3 − 23}$$

Imposing $\phi_i = \phi_{i,\text{target}}$ gives:

$$C_{ii}\left(2a_i V_i^{(0)} + b_i\right)\Delta V_i + \sum_{j\neq i} C_{ij}\left(a_j\left(V_j^{(0)}\right)^2 + b_j V_j^{(0)}\right) = 0 \tag{S3 − 24}$$

Therefore, the one–step compensation is:

$$\Delta V_i = -\frac{\sum_{j\neq i} C_{ij}\left(a_j\left(V_j^{(0)}\right)^2 + b_j V_j^{(0)}\right)}{C_{ii}\left(2a_i V_i^{(0)} + b_i\right)} \tag{S3 − 25}$$

POI algorithm

Crosstalk characterization establishes precise phase control for individual U(2) units. Building on this foundation, we implement perturbed optimization iteration to calibrate the collective response of all units simultaneously. This approach enables high-fidelity implementation of matrix multiplication.

The POI algorithm operates through systematic parameter perturbation and loss evaluation. For each optimization epoch, we execute the following procedure:

Parameter perturbation. Each parameter ϕ_i in the configuration phase vector $\Phi = [\phi_1, \phi_2, ..., \phi_i, ...]$ undergoes symmetric perturbation by ±δ. We set δ = 0.02 × 2π, corresponding to 2% of the phase shifter's full tuning range, balancing gradient accuracy against measurement noise.

Gradient estimation. We quantify the implementation accuracy using the normalized squared Frobenius error between the measured and target matrices:

$$\mathcal{L}(\mathbf{\Phi}) = \frac{1}{n^2}\sum_{p=1}^{n}\sum_{q=1}^{n} | [w_{pq}]_{\text{measured}} - [w_{pq}]_{\text{target}} |^2 = \frac{1}{n^2} \| \mathbf{W}_{\text{measured}}(\mathbf{\Phi}) - \mathbf{W}_{\text{target}} \|_F^2. \quad \text{(S3-26)}$$

Here, $\mathbf{W}_{\text{target}}$ is the normalized target matrix, $\mathbf{W}_{\text{measured}}(\mathbf{\Phi})$ is the normalized measured matrix realized by the photonic mesh, and

$$\mathbf{\Phi} = [\phi_1, \phi_2, \dots, \phi_N]^{\mathrm{T}}$$

denotes the vector of tunable phase parameters.

For the i-th phase parameter, the corresponding gradient component is estimated using a central finite-difference perturbation. The two perturbed phase configurations are defined as

$$\mathbf{\Phi}_i^{+} = [\phi_1, \dots, \phi_i + \delta, \dots, \phi_N]^{\mathrm{T}},$$

and

$$\mathbf{\Phi}_i^{-} = [\phi_1, \dots, \phi_i - \delta, \dots, \phi_N]^{\mathrm{T}},$$

where only the i-th phase parameter is perturbed while all other phase parameters remain unchanged. The finite-difference estimate of the i-th gradient component is then given by

$$g_i = \frac{\mathcal{L}(\mathbf{\Phi}_i^{+}) - \mathcal{L}(\mathbf{\Phi}_i^{-})}{2\delta} \approx \frac{\partial \mathcal{L}}{\partial \phi_i}. \quad \text{(S3-27)}$$

The phase parameter is updated according to

$$\phi_i \leftarrow \phi_i - \eta g_i, \qquad \text{(S3-28)}$$

where $\eta = 0.02$ is the learning rate, which is empirically selected to ensure stable convergence. Repeating this perturbation-and-update procedure for all tunable phase parameters enables iterative optimization of the photonic mesh toward the target matrix.

Accuracy and ENOB

We quantify accuracy as the absolute error expressed as a percentage of full-scale (%FS): full-scale is the peak-to-peak range.

$$\text{Accuracy (\%FS)} = (1 - \frac{|\text{ error }|}{\text{full-scale}}) \times 100\% \qquad (\text{S3} - 29)$$

This metric reports the error magnitude relative to the instrument's maximum measurable span.

The effective number of bits (ENOB) is derived from the measured mean error μ and the standard deviation of the error σ (both reported in LSB unless stated otherwise). The root-mean-square error (RMSE) is:

$$RMSE = \sqrt{\mu^2 + \sigma^2} \qquad (\text{S3} - 30)$$

ENOB is obtained by comparing the measured RMSE with the rms quantisation error of an ideal uniform quantiser:

$$ENOB = \log_2 \left(\frac{range}{RMSE \cdot k}\right) \qquad (\text{S3} - 31)$$

Here, range is the span of the ideal output levels used for evaluation (max − min; in the same units as RMSE), and k is a distribution-dependent constant: for a uniform distribution over the span, $k = \sqrt{12}$; for a full-scale sinusoidal input, $k = 2\sqrt{2}$. If errors are expressed in LSB on a nominal N_{nom}-bit scale (so range $= 2^{N_{\text{nom}}}$ LSB):

$$ENOB = N_{\text{nom}} - log_2 \left(RMSE_{\text{LSB}} \cdot k\right) \qquad (\text{S3} - 32)$$

3.6 Micro-transfer printed semiconductor optical amplifiers

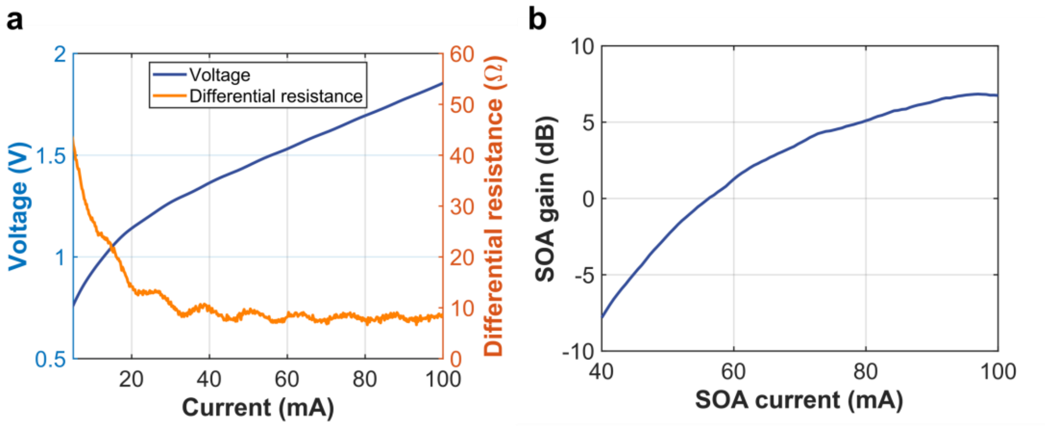

Fig. S3-7 | Characterization of micro-transfer printed SOAs. a, Voltage-current characteristics and differential resistance of a representative SOA. b, On-chip gain of the SOA as a function of bias current

The micro-transfer printed semiconductor optical amplifiers (SOAs) were characterised at 1550 nm wavelength with precise temperature control at 19°C using a thermoelectric cooler. Figure S3-7a presents the voltage-current characteristics of a representative device, revealing a low differential resistance of 8.7 Ω at 100 mA bias current.

Figure S3-7b demonstrates the optical gain performance as a function of bias current. The SOA exhibits a transparency current of 56 mA. At 100 mA, the device delivers 6.9 dB measured on-chip gain. After de-embedding the passive coupling and routing losses, the intrinsic SOA gain is estimated to be approximately 16 dB, sufficient for practical photonic integrated circuit applications. This performance metric validates the micro-transfer printing technique's capability to preserve the intrinsic gain characteristics of III-V materials whilst enabling heterogeneous integration on silicon photonic platforms.

The measured gain-current relationship follows the expected logarithmic dependence, with saturation beginning above 120 mA due to carrier heating effects. These results demonstrate that micro-transfer printed SOAs maintain performance comparable to conventionally bonded devices, whilst offering superior manufacturing scalability and placement flexibility.

3.7 Dynamic calibration

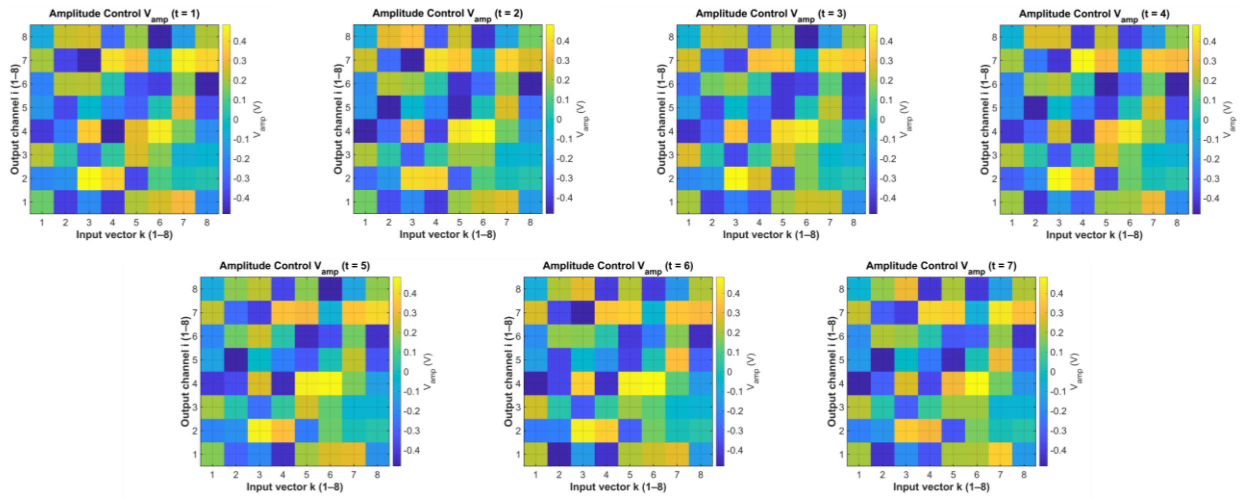

Fig. S3-8 | Dynamic amplitude calibration. 8×8 amplitude-control voltage matrix V_{amp} at RF port for time step t = 1..7. Rows index output channels $i = 1..8$; columns index input vectors $k = 1..8$. Colors encode voltage in volts.

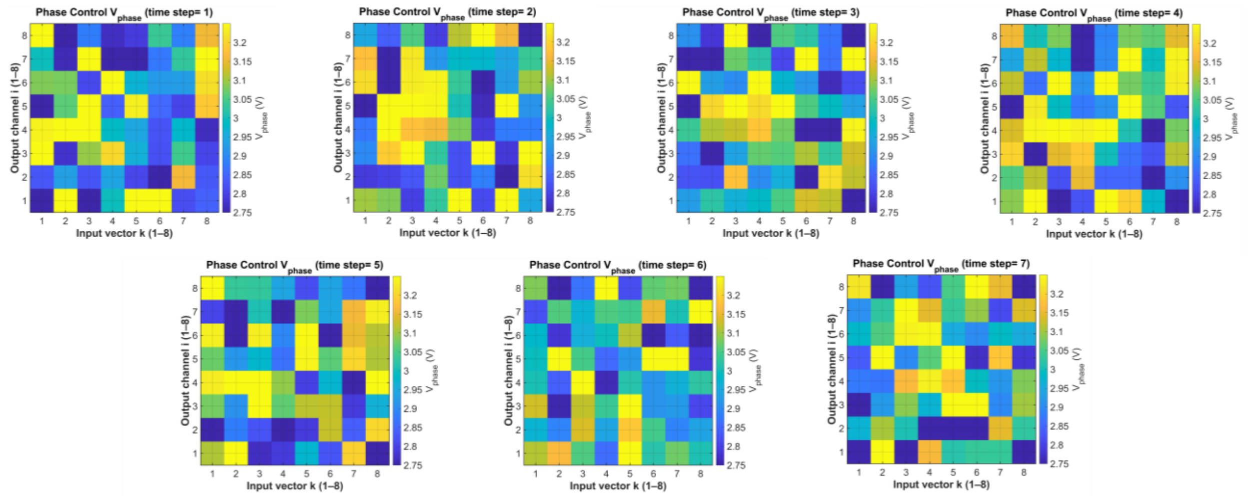

Fig. S3-9 | Dynamic phase calibration. 8×8 phase-control voltage matrix V_{phase} for time step t = 1..7. Rows index output channels $i = 1..8$; columns index input vectors $k = 1..8$. Colors encode voltage in volts.

Device non-uniformity and input-dependent propagation paths within the 8×8 MZI mesh introduce two independent error components at each output port. Phase errors arise from variations in optical path length, refractive index fluctuations, and thermal gradients across the chip. Amplitude errors stem from excess loss, deviation from ideal coupling ratios, and path-dependent attenuation through the mesh network.

To mitigate this the, each output port is followed by one phase EOM and one amplitude EOM. For the k-th injected 8D input vector $\mathbf{x}_k \in \mathbb{C}^8$, the output yields $\mathbf{y}_k \in \mathbb{C}^8$. A diagonal, per-output post-compensation:

$$\mathbf{y}_k' = \mathbf{D}_k\,\mathbf{y}_k, \mathbf{D}_k = \mathrm{diag}\left(a_{1,k}e^{j\phi_{1,k}}, \dots, a_{8,k}e^{j\phi_{8,k}}\right) \quad (S3-33)$$

is implemented, where $\phi_{i,k}$ is set by the phase EOM and $a_{i,k} \geq 0$ by the amplitude EOM of output i.

Figures S3-8 and S3-9 present the calibration voltage matrices for amplitude and phase control, respectively, across time steps t = 1 to 7. Each 8×8 matrix maps the control voltages required when injecting different input vectors (columns k = 1..8) to achieve uniform response across all output channels (rows i = 1..8). The amplitude EOMs operate with a 1.14 V bias voltage, with RF control voltages ranging from -0.4 V to +0.4 V (Fig. S3-8). This voltage range compensates for port-to-port and path-dependent loss variations up to approximately 5 dB. The phase EOMs, which lack a separate bias port, require RF voltages between 2.75 V and 3.25 V (Fig. S3-9), correcting phase non-uniformities within ±7 degrees. Notably, the control voltages fluctuate over time, with phase voltages exhibiting slightly larger variations than amplitude voltages. This difference is modest and reflects the optical phase's higher sensitivity to environmental perturbations. This Dynamic calibration scheme substantially enhances computational accuracy, as demonstrated by the improved matrix operation fidelity shown in the main text (Fig. 3d).

3.8 Phase stabilization

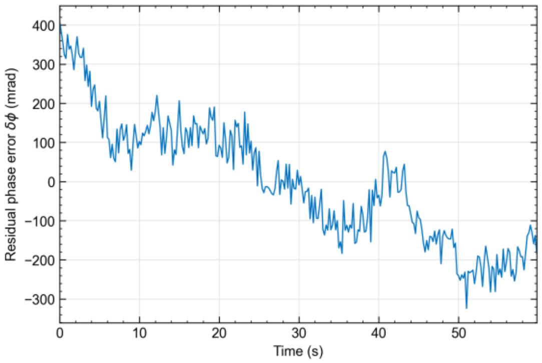

Fig. S3-10| Residual phase fluctuation under closed-loop stabilization.

In our implementation, phase stabilization is achieved through a combination of passive environmental stabilization and active electronic feedback. First, equal-length optical fibers are used to ensure temporal alignment of the optical pulses at the recombination point. The fiber paths are fixed on an air-floated optical bench to suppress vibration-induced optical-path-length fluctuations. During the measurement, the ambient temperature is also kept stable to reduce thermally induced changes in the fiber refractive index and physical length. These passive measures reduce phase drift and mechanical perturbations in the external optical paths.

In addition to these passive measures, the relative phase between the reference arm and the measured branch is actively stabilized using an electronic feedback system based on a ZYNQ-7020 FPGA and an

AD9361 RF transceiver module. A weak pilot/reference signal is introduced to monitor the phase drift of the external optical path. This pilot signal does not participate in the main optical computation, but serves as a phase-monitoring signal for the interferometric readout. After photodetection, the pilot interference signal is converted into an electrical signal, digitized by the AD9361 receiver, and processed in real time on the ZYNQ-7020.

The detected pilot signal can be expressed as:

$$s(t) = A\cos(\omega_p t + \Delta\phi(t)) \qquad (S3-34)$$

where $\omega_p$is the pilot modulation frequency and $\Delta\phi(t)$denotes the relative phase drift between the reference arm and the measured branch. This phase drift can arise from fiber-length fluctuations, thermal variations, mechanical vibration, and polarization-dependent changes in the external optical path. In the FPGA, digital down-conversion and I/Q demodulation are performed by mixing the detected signal with locally generated in-phase and quadrature references. After low-pass filtering, the demodulated components are given by:

$$I(t) \propto A\cos[\Delta\phi(t)], Q(t) \propto A\sin[\Delta\phi(t)] \qquad (S3-35)$$

The instantaneous phase error is then extracted as:

$$\Delta\phi(t) = \mathrm{atan2}(Q(t), I(t)) \qquad (S3-36)$$

The recovered phase-error signal is fed into a digital PI/PID feedback controller implemented on the ZYNQ-7020. The controller generates a correction signal through the AD9361 transmit/DAC path, which is then applied to the phase-control element in the external optical path. In this loop, the proportional term provides immediate correction of the measured phase error, while the integral term suppresses slow residual offsets caused by thermal drift and fiber-length variations. When required, the derivative term can also be used to damp faster perturbations and improve loop stability. The feedback loop maintains the interferometric operating point close to the quadrature locking condition $\Delta\phi \approx \frac{\pi}{2}$, where the phase sensitivity is maximized. By continuously monitoring the pilot signal and applying real-time correction, the system suppresses long-fiber-induced phase fluctuations and maintains a stable relative phase during the measurement window.

Supplementary Fig. S3-10 shows the residual phase fluctuation of the homodyne readout under closed-loop stabilization. Over a 60-s measurement window, the phase error remains within a limited range of a few hundred milliradians and does not exhibit unstable drift or loss of lock. This level of stability is sufficient to support the reported homodyne measurements.

Supplementary Note 4: Linear mode configuration

4.1 Experimental Configuration

SOI and SiN platforms adopt comparable configurations, a detailed discussion of which is omitted for brevity.

Optical Signal Generation and Input

A tunable laser (Thorlabs) provides 1550-nm continuous-wave light at an output power of 10 dBm. Polarisation controllers (Thorlabs FPC030) are used to align the input polarisation to the chip. Intensity modulators (Thorlabs LNA6213), driven by arbitrary-waveform generators (Tektronix AWG5204) and amplified by RF amplifiers (SHF S807Cs), generate ns-scale optical pulses for time-domain computations. Optical isolators (Thorlabs IO-G-1550-APC) are inserted in the optical paths to suppress back-reflected light from propagating into upstream optical components. Tunable optical delay lines, together with the timing control of the AWG, are used to synchronise the arrival times of different optical pulses.

Chip Packaging and Thermal Stabilisation

The photonic chips are bonded to metal heatsinks and cured before electrical packaging. Wire bonds connect the on-chip electrodes to the printed circuit board. A thermoelectric cooler is used to maintain thermal stability, with its cold side attached to the bottom surface of the heatsink and its hot side connected to the thermal baseplate. An embedded negative-temperature-coefficient thermistor monitors the heatsink temperature, enabling temperature-controlled operation during the experiments.

On-Chip Signal Processing and Expansion Interfaces

The optical pulses are coupled into the photonic processor and processed by the programmed on-chip photonic units. On-chip expansion interfaces provide optical access to external auxiliary devices, enabling loss compensation, phase stabilisation, pulse synchronisation, nonlinear activation, and dynamic calibration according to the specific operation mode. The detailed configurations for different operating modes are provided in Supplementary Notes 3–5.

Off-Chip Auxiliary Processing

External auxiliary devices are connected through the on-chip expansion interfaces. EDFAs (Connect MARS) and SOAs are used to compensate optical losses in the loop. They can also serve as nonlinear activation elements, where photodetection-based electronic feedback adjusts the EDFA pump current or the SOA bias current to control the gain-saturation transfer function. Optical filters (WL Photonics, 0.1-nm

3-dB bandwidth) are used after optical amplification to suppress amplified spontaneous emission noise. The external electro-optic modulator provides the high-speed actuation required for the physical implementation of the iteration-dependent correction coefficient c_k. The electronically generated correction signal is applied to the EOM drive port and mapped onto the circulating optical pulse as a controlled amplitude and/or phase modulation, enabling iteration-by-iteration correction before the signal re-enters the photonic processor. More details can be found in Section 3.7.

Phase Stabilisation and Detection

The phase-stabilisation setup consists of equal-length reference and measurement fibres mounted on an air-floated optical bench. Temperature-controlled operation is used to reduce environmental drift. A weak pilot/reference signal is introduced for phase monitoring. The detected interference signal is digitised by an AD9361 RF transceiver and processed by a ZYNQ-7020 FPGA, which generates the feedback signal. After RF amplification, this feedback signal drives phase modulators (Thorlabs LN65S-FC) to actively stabilise the optical phase. Balanced photodetectors (Thorlabs BDX-series, 0–5 GHz bandwidth) receive the output signals through matched optical delay lines, and a 4-channel oscilloscope (Teledyne LeCroy WavePro 804HD) records the system outputs. More details can be found in Section 3.8.

System Control and Data Acquisition

A Python-controlled microcontroller (STM32) operates a 450-channel digital-to-analogue converter system based on AD5370 chips through the SPI protocol. Pre-characterised lookup tables convert the desired photonic states, matrix parameters, bias points, and calibration settings into voltage commands. The DAC outputs are amplified by custom driver circuits before being applied to the corresponding photonic and electronic control elements. Real-time output data are recorded by the oscilloscope and transferred to the computer through the VISA protocol, enabling closed-loop optimisation, calibration, and result extraction.

4.2 Spectral information Acquisition

Moisture Determination Method

Following ISO 712:2010 international standard, we employed oven-drying methodology to determine flour moisture content:

1.Sample preparation: Weigh 5.0000 ± 0.0001 g flour samples using an analytical balance (0.0001 g precision). Distribute samples uniformly in weighing dishes.

2.Drying procedure: Heat samples at 130°C for 90 minutes in a convection oven. Transfer immediately to a desiccator and cool to room temperature (30 minutes). Record sample mass.

Constant mass verification: Repeat step 2 until achieving constant mass (successive weighings differ by ≤0.0003 g).

3.Moisture calculation: Calculate moisture content as: [(initial mass - dry mass)/initial mass] × 100%.

Sample Preparation with Gradient Moisture Contents

We purchased multiple commercial high-gluten flour brands from local markets. Samples underwent controlled dehydration at 50°C, with aliquots removed at 0.5, 1, 2, 4, and 6 hours. Each sample cooled in a desiccator for 10 minutes before analysis, yielding flour samples with gradient moisture contents.

Spectral and moisture data acquisition

1.Pre-treatment: All flour samples underwent compaction before spectral acquisition to minimise density variations and air gap interference.

2.Untreated samples: Fresh flour samples received immediate near-infrared transmission spectroscopy after compaction. Moisture content was determined using the validated method above.

3.Gradient samples: Dehydrated samples were removed from desiccators, compacted, and analysed immediately via NIR spectroscopy. Following spectral acquisition, moisture content was determined using the standard protocol.

This experimental workflow generated flour samples spanning a moisture gradient, with corresponding NIR spectra and validated moisture measurements for each sample.

4.3 Spectral data analysis

For a standard linear system data with variation, the expression is given as:

$$\boldsymbol{y} = \boldsymbol{X\beta} + \boldsymbol{\epsilon} \qquad (\mathrm{S4}-1)$$

Where $\mathbf{y}$ can be considered as the linear system output, X is the input and ϵ is the associated deviation from the perfectly linear projection. If we consider a simple least squared error minimizing estimator, the corresponding form is given as:

$$\widehat{\beta} = \left(\mathbf{X^T X}\right)^{-1} \mathbf{X^T y} \qquad (\mathrm{S4}-2)$$

A commonly known issue with the direct least squared estimator is overfitting the prediction model to the training dataset. However, for real life data, there sometimes exists a systematic mismatch between the training and testing dataset. This can be the result of various factors. For example, systematic environment change, or equipment change can cause this discrepancy. To compensate for this issue and obtain a "good enough" prediction model, ridge regression (also known as Tikhonov regularization) is a modified estimator with a deliberate error term to avoid overfitting.

$$\widehat{\beta_{\mathrm{ridge}}} = \left(\mathbf{X^T X} + \boldsymbol{\lambda I}\right)^{-1} \mathbf{X^T y} \qquad (\mathrm{S4}-3)$$

Here I is the appropriate identity matrix and λ is a scalar hyperparameter controlling the degree of deliberate error added to the estimator. For the context of the linear TPPP, the inversion part is the accelerated procedure for the overall processing.

The original spectrum data acquired has 1899 channels for each sample point. This is a dimensionality that is much higher than that of our TPPP device (8 ports) and can't be directly used onto the TPPP device. Principal component analysis (PCA) is a helpful linear dimensionality reduction technique that can preserve majority of the original information by finding the largest variation. When the original data's many elements have strong joint variability (often due to physical implications), then PCA is highly suitable.

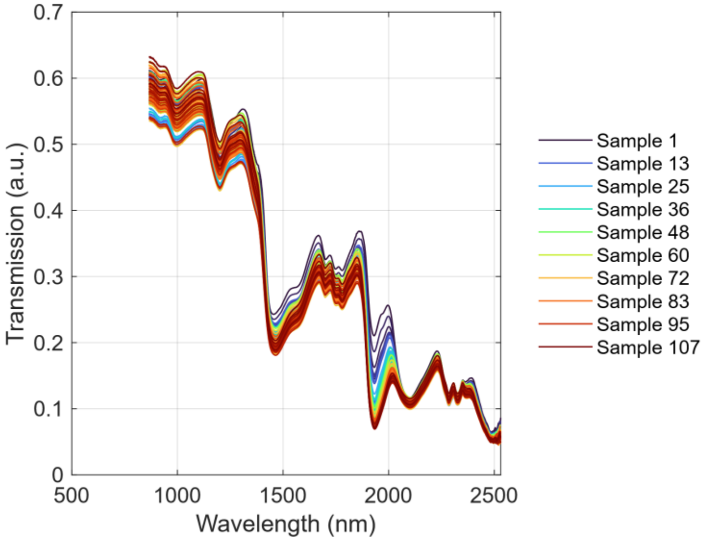

Fig. S4-1 | Original spectrum plots of the samples**.**

The original spectrum data of the flour samples is shown in Fig. S4-1. Assuming all other variables controlled, then the spectrum should only see effects from the humidity. While not technically analogous to the "water absorption peak", we know that some wavelengths would see joint variation while some other wavelengths hardly move.

When performing the PCA, typical algorithms starts with the most significant component then work its way through until achieving the number of components desired. The first component of PCA can be described as:

$$\mathbf{w}(1) = \operatorname{argmax} \frac{\mathbf{w}^{\mathbf{T}}\mathbf{X}^{\mathbf{T}}\mathbf{X}\mathbf{w}}{\mathbf{w}^{\mathbf{T}}\mathbf{w}} \qquad (\mathrm{S4}-4)$$

The first PCA component is a unit vector that can be intuitively understood as the direction that the data is most "skewed" in. The later PCA components are found by repeating the process when all previous components have been subtracted from the original **X**. The PCA-reduced spectrum can be found it Fig. S4-2.

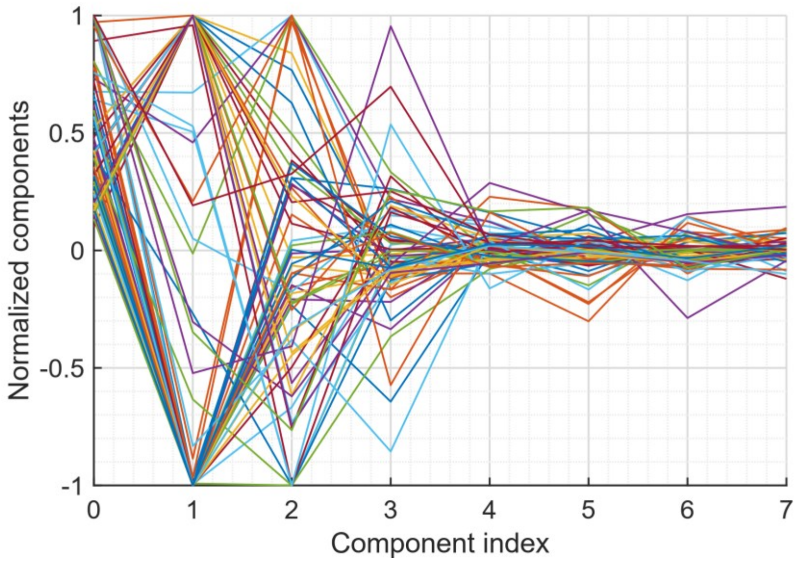

Fig. S4-2 | The normalized components before processed by the TPPP.

One detail to keep in mind is that while the device's 8 channel intuitively calls for reduction with the first eight components, in practice we only use the first seven. The reason is that we are reserving a specific dummy channel for the axis interception for systems that doesn't necessarily pass through the origin. As can be seen in Fig. S4-2, the number 0 index component for all the samples are set to 0.1. Theoretically, this could be any number and will not affect the estimator computation. For the experiment, because the component variations are normalized within -1 to 1 with respect to the first component, a moderate value less than 1 is chosen.

When normalizing the components' values, it is required to normalize the components together rather than separately because the relative magnitude of variation is important. The performance of the linear regressor with uncalibrated (88%) and dynamically calibrated (95%) systems are shown in Fig. 3(h) of the main article.

As briefly highlighted in the main article, aside from the systematic control inaccuracy, a lossy system can also lead to severely skewed encoding behaviour of RPPs. This has been a persisting issue with purely passive recurrent systems. For example, in Fig. S4-3, an estimator computed with a system of high loss is shown in comparison to our lossless compensated system.

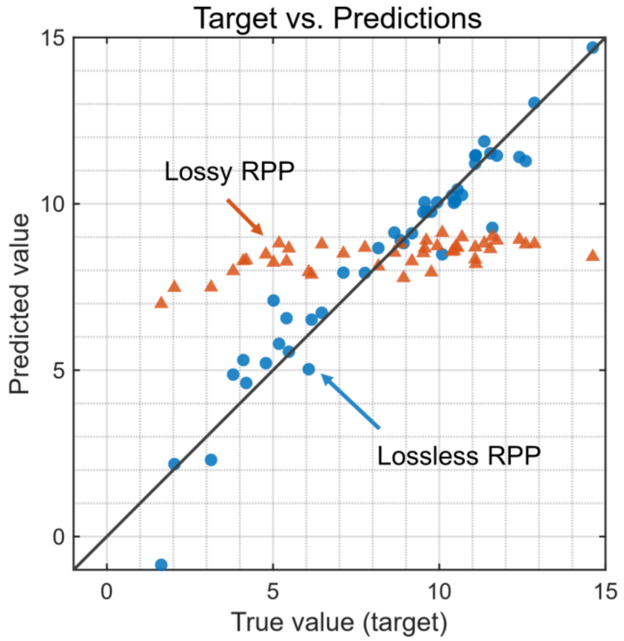

Fig. S4-3 | Comparison between a typical lossy RPP and our SOA-enabled lossless RPP.

Systems of high loss immediately experience high level of performance degradation due to the skewness. Fig. S4-3 shows that the prediction value is close to random guessing. When inserting these two estimators for the binary classifier, the lossy system is only able to achieve rough 67% accuracy compared to roughly 95% with the TPPP.

4.4 Representative Input–Output Samples

The matrix encoded in the matrix multiply core is:

$$\begin{bmatrix} 0.396 & 0.022 & 0.001 & -0.004 & -0.004 & 0 & -0.003 & 0 \\ 0.023 & -0.422 & 0.100 & -0.050 & -0.006 & 0.010 & -0.008 & 0.006 \\ 0.001 & 0.100 & 0.391 & -0.004 & 0.002 & 0.002 & 0.004 & 0 \\ -0.004 & -0.050 & -0.004 & 0.428 & 0 & 0.001 & 0 & 0 \\ -0.004 & -0.006 & 0.002 & 0 & 0.437 & 0 & 0 & 0 \\ 0 & 0.010 & 0.002 & 0.001 & 0 & 0.437 & 0 & 0 \\ -0.003 & -0.008 & 0.004 & 0 & 0 & 0 & 0.437 & 0 \\ 0 & 0.006 & 0 & 0 & 0 & 0 & 0 & 0.437 \end{bmatrix} \quad (S4-5)$$

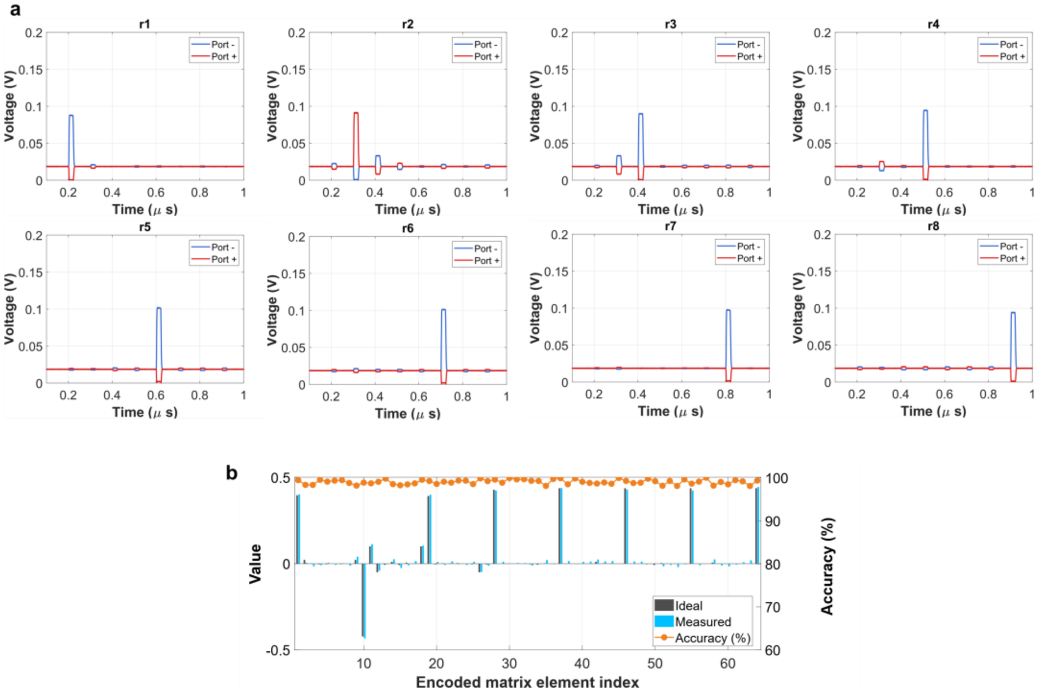

Fig. S4-4 | Characterization of encoding accuracy for the matrix inversion operator.

a, Time-domain pulse signals recorded at output ports 1–8, representing the encoded matrix elements of rows 1–8. **b,** Ideal versus experimentally measured encoded matrix element values and the corresponding accuracy assessment.

Following successful encoding of the matrix inversion operator, we validated the encoding accuracy. We applied sequential input pulses of 20 ns duration at 100 ns intervals to each port, effectively implementing multiplication of an identity matrix with the encoded matrix in the photonic matrix multiplication core. This approach provides direct readout of the encoded matrix element values through temporal demultiplexing.

Figure S4-4a displays the time-domain pulse trains recorded at output ports 1–8. Each port's output waveform corresponds matrix row elements: the i-th port captures row i of the encoded matrix, with the j-th pulse representing matrix element ω_{ij}.

Figure S4-4b quantifies the encoding fidelity by comparing ideal theoretical values with experimentally measured matrix elements. The achieved accuracy exceeds 98%, demonstrating exceptional preservation of numerical precision throughout the optical encoding process. Statistical analysis across three independent measurements yields the mean values and standard deviations presented in Figure 3b of the main text, confirming reproducibility and stability of the encoding process.

After successful encoding verification, we start matrix inversion calculations using the configuration detailed in Section 4.1. The initial state $\boldsymbol{X}_0 = \alpha I$, encoded with pulse was injected into the chip. To validate

operational stability, we employed rectangular input pulses with 50-ns duration, leveraging their flat-top profiles as stability indicators.

The theoretical result for this measurement should yield:

$$\begin{bmatrix} 0.931 & 0.015 & 0.004 & -0.008 & -0.006 & 0 & -0.005 & 0.001 \\ 0.015 & 0.402 & 0.066 & -0.036 & -0.004 & 0.008 & -0.005 & 0.004 \\ 0.004 & 0.066 & 0.935 & -0.013 & 0.003 & 0.004 & 0.005 & -0.001 \\ -0.008 & -0.036 & -0.013 & 0.985 & 0 & 0.001 & 0.001 & -0.001 \\ -0.006 & -0.004 & 0.003 & 0 & 0.998 & 0 & -0.001 & 0 \\ 0 & 0.008 & 0.004 & 0.001 & 0 & 0.998 & -0.001 & 0 \\ -0.005 & -0.005 & 0.005 & 0.001 & -0.001 & -0.001 & 0.998 & 0 \\ 0.001 & 0.001 & -0.001 & -0.001 & 0 & 0 & 0 & 0.998 \end{bmatrix} \quad (S4-6)$$

Figures S4-5 and S4-6 present the pulse waveform data acquired by the detection module throughout the iterative process. They demonstrate the temporal evolution of output pulses. The maintained rectangular profile integrity confirms the system's robustness against noise. Notably, the pulse amplitude follows the predicted convergence trajectory, with successive iterations showing decay towards the 0. Time spacing of iterations is approximately 240 ns, determined by the experimental optical loop length. The first pulse of each diagonal element corresponds to the input initial state. These eight diagonal elements exhibit nearly identical first pulses, validating our detection channel equalization method. Whilst elements theoretically equal to zero display residual pulse fluctuations due to inevitable system imperfections, these fluctuations remain below our convergence threshold. Signal-to-noise ratio deteriorates progressively with iteration count, attributable to amplified spontaneous emission (ASE) noise introduced during optical amplification and environmental noise—consistent with findings in System Characterization of the main text.

The ideal versus experimentally measured inverse matrix element values and the corresponding accuracy assessment under dynamic calibration are given in Figure S4-7, revealing an accuracy exceeding 97%. Statistical analysis across three independent measurement sets yields robust performance metrics, with mean values and standard deviations detailed in Figure 3c. The system maintains a mean accuracy of 95%. This high fidelity stems from our previously described calibration methodology, which compensates for device-specific variations and systematic errors. These results establish our photonic processor as a viable platform for high-precision matrix computations.

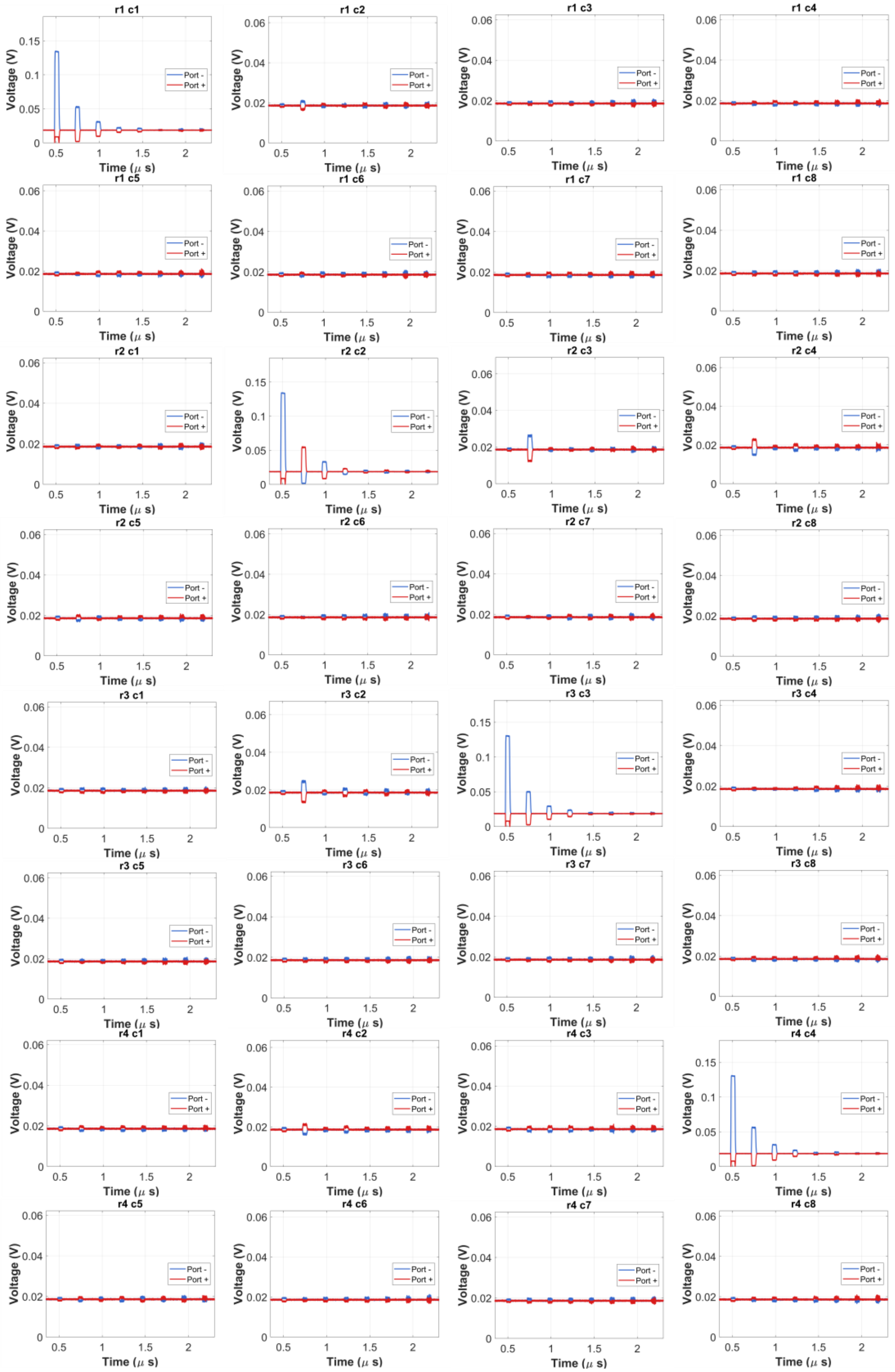

Fig. S4-5 Representative recorded waveforms (Part 1) demonstrating a matrix inversion operation with the TPPP system.

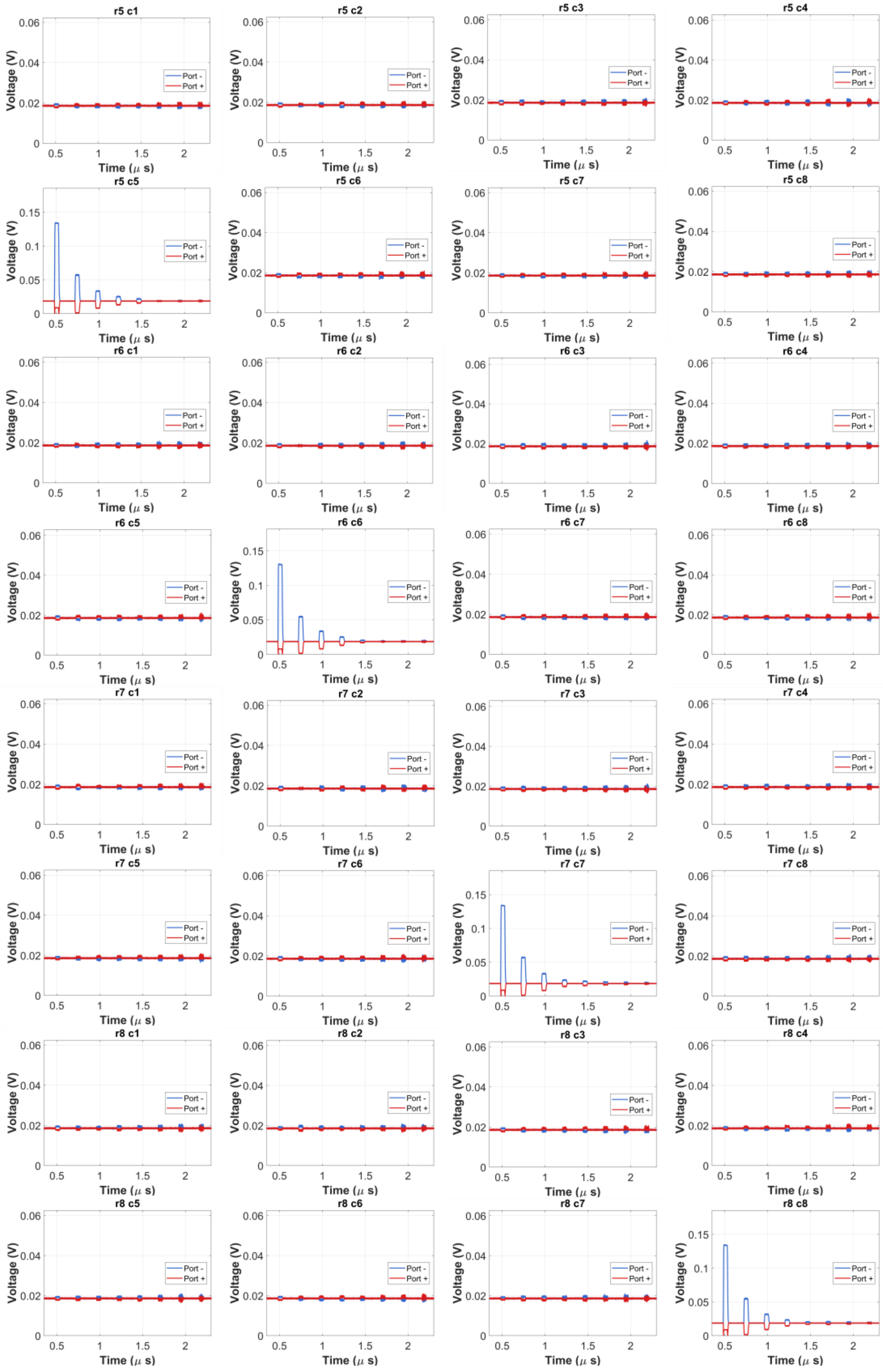

Fig. S4-6 Representative recorded waveforms (Part 2) demonstrating a matrix inversion operation with the TPPP system.

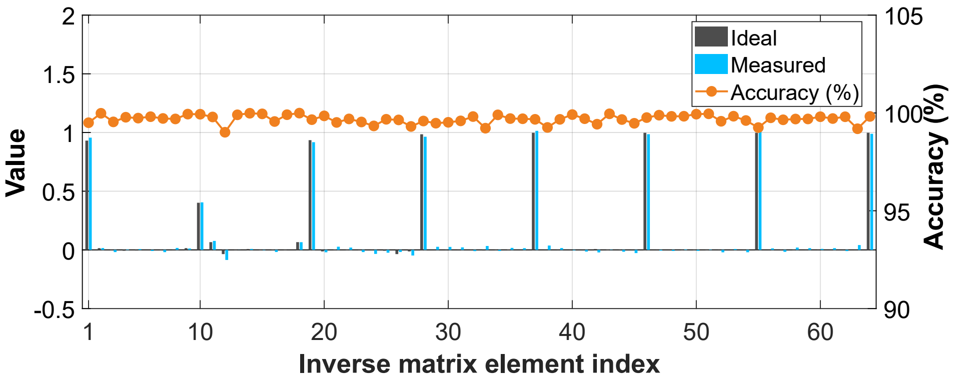

Fig. S4-7 Ideal versus experimentally measured matrix element values and the corresponding accuracy assessment.

Supplementary Note 5: Nonlinear mode configuration

5.1 Experimental Configuration

Whilst the input modulation, auxiliary off-chip processing, and detection modules are similar to those used in the linear configuration described above, the optical routing is reconfigured for the TA-RNN operation. The key distinction is that the singular values are dynamically modulated in the optical domain, enabling time-dependent input-to-hidden and hidden-to-hidden transformations. The TA-RNN therefore uses two optical processing paths: one for the current input state and one for the recurrent hidden state.

Input State Processing Path

The sensing data, encoded as optical pulse signals, enter the chip through expansion interface. A tap coupler extracts a small portion of the signal for real-time monitoring, while the remaining signal is routed to AMU core. In this path, AMU core is programmed to implement the right singular-vector matrix $V_{\mathrm{ih}}^{T}$of the input-to-hidden weight matrix. The transformed optical signal is then routed out of the chip through expansion interface for auxiliary processing.

In the external processing chain, EDFAs and SOAs compensate optical losses accumulated in the chip and fibre links. Optical filters with a 0.1-nm 3-dB bandwidth are used to suppress amplified spontaneous emission noise after optical amplification. Programmable electro-optic modulators then apply the time-varying singular-value weights $S_{\mathrm{ih}}(t)$. In this way, the input path optically implements the transformation associated with $S_{\mathrm{ih}}(t)V_{\mathrm{ih}}^{T}$, where the singular values can be updated dynamically from one time step to the next.

Hidden State Recurrent Path

The hidden-state signal is initially set to zero and is subsequently updated through the optical feedback loop. At each recurrent step, the hidden-state pulse enters the chip through expansion interface. After tap-coupling for monitoring, the signal is routed through AMU, which is configured to implement the right singular-vector matrix $V_{\mathrm{hh}}^{T}$of the hidden-to-hidden weight matrix. The transformed hidden-state signal is then sent to the external auxiliary chain.

Similar to the input path, the recurrent path uses optical amplification and filtering for loss compensation and noise suppression. Programmable electro-optic modulators apply the dynamic singular-value weights $S_{\mathrm{hh}}(t)$, enabling time-dependent recurrent modulation. This path therefore implements the optical equivalent of $S_{\mathrm{hh}}(t)V_{\mathrm{hh}}^{T}$, providing the adaptive recurrent connection required for temporal sequence processing.

Nonlinear Activation.

Both processed paths converge at UMU core, which implements left singular vector matrices U_{ih} and U_{hh} whilst performing coherent addition. The combined signal undergoes crucial nonlinear activation. In this test, photodetection-based electronic feedback adjusts the EDFA pump current, allowing the gain-saturation transfer function to be tuned during operation.

5.2 Driving model

We developed a lightweight, interpretable driving model that generates realistic control trajectories for validating our photonic computing platform. This rule-based system captures essential human driving behaviours whilst maintaining computational efficiency suitable for hardware-in-the-loop testing. The driving model includes the following critical components:

Environment & perception. The agent moves in a planar world populated with randomly spawned obstacles ahead of the car. At each timestep, it processes a compact, driver-centric observation vector mimicking human visual attention patterns. The system tracks current speed and obstacle distances within three angular sectors: front (±30°), left (60°–120°), and right (−120° to −60°). This abstraction prioritises imminent collision risks and lateral escape routes, the primary cues guiding human drivers. The vehicle state comprises position, heading and velocity $(x_t, y_t, \theta_t, v_t)$. Let $\mathrm{wrap}(\alpha) = ((\alpha + \pi) \bmod 2\pi)$ and $\mathrm{clip}(z; a, b) = \min\{\max\{z, a\}, b\}$.

Let the set of obstacle points at time t be $O_t = \{(o_k^x, o_k^y)\}$, For each obstacle k,

$$r_k = \sqrt{(o_k^x - x_t)^2 + \left(o_k^y - y_t\right)^2}, \alpha_k = \mathrm{wrap}\left(\mathrm{arctan2}\ \left(o_k^y - y_t, o_k^x - x_t\right) - \theta_t\right) \qquad (\mathrm{S5}-1)$$

With a maximum sensing range $D_{\max}$, the driver-centric distances are:

$$d_F(t) = \min(\left\{r_k : |\alpha_k| \le \frac{\pi}{6}\right\} \cup \{D_{\max}\}) \qquad (\mathrm{S5}-2)$$

$$d_L(t) = \min(\left\{r_k : \frac{\pi}{3} \le \alpha_k \le \frac{2\pi}{3}\right\} \cup \{D_{\max}\}) \qquad (\mathrm{S5}-3)$$

$$d_R(t) = \min(\left\{r_k : -\frac{2\pi}{3} \le \alpha_k \le -\frac{\pi}{3}\right\} \cup \{D_{\max}\}) \qquad (\mathrm{S5}-4)$$

The observation vector is $o_t = [v_t, d_F(t), d_L(t), d_R(t)\ ]$

Human-like "teacher" strategy. Our controller implements three distinct behavioral regimes, transitioning smoothly based on frontal obstacle proximity. This hierarchy reflects natural human decision-making under varying risk levels: (1) **emergency evade** when a frontal obstacle is extremely close, (2) **near-field bypass** guided by the left–right free-space gradient with a mild heading-hold term, and (3) **cruise** with simple heading keeping when the path ahead is clear.

Let the desired heading be $\theta^* = 0$ and the wrapped heading error $e_\theta = \mathrm{wrap}(\theta^* - \theta_t)$. The raw (pre-slew) steering $s_t^{\mathrm{raw}} \in [-1,1]$ and throttle $\tau_t^{\mathrm{raw}} \in [0,1]$ follow three regimes:

(1) Emergency evade ($d_F(t) < 1$ m)

$$s_t^{\mathrm{raw}} = \begin{cases} -1, & d_\mathrm{L} > d_\mathrm{R} \\ 1, & d_\mathrm{L} \le d_\mathrm{R} \end{cases}, \qquad \tau_t^{\mathrm{raw}} = \max(0.2,\ d_\mathrm{F}\ /\ 5) \qquad (\mathrm{S5}-5)$$

(2) Near-field bypass ($1 \le d_F(t) < 5$ m)

$$s_t^{\mathrm{raw}} = \mathrm{clip}\left(\frac{d_R - d_L}{5}, -1{,}1\right) + 0.3e_\theta, \qquad \tau_t^{\mathrm{raw}} = \max(0.2,\ d_\mathrm{F}\ /\ 5) \qquad (\mathrm{S5}-6)$$

(3) Cruise / heading-hold ($d_F(t) > 5$ m)

$$s_t^{\mathrm{raw}} = 0.5e_\theta, \qquad \tau_t^{\mathrm{raw}} = 1 \qquad (\mathrm{S5}-7)$$

Actuator Dynamics and Rate Limiting. Human reaction times and mechanical constraints impose bandwidth limitations on control actions. We enforce slew-rate limits to generate smooth, realistic trajectories. With slew limits:

$$u_s = \mathrm{MAX\_STEER\_CHANGE} \cdot \Delta\mathrm{t}, \qquad u_\tau = \mathrm{MAX\_THROTTLE\_CHANGE} \qquad (\mathrm{S5}-8)$$

the applied controls are:

$$\Delta s_t = \mathrm{clip}(s_t^{\mathrm{raw}} - s_{t-1,} - u_s, u_s), \qquad s_t = \mathrm{clip}(s_{t-1,} + \Delta s_t, -1, +1) \qquad (\mathrm{S5}-9)$$

$$\Delta \tau_t = \mathrm{clip}(\tau_t^{\mathrm{raw}} - \tau_{t-1,} - u_\tau, u_\tau), \qquad \tau_t = \tau_{t-1,} + \Delta \tau_t \qquad (\mathrm{S5}-10)$$

Vehicle response. Controls are converted to motion via simplified Ackermann kinematics mode. With wheelbase L, steering limit $\delta_{max} = \mathrm{MAX\_STER\_DEG} \cdot \pi/180$, and a simple throttle–acceleration map

$$\delta_t = s_t \delta_{\max} \qquad (\mathrm{S5}-11)$$

$$v_{t+1} = \mathrm{clip}(v_t + (\tau_t - 0.2)A\mathrm{CCEL_{FACTOR}}\Delta\mathrm{t}, 0, \mathrm{MAX\_V}) \qquad (\mathrm{S5}-12)$$

$$x_{t+1} = x_t + v_t \cos\theta_t \Delta t,\ y_{t+1} = y_t + v_t \sin\theta_t \Delta t \qquad (\mathrm{S5}-13)$$

$$\theta_{t+1} = wrap\left(\theta_t + \frac{v_t}{L}\tan \delta_t\, \Delta t\right) \qquad (\mathrm{S5}-14)$$

5.3 Representative Input–Output Samples

The learned weight matrices $\boldsymbol{W}_{ih}$ and $\boldsymbol{W}_{hh}$ are:

$$\boldsymbol{W}_{hh} = \begin{bmatrix} 0.110 & 0.357 & -0.159 & 0.459 \\ 0.037 & 0.006 & 0.040 & -0.212 \\ -0.157 & 0.344 & 0.112 & 0.448 \\ 0.086 & 0.460 & 0.087 & 0.348 \end{bmatrix} \quad (S5-15)$$

$$\boldsymbol{W}_{ih} = \begin{bmatrix} -0.034 & -0.085 & -0.081 & -0.008 \\ 0.010 & 0.094 & 0.022 & 0.020 \\ -0.033 & -0.084 & -0.009 & -0.077 \\ 0.362 & -0.055 & -0.010 & 0.009 \end{bmatrix} \quad (S5-16)$$

We decompose these matrices using singular value decomposition (SVD), yielding:

$$\boldsymbol{W}_{hh} = \begin{bmatrix} -0.580 & 0.704 & -0.230 & -0.338 \\ 0.153 & -0.016 & 0.654 & -0.741 \\ -0.563 & -0.710 & -0.263 & -0.333 \\ -0.568 & -0.021 & 0.671 & 0.475 \end{bmatrix} \times$$

$$\begin{bmatrix} 0.999 & 0 & 0 & 0 \\ 0 & 0.269 & 0 & 0 \\ 0 & 0 & 0.232 & 0 \\ 0 & 0 & 0 & 0.049 \end{bmatrix} \times \quad (S5-17)$$

$$\begin{bmatrix} -0.019 & -0.661 & -0.014 & -0.750 \\ 0.695 & -0.010 & -0.719 & 0.005 \\ 0.420 & 0.604 & 0.395 & -0.550 \\ 0.583 & -0.445 & 0.572 & 0.367 \end{bmatrix}$$

$$\boldsymbol{W}_{ih} = \begin{bmatrix} -0.061 & -0.608 & 0.701 & -0.368 \\ -0.008 & 0.536 & 0.022 & -0.844 \\ -0.058 & -0.581 & -0.713 & -0.387 \\ 0.996 & -0.067 & 0.002 & -0.051 \end{bmatrix} \times$$

$$\begin{bmatrix} 0.368 & 0 & 0 & 0 \\ 0 & 0.180 & 0 & 0 \\ 0 & 0 & 0.070 & 0 \\ 0 & 0 & 0 & 0.026 \end{bmatrix} \times \quad (S5-18)$$

$$\begin{bmatrix} 0.992 & -0.125 & -0.013 & -0.012 \\ 0.117 & 0.857 & 0.372 & 0.336 \\ -0.032 & 0.039 & -0.708 & 0.704 \\ -0.047 & -0.499 & 0.602 & 0.622 \end{bmatrix}$$

Our photonic architecture implements adaptive singular value modulation across time steps, as detailed in Supplementary note 2. This dynamic adjustment allows the network to selectively amplify or attenuate different temporal features based on input characteristics, enhancing the system's expressivity whilst maintaining computational efficiency.

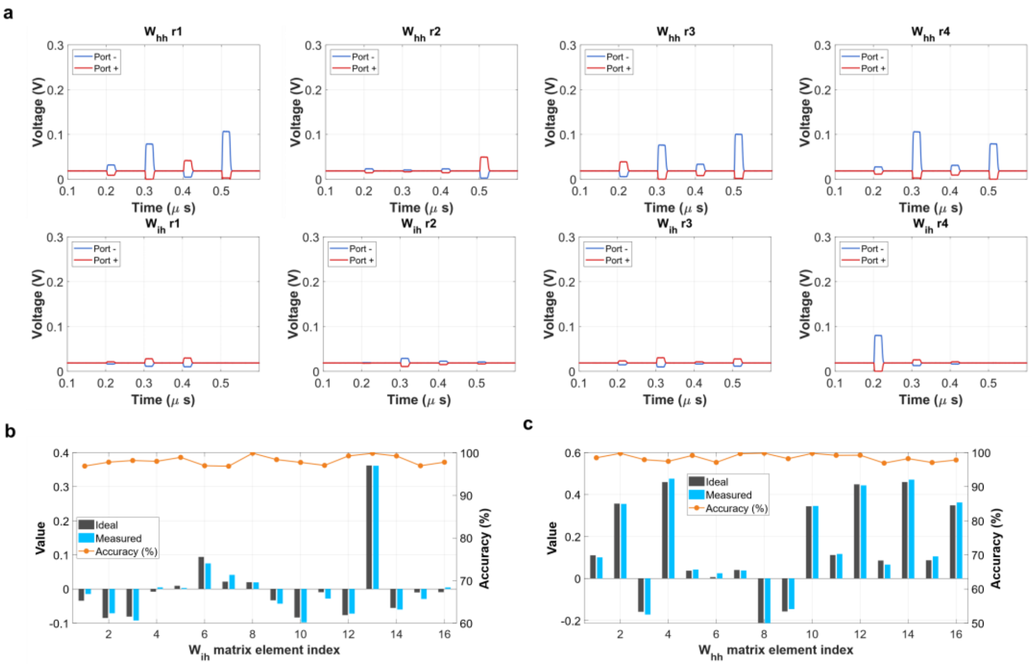

Fig. S5-1 | Characterization of encoding accuracy for $\boldsymbol{W}_{ih}$ and $\boldsymbol{W}_{hh}$.

(a), Time-domain pulse signals recorded at output ports 1–8, representing the encoded matrix elements of rows 1–4 of $\boldsymbol{W}_{ih}$ and $\boldsymbol{W}_{hh}$, respectively. b, Ideal versus experimentally measured encoded matrix element values of (b) $\boldsymbol{W}_{ih}$ and (c) $\boldsymbol{W}_{hh}$ and the corresponding accuracy assessment.

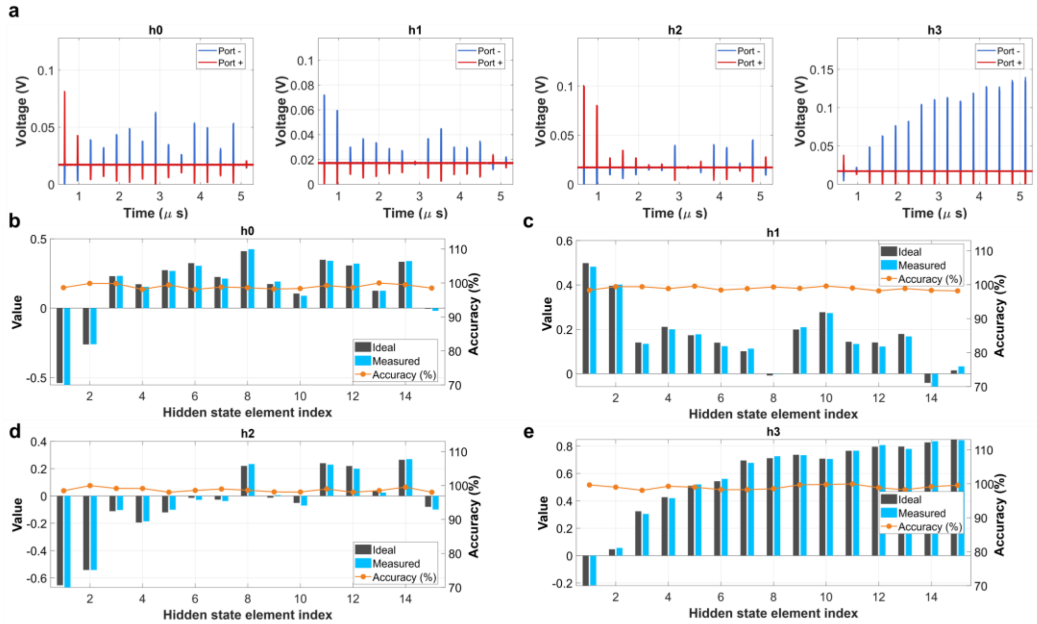

Fig. S5-2 | Characterization of hidden states.

(a), Time-domain pulse signals of hidden states h0, h1, h2 and h3. Ideal versus experimentally measured hidden state values of (b) h0, (c)h1, (d)h2 and (e)h3, and the corresponding accuracy assessment.

We validated the encoding fidelity of weight matrices $\boldsymbol{W}_{\mathrm{ih}}$ and $\boldsymbol{W}_{\mathrm{hh}}$ using time-domain pulse characterisation at the matrix core outputs. Figure S5-1a displays pulse signals recorded at output ports 1–8, representing the encoded elements from rows 1–4 of both matrices. The experimental protocol followed the methodology detailed in Supplementary Note 4. Quantitative assessment revealed high encoding accuracy for both matrices (Figs S5-1b, c), confirming the reliability of our optical encoding scheme for implementing recurrent neural network weight matrices in the photonic domain. Figure S5-2 presents the time-domain pulse signals for hidden states h0, h1, h2 and h3 across four output ports. Figure S5-2(a) displays the temporal evolution of these states, whilst figure S5-2(b)–(e) compare ideal theoretical predictions with experimental measurements for h0, h1, h2 and h3, respectively, including quantitative accuracy assessments. The signal-to-noise ratio decreases systematically with increasing iteration count. This degradation stems from amplified spontaneous emission (ASE) noise accumulated during optical amplification stages and environmental noise contributions. These observations align with the noise analysis presented in System Characterization of the main text and supplementary note 4.

Supplementary Note 6: Performance evaluation and scalability

6.1 Energy Efficiency

For a $1 \times N$ input vector multiplied by an $N \times N$ matrix, we count:

$$\text{ops/s} = R_s \,(2N^2 - N) \qquad (\text{S6}-1)$$

where R_s is the symbol rate (Baud; symbols/s). With total wall-plug electrical power P_{tot}(W), we have:

$$E_{\text{op}} = \frac{P_{\text{tot}}}{\text{ops/s}}[\text{J/op}] \quad E_{\text{op}}[\text{fJ/op}] = 10^{15}\, E_{\text{op}} \qquad (\text{S6}-2)$$

P_{tot} can be decomposed as

$$P_{\text{tot}} = P_{\text{laser}} + P_{\text{SOA}} + P_{\text{weight}} + P_{\text{mod}} + P_{\text{det}} + P_{\text{misc}} \qquad (\text{S6}-3)$$

where P_{laser} is the wall-plug laser power needed to supply the required per-channel optical input; P_{SOA} is the static SOA bias power across all segments; P_{weight} is the thermal tuning (heater/weight) power; P_{mod} is the RF drive power for the N TW-MZMs operating with PAM-M and 50 Ω termination; $P_{\text{det}}$is the receive-chain power (TIA/ADC, etc.) and P_{misc} lumps clocks/DSP/control. In order to calculate each term, we first calculate three quantities used across the model: segmentation count M_{seg}, overall signal transfer T_{sig}, and output ASE PSD S_{out}.

For an $N \times N$ mesh, the depth is D_{layers}. Each layer incurs a per-layer loss ℓ (dB/layer). To prevent any single SOA from approaching or exceeding a per-segment gain cap G_{max}(dB), the mesh is partitioned into segments of at most $k_{\text{layers}} = max(1, \lfloor \frac{G_{\text{max}} - h_{\text{dB}}}{\ell} \rfloor)$ layers. The total number of segments is:

$$M_{\text{seg}} = \left\lceil \frac{D_{\text{layers}}}{k_{\text{layers}}} \right\rceil \qquad (\text{S6}-4)$$

This choice guarantees that the passive loss accumulated within any segment can be compensated plus a small headroom h_{dB}without exceeding G_{max}. Let segment m contain $k_m \in \{1, \dots, k_{\text{layers}}\}$ layers (the last segment may be shorter). Its loss in dB is $\ell_m = k_m\, \ell$(with $k_m \le k_{\text{layers}}$); linear loss is $L_m = 10^{-\ell_m/10}$. The applied gain is $G_m = 10^{\min(\ell_m + h_{\text{dB}},\, G_{max})/10}$. A fixed downstream "tail" loss (couplers, filters, output grating, etc.) is modeled as $L_{\text{tail}} = 10^{-L_{\text{tail,dB}}/10}$. The transmission from the mesh input to the output is the product of per-segment loss/gain and the tail loss:

$$T_{\text{sig}} = \left(\prod_{m=1}^{M_{\text{seg}}} L_m\, G_m \right) L_{\text{tail}} \qquad (S6-5)$$

Each SOA adds amplified spontaneous emission (ASE). For segment m with linear gain G_m, the single-polarisation ASE power spectral density (per unit optical bandwidth) injected after that SOA is modeled as $S_m = n_{sp}\, h\nu\, (G_m - 1)$ .Here h is Planck's constant, $\nu = c/\lambda$ is the optical frequency, and n_{sp} is the

spontaneous-emission factor (Noise figure $\approx 10\log_{10}(2n_{sp})$ dB when both polarizations are collected; in our model the "2" is absorbed into the chosen n_{sp}). This ASE then propagates forward through the remaining segments and the tail. The transfer from the output of segment m to the global output is

$$\Pi_{m\to\text{out}} = \left(\prod_{j=m+1}^{M_{\text{seg}}} L_j\, G_j\right) L_{\text{tail}} \qquad (\text{S6}-6)$$

Summing independent contributions from all segments gives output ASE PSD:

$$S_{\text{out}} = \sum_{m=1}^{M_{\text{seg}}} \left[n_{sp}\, h\nu\, (G_m - 1)\right] \Pi_{m\to\text{out}} \qquad (\text{S6}-7)$$

1.laser power

With target post-filter symbol SNR (SNR$_{\text{sym,target}}$(linear), roll-off ρ, and OSNR reference bandwidth RBW, the required OSNR (OSNR_{req}) is:

$$\text{OSNR}_{\text{req}} = \text{SNR}_{\text{sym,target}} \frac{(1+\rho)\, R_s}{\text{RBW}} \qquad (\text{S6}-8)$$

The ASE power in the reference bandwidth is $P_{\text{ASE,out}} = S_{\text{out}} \cdot \text{RBW}$. The required signal at the output is:

$$P_{\text{sig,out,req}} = \text{OSNR}_{\text{req}} \cdot P_{\text{ASE,out}} \qquad (\text{S6}-9)$$

Therefore, the required wall-plug laser power for N channels is

$$P_{\text{laser}} = \frac{N \cdot P_{\text{sig,out,req}}}{\eta_{\text{laser}} \cdot T_{\text{sig}}}$$

Here η_{laser} is a wall-plug efficiency.

2. SOA bias power

Static per-SOA device bias scales with the number of segments and outputs:

$$P_{\text{SOA}} = N\, M_{\text{seg}}\, P_{\text{SOA,bias}} \qquad (\text{S6}-10)$$

3. Weight retention power

The weight retention power scales with matrix size:

$$P_{\text{weight}} = N^2\, P_{\text{weight/per}} \qquad (\text{S6}-11)$$

4.Modulator RF drive

The relevant RF frequency is:

$$f_{\text{RF}} \approx \frac{1+\rho}{2} R_s \qquad (\text{S6}-12)$$

The half-wave drive $V_\pi(f_{\mathrm{RF}})$is increasing with the rise of f_{RF}. The PAM-M constellation factor is:

$$K_{\mathrm{PAM}} = \frac{M+1}{12(M-1)} \qquad (\mathrm{S6}-13)$$

Assuming 50 Ω termination ($R_{\mathrm{term}} = 50\,\Omega$) and optional static driver draw $P_{\mathrm{driver,static/ch}}$, the total modulator power P_{mod} is:

$$P_{\mathrm{mod}} = N\left[\frac{(\kappa V_\pi)^2}{R_{\mathrm{term}}} K_{\mathrm{PAM}} + P_{\mathrm{driver,static/ch}}\right] \qquad (\mathrm{S6}-14)$$

where κ is the driving ratio.

5. Detection and miscellaneous electronics power

In our power model, the detection power P_{det} and control/DSP electronics power P_{misc} are captured with:

$$P_{\mathrm{det}} = N\big(P_{\mathrm{det,static/ch}} + s_{\mathrm{det}} R_s\big),\ P_{\mathrm{misc}} = N(s_{\mathrm{misc}} R_s) \qquad (\mathrm{S6}-15)$$

where R_s is in GBd, $P_{\mathrm{det,static/ch}}$is a rate-independent term (e.g., TIA bias, ADC references), and the slopes $s_{\mathrm{det}}$and s_{misc}have units of W/(GBd·ch). The linear dependence on R_s reflects CMOS dynamic power ($P \sim \alpha C V^2 f$) and bandwidth-driven analog biasing, so that incremental power is approximately proportional to throughput over the operating window. The detection slope s_{det} aggregates analog front-end and data-conversion costs and can be decomposed as $s_{\mathrm{det}} \approx s_{\mathrm{TIA}} + s_{\mathrm{ADC}}$; a convenient bottom-up estimate for the ADC contribution is $s_{\mathrm{ADC}} \approx \mathrm{FOM}_W\, 2^{\mathrm{ENOB}} \mathrm{OSR} \times 10^9$ W/(GBd·ch), using the Walden figure of merit FOM_W(J/conv-step), effective number of bits (ENOB), and oversampling ratio (OSR). The miscellaneous slope s_{misc} bundles clock distribution, PLL/CDR, gearboxes, lane buffers, and lightweight. Parameters used in this work are listed below:

Table S6-1. Parameters used in the energy-per-operation calculations.

Category	Quantity	Symbol	Default	Units
System	Target symbol SNR	$\mathrm{SNR}_{\mathrm{sym,target}}$	30	dB
System	RRC roll-off	ρ	0.20	—
System	OSNR reference bandwidth	RBW	12.5e9	Hz
Laser	Laser wall-plug efficiency	η_{laser}	0.30	—
SOA/mesh	Per-segment gain cap	G_{max}	15	dB
SOA/mesh	Gain margin per segment	h_{dB}	1	dB
SOA/mesh	Spontaneous emission factor	n_{sp}	2.0	—
SOA/mesh	Tail loss	L_{tail}	3	dB
Platform loss	Per-layer loss (SOI)	ℓ_{SOI}	0.40	dB/layer
Platform loss	Per-layer loss (SiN)	ℓ_{SiN}	0.15	dB/layer
Bias/weights	SOA bias (per device)	$P_{\mathrm{SOA,bias}}$	250	mW/device
Bias/weights	Thermal weight (per weight)	$P_{\mathrm{weight/per}}$	18	mW/weight
Modulator	RF termination	R_{term}	50	Ω
Modulator	PAM order	M	256	—
Modulator	Drive ratio	κ	1.0	—

Modulator	Driver static (per channel)	$P_{\text{driver,static/ch}}$	0.00	W/ch
Detection	Static receive-chain (per ch.)	$P_{\text{det,static/ch}}$	10	mW/ch
Detection	Rate slope (per ch.)	s_{det}	1.0	mW/(GBd·ch)
Misc. electronics	Rate slope (per ch.)	s_{misc}	0.5	mW/(GBd·ch)
Constants	Planck constant	h	6.62607015e-34	J·s
Constants	Speed of light	c	2.99792458e8	m/s
Constants	Wavelength	λ	1550	nm
Constants	Optical frequency	ν	c/λ	Hz

Table S6-2. $LiNbO_3$ TW-MZM V_{π}(f)[1]

Frequency (GHz)	V_{π} [V]
1	5.5
5	5.8
10	6.2
20	6.8
30	7.4
38	8.4
40	8.7

6.2 Recurrent depth

This part details the methodology used to compute the maximum recurrent depth N_{rec} of our recurrent photonic processors implemented on Silicon-on-Insulator (SOI) and Silicon Nitride (SiN) platforms. The depth is limited by two independent mechanisms:

1.Phase-noise accumulation (laser phase diffusion over the loop time), and

2.OSNR degradation (ASE noise accumulation due to per-round loss that must be compensated).

For a matrix of size N, we evaluate both limits and take the minimum:

$$N_{\mathrm{rec}}(N) \; = \; \min\left(N_{\phi,\max}(N),\, N_{\mathrm{OSNR},\max}(N)\right) \qquad (\mathrm{S6}-16)$$

1.Phase-noise limited recurrent depth

A single-mode laser with Lorentzian spectrum of full-width $\Delta\nu$ exhibits Wiener phase diffusion with variance that grows linearly in elapsed time t. The canonical result is

$$\mathrm{Va\,r}[\phi(t) - \phi(0)] \; = \; 2\pi\, \Delta\nu\, t \qquad (\mathrm{S6}-17)$$

This follows from the relationship between Lorentzian coherence and a Markov (Wiener) phase process. In coherent detection, the **r**elative phase between the signal and LO matters. If the same source feeds both Tx and LO, the common-mode laser noise largely cancels, and the effective relative linewidth reduces to:

$$\Delta\nu_{\mathrm{rel}} \; = \; \Delta\nu_{\mathrm{sig}} = \Delta\nu_{\mathrm{LO}} \qquad (\mathrm{S6}-18)$$

Each recurrent step takes the round-trip time $T_{\mathrm{rt}} = \tau$. The per-step phase variance is therefore:

$$C_{\phi} \; = \; 2\pi\, \Delta\nu_{\mathrm{rel}}\, \tau \qquad (\mathrm{S6}-19)$$

The error event is a sign-bit flip between two antipodal phase states (0 vs. π) in high-SNR phase readout. Under small Gaussian phase jitter with rms σ_{ϕ}, a flip occurs when the instantaneous phase error exceeds $\pm\frac{\pi}{2}$. Thus

$$P_e^{\mathrm{sign}} \; = \; 2\, Q\left(\frac{\frac{\pi}{2}}{\sigma_{\phi}}\right) \qquad (\mathrm{S6}-20)$$

where $Q(\cdot)$is the standard normal tail probability. Solving for σ_{ϕ}at a target P_e^{sign}gives:

$$\sigma_{\phi,\max} = \frac{\pi/2}{Q^{-1}\left(P_e^{\text{sign}}/2\right)} \quad (S6-21)$$

Variances add over independent steps; after n steps, the accumulated variance is nC_ϕ. Requiring $nC_\phi \leq \sigma^2_{\phi,\max}$yields:

$$N_{\phi,\max} = \left\lfloor \frac{\sigma^2_{\phi,\max}}{C_\phi} \right\rfloor \quad (S6-22)$$

2.OSNR limited recurrent depth

In each round the signal encounters a passive power loss factor A(linear, $A > 1$corresponds to $A_{\text{dB}} > 0$). To maintain the same observation-point signal level, an equivalent gain $\approx A$must be applied. Optical gain injects amplified spontaneous emission (ASE) over the measurement bandwidth $\Delta\nu$. Using a lumped, per-round noise model with a linear noise figure F, the incremental ASE power per round scales as:

$$P_{\text{ASE, round}} \propto F\, h\nu\, (A-1)\, \Delta\nu \quad (S6-23)$$

Here, $h\nu$ is the photon energy, $A - 1$ encodes how much excess gain is needed to compensate for the loss (no loss ⇒ no gain ⇒ no added ASE), $\Delta\nu$ is the noise measurement bandwidth, F absorbs amplifier physics and filter details into a single linear factor. Signal power is held roughly constant round-to-round (by gain), while noise power adds. Therefore, in linear units, we have:

$$\frac{1}{\text{OSNR}(n)} = \frac{1}{\text{OSNR}_0} + K\, n \quad (S6-24)$$

with slope:

$$K = \frac{F\, h\, \nu\, (A-1)\, \Delta\nu}{P_s} \quad (S6-25)$$

Where P_s is the signal optical power at the OSNR measurement point

This is a first-order model that is accurate when the gain stages remain in the linear, unsaturated regime and the per-round ASE is small compared to the signal. For a minimum required OSNR (linear) $\text{OSNR}_{\min}$,

$$N_{\text{OSNR,max}} = \frac{1/\text{OSNR}_{\min} - 1/\text{OSNR}_0}{K} \quad (S6-26)$$

6.3 Normalized roofline model

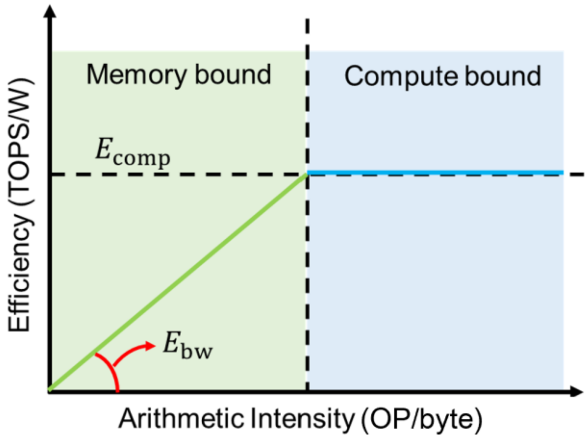

Fig. S6-1 Normalized roofline model schematic. Energy efficiency versus arithmetic intensity. In the memory-bound region, efficiency rises linearly with slope E_{bw}; in the compute-bound region it saturates at the plateau E_{comp}.

We introduce a normalized roofline model to quantify energy efficiency across diverse hardware architectures as a function of arithmetic intensity, as illustrated in Fig. S6-1. This framework enables direct comparison of computational platforms by normalizing performance metrics to power consumption, revealing fundamental efficiency trade-offs in modern processing systems.

The achievable energy efficiency *E(AI)* for any given arithmetic intensity *AI* (operations per byte) follows a piecewise linear relationship bounded by memory and compute constraints:

$$E(AI) = \min\left(E_{\text{comp}}, AI \cdot E_{\text{bw}}\right) \qquad (\text{S6}-27)$$

where E_{comp} represents peak compute efficiency (TOPS/W) and E_{bw} denotes memory bandwidth efficiency (bytes/s/W):

$$E_{\text{comp}} = \frac{\text{Peak TOPS}}{\text{Compute power}} [\text{TOPS/W}] \qquad (\text{S6}-28)$$

$$E_{\text{bw}} = \frac{\text{Peak bytes/s}}{\text{Memoey power}} [\text{B/s/W}] \qquad (\text{S6}-29)$$

This formulation captures the fundamental architectural balance between computational throughput and data movement efficiency.

Unlike conventional architectures that repeatedly transfer intermediate results between memory hierarchies, our system maintains computational states as circulating optical signals—effectively creating "in-flight memory". This architectural innovation eliminates redundant read-write cycles between iterations, enabling direct successive computations within the optical domain. Consequently, the memory bandwidth

efficiency (E_{bw}) increases substantially. For fair comparison, we assume our TPPP employs HBM2e as the memory medium. Note that bandwidth-energy efficiency (E_{bw}) reciprocal represents energy per byte:

$$e_{\mathrm{byte}} = \frac{1}{E_{\mathrm{bw}}}[\mathrm{J/B}] = 8e_{\mathrm{bit}}[\mathrm{J/bit}] \qquad (S6-30)$$

Using this formula and reference[2,3], we derive E_{bw} for both HBM2e and HBM2, which are two widely used memory technologies in modern AI processors.

Details on the *AI* performance and energy efficiency of our TPPP are provided in Supplementary Notes 1, 2, and 6. Table S6-3 lists the parameters of all processors evaluated in Fig. 5k.

Table S6-3 Roofline parameters for evaluated processors

Processors	Compute peak (TOPS/W)	Effective BW slope (GB/s/W)	*AI* knee (ops/byte)
NVIDIA A100[4]	2.08	29.07	71.552
Google TPU v4[5]	1.432	32.051	44.688
TPPP	3.43	536.218	6.397
AMD MI250[6]	0.647	29.07	22.243
AMD MI100[7]	0.308	32.051	9.599
NVIDIA A800[8]	5.196	32.051	162.11

6.4 Operation-matched raw single-pass compute delay

To compare TPPP and A100 at the same abstraction level, we estimate the raw single-pass compute delay required to perform the same number of MAC operations. This comparison excludes system-level overheads, including memory movement, kernel launch, batching, synchronization, optical delay memory, and external control.

For an $N \times N$matrix-vector operation implemented by the MZI mesh,

$$\mathbf{y} = \mathbf{W}\mathbf{x}, \qquad \text{(S6-31)}$$

the required number of multiply-accumulate operations is[9,10]

$$N_{\mathrm{MAC}}(N) = N^2. \qquad \text{(S6-32)}$$

For the A100 reference, we use a serialized Tensor Core tile estimate. An Ampere FP16 MMA tile with shape $m16n8k16$performs[9,10]

$$N_{\mathrm{MAC,tile}} = 16 \times 8 \times 16 = 2048 \qquad \text{(S6-33)}$$

MAC operations. Therefore, the number of serialized Tensor Core tiles needed to match the same operation count is

$$N_{\mathrm{tile}}(N) = \left\lceil \frac{N^2}{2048} \right\rceil. \qquad \text{(S6-34)}$$

If one Tensor Core tile has latency L_{MMA}clock cycles and the A100 clock frequency is f_{clk}, the A100 raw single-pass compute delay is

$$\tau_{\mathrm{A100}}(N) = N_{\mathrm{tile}}(N) \frac{L_{\mathrm{MMA}}}{f_{\mathrm{clk}}}. \qquad \text{(S6-35)}$$

Substituting Eq. S6-34 gives

$$\tau_{\mathrm{A100}}(N) = \left\lceil \frac{N^2}{2048} \right\rceil \frac{L_{\mathrm{MMA}}}{f_{\mathrm{clk}}}. \qquad \text{(S6-36)}$$

This is the A100 raw compute-delay reference plotted in Fig. 5f. It should be interpreted as a serialized arithmetic-delay estimate, not as an optimized GPU runtime. It excludes GPU parallelism, pipelining, memory movement, kernel launch, batching, and scheduling. Similarly, the TPPP curve represents the raw optical propagation delay through the MZI mesh and excludes optical-loop and system-level overheads. Thus, Fig. 5f compares raw single-pass compute delay under the same MAC count.

Supplementary Note 7: Three Forms of Temporal Plasticity

The term temporal plasticity is used in different ways across different fields. In this work, we use it in a broad engineering sense: it refers to the ability of a processor's effective operator to vary from one timestep to the next during operation.

To avoid confusion with stricter usages of the term, this note distinguishes three mechanisms by which a processor's effective behavior can vary in time, and makes explicit which one is demonstrated in this work.

At each timestep k, the processor implements an effective operator $\mathcal{F}_{\Theta_k}$. The system evolves as:

$$\boldsymbol{z}_k = \mathcal{F}_{\Theta_k}(k, \boldsymbol{x}_k, \boldsymbol{s}_k) \qquad \text{(S7}-\text{1)}$$

where $\boldsymbol{x}_k$ is the runtime input, $\boldsymbol{z}_k$ is the current outputs, and $\boldsymbol{s}_k$ denotes all information available to the operator-acquisition rule at timestep k. This state is not restricted to a hidden state in the machine-learning sense. It may include previous states or outputs, measured signals, physical path conditions, device or environmental states, and previously applied operator settings. Θ_k is the configuration of the hardware at timestep k.

Temporal plasticity simply means that:

$$\Theta_k \neq \Theta_{k-1} \qquad \text{(S7}-\text{2)}$$

The key question is therefore not merely whether Θ_k changes in time, but how Θ_k is obtained at each timestep.

We express three commonly used regimes to obtain Θ_k as:

$$\Theta_k = \mathcal{A}_m(k, \boldsymbol{x}_k, \boldsymbol{s}_k; \Omega_m), m \in \{\text{pre}, \text{ana}, \text{data}\} \qquad \text{(S7}-\text{3)}$$

Here, $\mathcal{A}_m$ denotes the operator-acquisition mechanism, and Ω_m specifies the source from which this mechanism is determined. These three regimes have been extensively studied across control theory, signal processing, and machine learning[11–16]:

1. **Predefined temporal plasticity** ($m =$ pre). Ω_{pre} is a designer-specified schedule or rule fixed prior to operation, so that Θ_k evolves along with a predetermined behavior.
2. **Analytical temporal plasticity** ($m =$ ana). Ω_{ana} is an explicitly prescribed analytical, physical, algorithmic, or control-theoretic rule, from which Θ_k is computed online during operation.
3. **Data-driven temporal plasticity** ($m =$ data). Ω_{data} is a data-derived temporal mapping, model, policy, or memory structure learned or constructed from empirical, simulated, calibration, historical, or task-specific data. During operation, Θ_k is obtained from Ω_{data} according to the timestep, input, current state, or other runtime context. This class includes lookup-table retrieval, interpolation over calibrated temporal libraries, learned temporal controllers, and data-trained mappings from context to operator states. This is the regime demonstrated in the present work.

7.1 Predefined temporal plasticity

In predefined temporal plasticity, the operator at each timestep is determined by a rule or schedule specified by the designer before the system runs:

$$\Theta_k = \mathcal{A}_{\text{pre}}(k, \boldsymbol{x}_k, \boldsymbol{s}_k; \Omega_{\text{pre}}) \tag{S7 − 4}$$

Here, Ω_{pre} denotes predefined rules, empirical schedules, lookup tables, or prior design knowledge. In the strict predefined case, the dependence on x_k and s_k is absent. The expression therefore reduces to:

$$\Theta_k = \mathcal{A}_{\text{pre}}(k; \Omega_{\text{pre}}) = \Theta_{\text{pre}}(k) \tag{S7 − 5}$$

Examples include a periodic modulation pattern, a fixed compensation sequence, a hand-designed temporal schedule, or any deterministic sequence of weight settings specified in advance.

The defining property is that the operator trajectory is fixed before runtime data are processed. In the strict predefined case:

$$\frac{\partial \Theta_k}{\partial \boldsymbol{x}_k} = 0, \frac{\partial \Theta_k}{\partial \boldsymbol{s}_k} = 0 \tag{S7 − 6}$$

Equivalently, the entire sequence of operator states could be written into the hardware memory before any runtime input is observed. Predefined temporal plasticity is simple, deterministic, reproducible, and easy to implement. It requires no runtime sensing, no online computation of the operator, and no learned data-derived memory bank. However, it cannot naturally capture task-dependent, input-path-dependent, device-dependent, or environment-dependent regularities unless these effects are explicitly incorporated by the designer.

7.2 Analytical Temporal Plasticity

In analytical temporal plasticity, the operator is computed during operation from an explicitly specified rule:

$$\Theta_k = \mathcal{A}_{\text{ana}}(k, \boldsymbol{x}_k, \boldsymbol{s}_k; \Omega_{\text{ana}}) \tag{S7 − 7}$$

Here, Ω_{ana} denotes an analytical model, physical model, algorithmic update rule, compensation formula, or control law. The operator is not merely read from a fixed predefined schedule. Instead, it is generated by evaluating an explicit rule that may depend on the current timestep, the current input, and the current runtime state.

The runtime state $s_k$may include quantities such as previous outputs, measured signals, environmental conditions, device states, path-selection states, and previous operator settings.

Thus, analytical temporal plasticity covers cases such as:

- an analytical compensation formula for phase drift;
- a physical model that computes modulation states from measured temperature or wavelength shifts;

- an algorithmic rule that updates the operator based on the current state;
- a controller that computes Θ_k from measured outputs or error-related observables;
- a model-based rule that adapts to runtime device or environmental conditions.

In this regime, two different runtime states can lead to different operator values at the same timestep:

$$s_k^{(a)} \neq s_k^{(b)} \Rightarrow \Theta_k^{(a)} \neq \Theta_k^{(b)} \quad (S7-8)$$

Equivalently, the operator may depend explicitly on runtime variables:

$$\frac{\partial \Theta_k}{\partial \boldsymbol{x}_k} \neq 0 \text{ or } \frac{\partial \Theta_k}{\partial \boldsymbol{s}_k} \neq 0 \quad (S7-9)$$

Analytical temporal plasticity provides more flexibility than a fixed schedule. It can adapt to runtime state, measured signals, device drift, environmental changes, or sample-specific conditions, provided that a suitable analytical or algorithmic rule is available. However, it requires an explicit rule that is accurate enough to generate useful operator states. For complex photonic systems, such a rule may be difficult to derive because loss, phase, device nonuniformity, thermal drift, wavelength dependence, path selection, and nonlinear hardware effects can interact in complicated ways.

7.3 Data-driven Temporal Plasticity

Data-driven temporal plasticity refers to the regime in which the source Ω_{data} is constructed from data rather than manually specified as a fixed schedule or derived as a closed-form analytical rule:

$$\Theta_k = \mathcal{A}_{\text{data}}(k, \boldsymbol{x}_k, \boldsymbol{s}_k; \Omega_{\text{data}}) \quad (S7-10)$$

Here, Ω_{data} denotes a data-derived temporal mapping, model, policy, or memory structure learned or constructed from empirical, simulated, calibration, historical, or task-specific data. During operation, Θ_k is obtained from Ω_{data} according to the timestep, input, current state, or other runtime context.

This definition intentionally includes a broad class of data-derived acquisition mechanisms, including lookup-table retrieval, interpolation over calibrated temporal libraries, learned temporal controllers, data-trained mappings from context to operator states, and memory-based retrieval of calibrated operator trajectories. Therefore, data-driven temporal plasticity is not limited to a fixed memory bank. A memory bank is one implementation. More generally, the defining feature is that the operator-acquisition source Ω_{data} is derived from data. Therefore, data-driven temporal plasticity may also exhibit runtime dependence as in $(S7-8)$ and $(S7-9)$. This dependence is learned from the data-derived adaptation rules encoded in Ω_{data}, rather than being imposed by an explicitly prescribed analytical formula.

The present work belongs to the data-driven temporal plasticity regime.

In the linear learning task, as given in Methods: Richardson's iterative method, the runtime input condition is:

$$\mathbf{x}_k = \mathbf{X}_k \quad (S7-11)$$

The system state is:

$$\mathbf{s}_k = \mathbf{I} - \alpha\mathbf{Y} \quad (S7-12)$$

The data-driven adaptation rule therefore gives:

$$\Theta_k = \mathcal{A}_{\text{data}}(k, \mathbf{x}_k, \mathbf{s}_k; \Omega_{\text{data}}) = \mathcal{A}_{\text{data}}(k, \mathbf{X}_k, \mathbf{I} - \alpha\mathbf{Y}; \Omega_{\text{data}}) \quad (S7-13)$$

Here, the adaptation of the effective operator $\mathcal{F}_{\Theta_k}$ is realized through the active correction coefficient $\mathbf{c}_k$, as defined in equation (2) of the main text. Although this coefficient is calibrated before deployment, it should not be interpreted as a hand-designed or purely input-independent temporal schedule. Rather, it is learned from empirical device and environmental data and represented as an interpolation model conditioned on the runtime input condition $\mathbf{X}_k$ and the system state $\mathbf{I} - \alpha\mathbf{Y}$.

In practice, different input channels, optical paths, and hardware configurations can experience different path-dependent losses, device nonidealities, environmental perturbations, and channel-dependent signal skew. The active correction coefficient is therefore selected according to the current timestep, input-selected optical condition, and system state, enabling data-calibrated compensation during the iterative computation. Thus, in the linear mode, Θ_k is ultimately expressed as a data-derived interpolation model based on $\mathbf{X}_k$ and $\mathbf{I} - \alpha\mathbf{Y}$, rather than as a manually prescribed modulation rule.

In the nonlinear mode, the time-varying operator is implemented through data-trained adaptations of the singular-value-domain parameters:

$$\mathbf{S}_{ih}(k) = \mathbf{S}_{ih} + \lambda\Delta\mathbf{S}_{ih}(k) \quad (S7-14)$$

$$\mathbf{S}_{hh}(k) = \mathbf{S}_{hh} + \lambda\Delta\mathbf{S}_{hh}(k) \quad (S7-15)$$

Here, $\mathbf{S}_{ih}$ and $\mathbf{S}_{hh}$ denote the baseline singular-value-domain parameters for the input-to-hidden and hidden-to-hidden transformations, respectively. The terms $\Delta\mathbf{S}_{ih}(k)$ and $\Delta\mathbf{S}_{hh}(k)$ denote timestep-dependent adaptations learned from input-output training data and hidden-state evolution. These adaptations are therefore data-derived rather than manually specified or computed from a closed-form analytical model. The scalar λ controls the strength of the temporal modulation.

The corresponding Θ_k can be written as:

$$\Theta_k = \mathcal{A}_{\text{data}}(k, \mathbf{x}_k, \mathbf{s}_k; \Omega_{\text{data}}) = \mathcal{A}_{\text{data}}(k, v_k, a_{k-1}, \mathbf{W}_{ih}(k), \mathbf{W}_{hh}(k); \Omega_{\text{data}}) \quad (S7-16)$$

where:

$$\mathbf{s}_k = \{a_{k-1}, \mathbf{W}_{ih}(k), \mathbf{W}_{hh}(k)\} \quad (S7-17)$$

$$\mathbf{x}_k = v_k \quad (S7-18)$$

$$\mathbf{W}_{ih}(k) = \mathbf{U}_{ih}\text{diag}(\mathbf{S}_{ih} + \lambda\Delta\mathbf{S}_{ih}(k))\mathbf{V}_{ih}^T \quad (S7-19)$$

$$\mathbf{W}_{hh}(k) = \mathbf{U}_{hh}\mathrm{diag}(\mathbf{S}_{hh} + \lambda\Delta\mathbf{S}_{hh}(k))\mathbf{V}_{hh}^{T} \quad (S7-20)$$

Thus, in the nonlinear mode, temporal plasticity arises because the effective operator changes with timestep through the learned singular-value perturbations:

$$\Theta_k \neq \Theta_{k-1} \mathrm{if} \Delta\mathbf{S}_{ih}(k) \neq \Delta\mathbf{S}_{ih}(k-1) \mathrm{or} \Delta\mathbf{S}_{hh}(k) \neq \Delta\mathbf{S}_{hh}(k-1) \quad (S7-21)$$

This corresponds to data-driven temporal plasticity because the time-varying components of Θ_k, namely $\Delta\mathbf{S}_{ih}(k)$and $\Delta\mathbf{S}_{hh}(k)$, are learned from data rather than predefined by hand or computed online from an explicit analytical rule. Although the learned modulation follows a fixed temporal schedule during inference, its origin is data-driven: it encodes temporal regularities extracted from task-specific input-output data and hidden-state evolution. In this mode, $\Theta_k$ultimately corresponds to a temporally modulated RNN operator, whose effective computation depends on both the runtime input, model parameters and the evolving hidden state.

Together, the linear and nonlinear demonstrations therefore fall within the data-driven temporal-plasticity regime. The linear mode uses a data-calibrated interpolation model to select the active correction coefficient according to runtime input and system state, whereas the nonlinear mode uses data-trained singular-value adaptations to modulate the recurrent operators on top of conventional hidden-state dynamics. In both cases, the time-dependent operator is obtained from data-derived acquisition mechanisms rather than from manually prescribed, purely input-independent schedules.

7.4 Hardware Perspective

From a hardware-implementation perspective, the three regimes share the same essential requirement: the physical processor must implement a time-varying effective operator, updating its configurable degrees of freedom from one timestep to the next. Whether the operator at each step is obtained from a predefined schedule, an analytical rule, or a data-derived source, the hardware must change its tunable state between consecutive timesteps.

The distinction among predefined, analytical, and data-driven temporal plasticity therefore does not lie in the photonic modulation substrate itself. In all three cases the processor requires a mechanism for dynamically changing the implemented operator — through modulator voltages, phase-shifter settings, attenuation states, or other tunable parameters. The regimes differ only in how the control signals are generated: in the predefined case, the time-resolved operator sequence is specified before operation; in the analytical case, it is computed online from an explicit physical, algorithmic, or control-theoretic rule; in the data-driven case, it is obtained from a data-derived mapping, model, policy, or memory structure. In each regime, a set of source parameters feeds a generating mapping that produces the operator at each timestep,

which the hardware then writes into its configuration. A key implication is that any hardware platform capable of demonstrating data-driven temporal plasticity can, in principle, also support the predefined and analytical regimes. Data-driven operation already requires the hardware to realize dynamically changing operators, so once the actuation layer accepts a time-resolved sequence of operator settings, that same layer can be driven by a predefined schedule or an analytical rule. The present demonstration thus establishes a hardware basis for temporal plasticity more generally.

Reference

1. Lithium Niobate Electro-Optic Modulators, Fiber-Coupled (1260 nm - 1625 nm). https://www.thorlabs.com.
2. Moon, K.-I., Son, H.-Y. & Lee, K. Advanced Packaging Technologies in Memory Applications for Future Generative AI Era. in *2023 International Electron Devices Meeting (IEDM)* 1–4 (IEEE, San Francisco, CA, USA, 2023). doi:10.1109/IEDM45741.2023.10413890.
3. O'Connor, M. *et al.* Fine-grained DRAM: energy-efficient DRAM for extreme bandwidth systems. in *Proceedings of the 50th Annual IEEE/ACM International Symposium on Microarchitecture* 41–54 (ACM, Cambridge Massachusetts, 2017). doi:10.1145/3123939.3124545.
4. NVIDIA A100 GPUs Power the Modern Data Center. *NVIDIA* https://www.nvidia.com/en-us/data-center/a100/.
5. TPU v4. *Google Cloud Documentation* https://docs.cloud.google.com/tpu/docs/v4?hl=zh-cn.
6. AMD Instinct™ MI250 Accelerators. *AMD* https://www.amd.com/en/products/accelerators/instinct/mi200/mi250.html.
7. AMD Instinct™ MI100 Accelerators. *AMD* https://www.amd.com/en/products/accelerators/instinct/mi100.html.
8. NVIDIA A800 40GB Active Graphics Card. *NVIDIA* https://www.nvidia.com/en-us/products/workstations/a800/.

9. 1. Introduction — PTX ISA 9.3 documentation. https://docs.nvidia.com/cuda/parallel-thread-execution/.

10. Sun, W., Li, A., Geng, T., Stuijk, S. & Corporaal, H. Dissecting Tensor Cores via Microbenchmarks: Latency, Throughput and Numeric Behaviors. *IEEE Trans. Parallel Distrib. Syst.* **34**, 246–261 (2023).

11. Sutton, R. S. & Barto, A. G. Reinforcement Learning: An Introduction. *IEEE Transactions on Neural Networks* **9**, 1054–1054 (1998).

12. Shalev-Shwartz, S. Online Learning and Online Convex Optimization. *Found. Trends Mach. Learn.* **4**, 107–194 (2012).

13. Ioannou, P. A. & Datta, A. Robust adaptive control: a unified approach. *Proceedings of the IEEE* **79**, 1736–1768 (1991).

14. Mayne, D. Q., Rawlings, J. B., Rao, C. V. & Scokaert, P. O. M. Constrained model predictive control: Stability and optimality. *Automatica* **36**, 789–814 (2000).

15. Leith, D. J. & Leithead, W. E. Survey of gain-scheduling analysis and design. *International Journal of Control* **73**, 1001–1025 (2000).

16. Rugh, W. J. & Shamma, J. S. Research on gain scheduling. *Automatica* **36**, 1401–1425 (2000).
